# Imagine yourself as a little girl…—efficacy and psychophysiology of imagery techniques targeting adverse autobiographical childhood experiences- multi-arm randomised controlled trial

**DOI:** 10.3389/fpsyg.2025.1710963

**Published:** 2026-01-16

**Authors:** Julia Bączek, Stanisław Karkosz, Magdalena Pietruch, Robert Szymański, Jarosław M. Michałowski

**Affiliations:** 1 Laboratory of Affective Neuroscience in Poznan, SWPS University, Warsaw, Poland; 2 Laboratory of Brain Imaging, Nencki Institute of Experimental Biology of the Polish Academy of Sciences, Warsaw, Poland

**Keywords:** imagery rescripting, imagery exposure, memory reconsolidation, prediction error, skin conductance level, salivary alpha-amylase, fear of failure, randomised controlled trial

## Abstract

Fear of failure is often rooted in highly self-critical autobiographical memories that elicit persistent distress and avoidance. Imagery-based interventions aim to reduce the impact of such memories, yet their mechanisms of action remain unclear. In this three-arm parallel group randomised controlled trial, 180 young adults with elevated fear of failure were randomly assigned to imagery exposure, standard imagery rescripting, or imagery rescripting with a 10-min delay designed to disrupt memory reconsolidation. Across four sessions delivered over 2 weeks, outcomes were assessed using self-report measures and physiological markers, with follow-ups at 3 and 6 months. All interventions led to significant and sustained reductions in negative emotions, arousal, and fear of failure, as well as decreased physiological reactivity to autobiographical memories of criticism. Contrary to predictions, delayed rescripting did not show superiority, while planned contrasts suggested more consistent benefits of standard rescripting compared to delayed rescripting and a rebound effect after exposure. Notably, prediction error, operationalised as transient increases in physiological arousal during rescripting, predicted stronger therapeutic change in rescripting but not in exposure. These findings demonstrate that both common therapeutic factors and prediction error contribute to durable improvements in emotional responses to adverse memories, advancing the understanding of mechanisms underlying imagery-based techniques.

**Clinical trials registration:**

ClinicalTrials.gov Identifier: NCT07048756, https://clinicaltrials.gov/ct2/show/NCT07048756.

## Introduction

Adverse childhood experiences, such as criticism, neglect, or harsh responses from caregivers, have been consistently linked to long-term impacts on psychological well-being and quality of life in adulthood (Williams et al., 2022). Among these experiences, the way caregivers respond to a child’s mistakes or failures may play a key role in shaping later emotional and cognitive patterns. One possible consequence is fear of failure – the belief that making mistakes leads to rejection or being perceived as less worthy (Balkis et al., 2024). Research suggests that fear of failure may stem from early relational experiences and social learning processes (Deneault et al., 2022; Peterson et al., 2024).

To address earlier adversities, some therapeutic approaches use imagery-based techniques aimed at modifying the emotional meaning of specific autobiographical memories. Two commonly applied techniques are Imagery Exposure (IE) and Imagery Rescripting (ImRs). Both involve the activation of difficult or distressing memories through imagination, but they are based on different therapeutic assumptions and proposed mechanisms of change.

Imagery exposure involves systematic and controlled repeated mental exposure to distressing events which is suggested to result in gradual weakening of emotional reactivity via habituation (Hamlett et al., 2023). Habituation may allow for increased tolerance of distressing content thanks to a better top-down regulation of aversive memory traces, rather than directly updating these traces (Schiller et al., 2013). Clinical evidence shows that imagery exposure produces significant reductions in emotional distress across anxiety- and trauma-related disorders. Randomised and routine-care studies have demonstrated large and sustained effects of IE, confirming that it is an established and effective intervention for anxiety problems (Hoppen et al., 2024; Roth-Rawald et al., 2023).

During ImRS, an individual recalls a distressing childhood memory and then imagines a more supportive or protective outcome (Arntz and Weertman, 1999; Morina et al., 2017). This process was proposed to alter the emotional significance of distressing memories and associated beliefs about the self, others, and the world (Woelk et al., 2022).

Clinical research supports the efficacy of Imagery Rescripting (ImRs) in reducing emotional distress (Woelk et al., 2022; Veale et al., 2015). However, the precise mechanisms underlying its effectiveness remain unclear (Siegesleitner et al., 2020; Kunze et al., 2017). It is hypothesized that ImRs not only reduces emotional responses but also alters the affective meaning of memories. It leads to the hypothesis that the mechanisms of change in ImRs may be memory reconsolidation and/or prediction error, both rooted in neurocognitive models of emotional learning.

Memory reconsolidation is a biological process in which reactivated memories become temporarily labile and modifiable before reconsolidating into long-term storage (Nader et al., 2000; Arntz et al., 2013). Interventions applied during a specific time interval -referred to as the reconsolidation window, starting approximately 10 min after memory activation- can disrupt reconsolidation and induce lasting changes in the original memory trace (Schiller et al., 2013; Germeroth et al., 2017; Agren et al., 2017).

Experimental studies have shown that providing new, non-threatening information in the reconsolidation window during threat exposure eliminates conditioned fear responses rather than merely suppressing them (Schiller et al., 2013). While these findings remain debated (Chalkia et al., 2020), successful applications of disruption of reconsolidation-based interventions have been demonstrated in various domains, including spider phobia (Agren et al., 2017), and imaginal exposure (Agren et al., 2017). Using the reconsolidation window for modifications of aversive memory traces in psychotherapeutic contexts remains a promising yet under-examined area (Bui and Milton, 2023).

Another mechanism increasingly discussed in the context of psychotherapy (also ImRs) is prediction error (PE) – the discrepancy between an individual’s expectation regarding a real (or imagined) and actual ending of events. PE is central to learning theory and expectation violations are thought to promote memory updating (Sinclair et al., 2021; Exton-McGuinness et al., 2015). ImRs may elicit PE by introducing unexpected elements – such as a protective figure in a previously threatening memory – thereby enhancing emotional relearning (Dibbets and Arntz, 2016; Siegesleitner et al., 2019).

Although a few studies have explored the potential role of reconsolidation in therapeutic memory change (Strohm et al., 2021), none have directly compared reconsolidation and its disruption, nor systematically examined the role of prediction error in Imagery Rescripting (ImRs) – particularly in the context of personalised autobiographical memories involving criticism, a theme frequently encountered in clinical work.

To address this gap, we conducted a randomised trial including three active conditions: Imagery Exposure (IE), standard Imagery Rescripting (ImRs), and delayed Imagery Rescripting (ImRs-DSR), in which a 10-min interval between memory reactivation and intervention was introduced to interfere with the reconsolidation process. Each intervention session targeted the same autobiographical memory of parental criticism. This design allowed us to investigate whether disrupting reconsolidation alters the emotional and physiological effects of ImRs, and to test the role of prediction error in therapeutic memory change.

We included participants with high levels of fear of failure - a factor often related to memories of social evaluation (Tariq et al., 2021). We assessed outcomes using self-report questionnaires as well as objective physiological indicators – salivary alpha-amylase (sAA) and skin conductance level (SCL). We also applied behavioral research procedures to test how general and stable the treatment effects were. We did this by evaluating the “renewal effect” – the return of fear in a setting different to the one used during treatment (Broomer and Bouton, 2023), such as an unfamiliar room. To measure the durability of change, we included recall (procedure testing spontaneous recovery, which is delayed return of fear in the same room where the treatment was delivered) and reinstatement (return of fear after re-exposure to a fear-related cue). We tested whether the benefits of each intervention lasted over time. To assess generalisation, we used participants’ autobiographical memories not included in the therapeutic intervention, which we refer to as past and future scenarios of aversive events.

## Hypotheses

### Hypothesis 1 (treatment short-term efficacy—direct effects)

Participants in all three conditions (IE, ImRs, ImRs-DSR) will show a reduction in emotional and physiological responses to the treated autobiographical criticism scenario following the intervention. However, this reduction will be stronger in the rescripting conditions (ImRs and ImRs-DSR) compared to IE, and strongest in ImRs-DSR due to the hypothesized enhancement of memory updating through reconsolidation disruption.

Specifically, after undergoing ImRs-DSR participants are expected to show the most pronounced reduction in fear of failure as well as lowest skin conductance levels (SCL) and subjective emotional distress (e.g., fear, sadness, guilt) for their treated autobiographical criticism scenarios, and highest increase in positive affect (e.g., relief, calmness), compared to the other two conditions.

### Hypothesis 2 (stability and long-term effects)

Treatment effects observed post-intervention will be maintained over time (3- and 6-month follow-ups) in all groups, but more strongly in ImRs and ImRs-DSR compared to IE. The ImRs-DSR condition is expected to show the highest stability of both subjective and physiological treatment gains, including resistance to reinstatement (i.e., return of emotional reactivity after hotspot reactivation).

### Hypothesis 3 (generalisation)

Treatment effects will generalise beyond the directly targeted memory to non-treated autobiographical memories (i.e., past and future criticism scenarios), as well as to novel contextual settings (i.e., renewal).

Participants in the ImRs and ImRs-DSR groups are expected to show greater generalisation of emotional regulation (i.e., lower physiological and subjective reactivity) than participants in the IE group. The ImRs-DSR group is expected to exhibit the broadest generalisation due to deeper memory updating.

### Hypothesis 4 (prediction error)

Participants in both rescripting conditions (ImRs and ImRs-DSR) will exhibit greater prediction error (PE) during the intervention phase than those in the IE condition, due to the unexpected positive transformation of the memory scene (see Prediction Error section).

Higher PE is also expected to be associated with greater treatment effects (i.e., greater reductions in post-intervention physiological arousal and subjective distress). PE levels are expected to be comparable between ImRs and ImRs-DSR, as the rescripting content is identical.

## Methods

### Participants

Participants were recruited between June 2020 and June 2022 via online advertisements, social media, and university mailing lists. A total of 6,349 individuals completed the initial online prescreening survey. Of these, 180 participants met the eligibility criteria following clinical interviews and were randomly assigned to one of three experimental conditions:

- Imagery Exposure (IE) (*n* = 60),
- Imagery Rescripting (ImRs) (*n* = 60),
- Imagery Rescripting with a 10-min reconsolidation window (ImRs-DSR) (*n* = 60)

**Figure 1:**
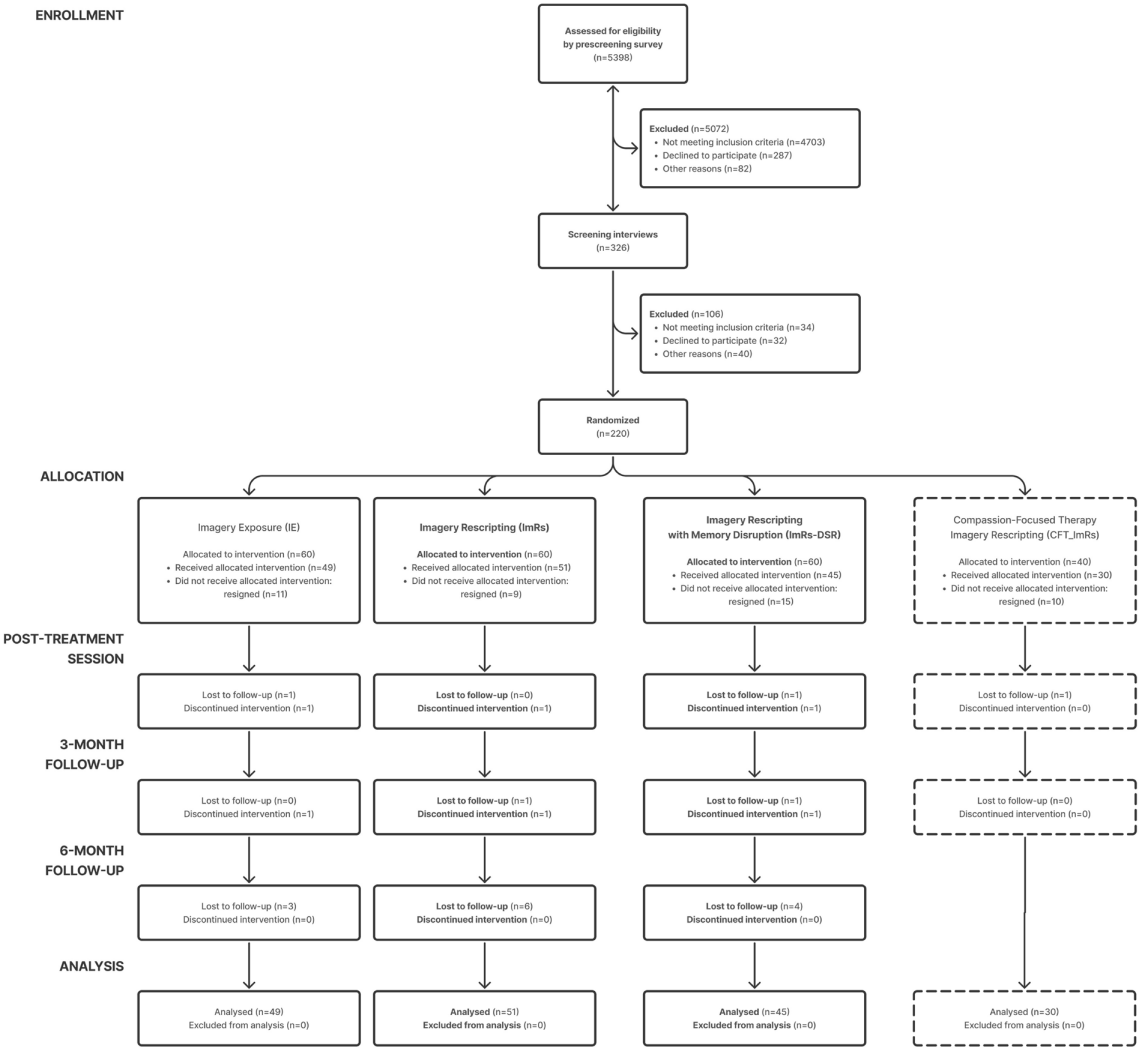
CONSORT diagram.

Of the 180 participants in the three conditions, 145 participants that completed at least one time point, were included in the final analyses (IE: 49, ImRs: 51, ImRs-DSR: 45). The full participant flow is depicted in the CONSORT diagram (see Figure 1). Eligible participants were between 18 and 35 years of age, scored at least 1 SD above the mean on the Performance Failure Appraisal Inventory (PFAI; Conroy et al., 2002; Polish adaptation: Golińska, 2017), and were not undergoing psychotherapy or psychopharmacological treatment at the time of the study. Additional exclusion criteria included a history of sexual abuse or severe childhood maltreatment, current affective or anxiety disorders, personality disorders, psychotic symptoms, active suicidality, or substance use disorders.

The inclusion and exclusion criteria were assessed in two stages: first, through an online screening survey with self-report measures, and second, via a clinical interview conducted by CBT-trained clinicians. The diagnostic assessment included the Mini-International Neuropsychiatric Interview (M.I.N.I. 5.0.0; Sheehan et al., 1998) and the Structured Clinical Interview for DSM-5 Personality Disorders (SCID-5-PD; APA, 2013).

Demographic characteristics were similar across groups. Participants were primarily university students and young female adults, with an average age of approximately 24 years. No significant baseline differences were observed across conditions in terms of age, sex, education level, employment status, or fear of failure scores, however it must be noted that women were overrepresented in the study (see Table 1).

**Table 1:** Participants’ demographic characteristics and screening scores.

Characteristic	Group			*p-value*
	ImRs-DSR	ImRs	IE	
	(*N* = 45)	(*N* = 51)	(*N* = 49)	
Sex, No. (%)				
Female	35 (78%)	42 (82%)	42 (86%)	0.478
Male	10 (22%)	9 (18%)	6 (12%)	
Prefer not to say/different	0 (0%)	0 (0%)	1 (2%)	
Age, Mean (SD)	24.53 (3.75)	24.25 (4.35)	24.04 (3.80)	0.836
Employment status, No. (%)				
Employed	30 (67%)	33 (65%)	29 (59%)	0.515
Unemployed	8 (18%)	5 (10%)	6 (12%)	
Other	7 (16%)	13 (25%)	14 (29%)	
University student status, No. (%)				
Student	31 (69%)	33 (65%)	32 (65%)	0.899
Not a student	14 (31%)	18 (35%)	17 (35%)	
Highest level of education, No. (%)				
General secondary school	21 (47%)	24 (47%)	25 (52%)	0.845
University degree (bachelor’s or higher)	21 (47%)	22 (43%)	21 (44%)	
Vocational education	3 (7%)	5 (10%)	2 (4%)	
Fear of failure, Mean (SD)	118.74 (11.32)	120.68 (9.15)	120.15 (9.18)	0.629

*A priori* power analysis using G*Power indicated that at least 51 participants per group were needed to detect medium-sized effects (Cohen’s *d* = 0.50) with 80% power at *α* = 0.05. To accommodate an expected dropout rate of up to 25%, the recruitment target was set at 68 participants per condition. Ultimately, 145 participants were enrolled across the three main arms (ImRs, ImRs-DSR, IE). Dropout was lower than expected (≈8% of 145), and 145 were included in analysis.

### Study design

This study was conducted as a randomised controlled superiority trial with a between-subjects design and three experimental conditions:

- Imagery Rescripting (ImRs)
- Imagery Rescripting with a 10-min reconsolidation break (ImRs-DSR), designed to disrupt memory reconsolidation
- Imagery Exposure (IE), serving as an active control condition involving emotional imagery without therapeutic modification

**Figure 2:**
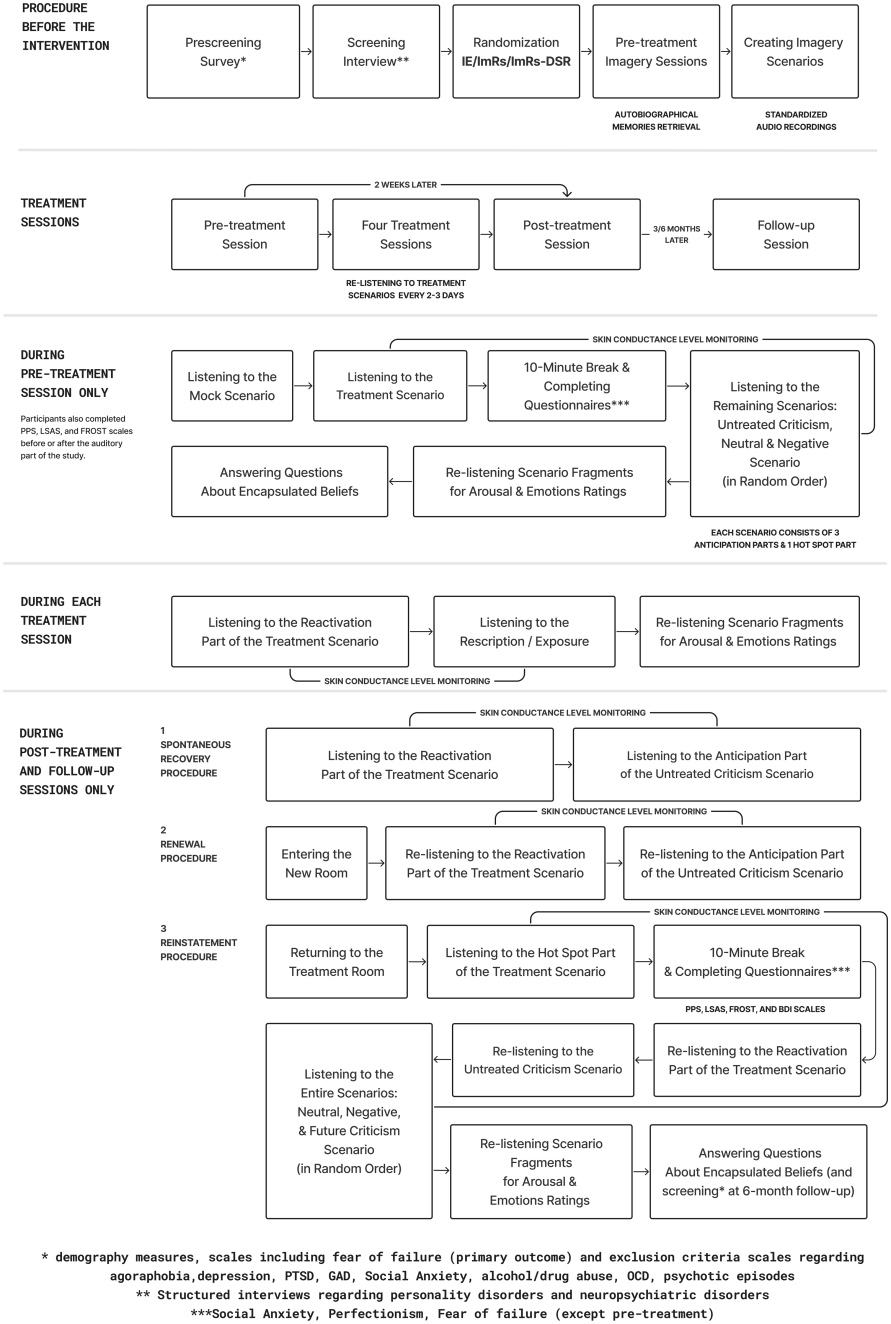
Study design overview.

Participants were randomly assigned to one of the three groups using computer-generated block randomisation. Allocation was performed by researchers not involved in recruitment or data collection. A double-blind procedure was implemented where feasible: participants and therapists conducting preparatory interviews were blind to condition. All participants listened to audio recordings of autobiographical scenarios, and only the content of the therapeutic segment differed between conditions. However, experimenters delivering the interventions could not be fully blinded, due to structural differences between groups (e.g., presence of a 10-min delay in ImRs-DSR). To minimise potential bias, all scenarios were pre-scripted, pre-recorded, and presented in a standardised format across sessions. A detailed description of the procedure may be found in the Appendix, see Figure 2 and Table 2 for the study design and schedule.

**Table 2:** Schedule.

Study phase	Procedure	Details	Enrollment	Pre-Treatment	Intervention 1	Intervention 2	Intervention 3	Intervention 4	Post-treatment				3-month follow-up				6-month follow-up			
									Recall	Renewal	Reinstatement	After	Recall	Renewal	Reinstatement	After	Recall	Renewal	Reinstatement	After
Eligibility	Exclusion Criteria (BDI, YBOCS, AUDIT, DAST, DSM-5 Dimensional Scales)	Yale-Brown Obsessive-Compulsive Scale; Beck Depression Inventory DSM-5 Dimentsional Scales (GAD, Social Phobia, Panic, Agoraphobia, PTSD) Alcohol Use Disorders Identification Test, Drug Abuse Screening Test	X																	X
	Mini International Neuropsychiatric Interview (MINI)	Psychiatric disorders (diagnostic interview)	X																	
	SCID-5-PD (Personality Disorders)	SCID-5-PD (Personality Disorders)	X																	
	Informed Consent	Informed Consent	X																	
Assessment	LSAS	Liebowitz Social Anxiety Scale		X								X				X				X
	FROST	Frost Multidimensional Perfectionism Scale		X								X				X				X
	PPS	Pure Procrastination Scale		X								X				X				X
	Ratings of emotions / arousal / valence / focus / immersion	All/Treated (Intervention sessions)		X	X	X	X	X				X				X				X
Scenario Development	Imagery Interview	Autobiographical memory collection; scenarios development	X																	
Eligibility /Assesment	PFAI (Fear of Failure)	Fear of failure (primary outcome)	X						X	X	X									X
Scenario Presentation	Treated Scenario – Anticipation	Anticipation Phase		X	X	X	X	X	X	X	X	X	X	X	X	X	X	X	x	x
	Untreated Scenario – Anticipation	Anticipation Phase		X					X	X	X	X	X	X		X	X	X		X
	Future Scenario – Anticipation	Anticipation Phase									X	X			X	X		X	X	X
	Negative Scenario – Anticipation	Anticipation Phase		X					X	X	X	X	X	X		X	X	X		X
	Neutral Scenario – Anticipation	Anticipation Phase		X							X	X			X	X			X	X
	Treated Scenario – Hotspot	Hotspot Phase		X							X	X			X	X			X	X
	Untreated Scenario – Hotspot	Hotspot Phase		X								X				X				X
	Future Scenario – Hotspot	Hotspot Phase		X							X	X			X	X			X	X
	Negative Scenario – Hotspot	Hotspot Phase		X							X	X			X	X			X	X
	Neutral Scenario – Interaction	Hotspot Phase		X							X									
Intervention (Scenario Presentation)	Therapeutic Scenario Playback (ImRs/IE/ImRs-DSR)	Treated			X	X	X	X				X				X				X
Physiological	sAA (Baseline/after Hotspot of treated scenario presentation)	Treated		X							X									
	Skin Conductance Level	All	X	X	X	X	X	X	X	X	X		X	X	X		X	X	X	

The study involved eight assessment time points per participant, spread across approximately 6 months:

1. Pre-treatment baseline,
2. Four intervention sessions over two weeks,
3. Post-treatment assessment,
4. Follow-up assessments at 3 and 6 months post-treatment.

At each assessment, participants engaged with personalised, audio-guided imagery scenarios, created based on the autobiographical material collected during preparatory interviews and recorded by the psychotherapist assigned to the participant. One scenario (a past criticism memory) was selected as the targeted treatment scenario, while additional scenarios (untreated past criticism, future criticism, neutral, and negative) were used to assess generalisation and specificity of effects. For the sake of clarity, the present manuscript reports results only for criticism-related scenarios (treated, untreated, and future), as these were central to the study hypotheses and intervention rationale.

In addition to baseline listening and imagery of scenarios, the study incorporated behavioral paradigms adapted from basic learning research to test the stability and relapse vulnerability of treatment effects. These included Recall, Renewal, and Reinstatement (see below for further elaboration). These procedures were administered at post-treatment, 3-month, and 6-month follow-ups and applied to both the treated and untreated scenarios.

The study was reviewed and approved by the Ethics Committee of the Faculty of Psychology and Law, SWPS University in Poznań (approval no. 2019-03-10). All participants provided written informed consent and received up to 830 PLN (approximately ~224 USD) for full participation.

Note on parallel condition: After study initiation, additional funding was obtained to conduct a fourth condition involving Compassion-Focused Imagery Rescripting (CFT-ImRs). This arm followed a procedure analogous to the other groups but was not included in the current manuscript due to its smaller sample size, lack of long-term follow-ups, and its distinct therapeutic rationale (focus on self-compassion). We mention it here to maintain transparency regarding the full study design.

### Imagery scenarios and memory reactivation task

Before treatment, each participant took part in two imagery sessions during which a cognitive-behavioral therapist (CBT) asked him/her to recall three events of being criticized for failures and/or lack of achievements (two past-related and one future-related), one neutral personal event, and one negative event that was not related to failure and criticism. Each imagery session involved only recalling and describing the situation, but no actual intervention. Therapists were supervised, and the recorded imagery sessions were randomly listened to by the supervisor of the experiment as a quality assurance procedure.

Using the information from the two imagery sessions, the following imagery scenarios were developed for each participant: 2 past criticism scenarios, one negative scene without criticism (e.g., an accident), one neutral scenario (e.g., shopping), and one future criticism scenario (e.g., criticism at work). One scenario of past criticism was complemented with a scene of rescripting. Each scenario followed the same framework (see exemplary scenarios in Appendix A) and was audio recorded using the voice of the same therapist who performed the diagnostic and imagery sessions. Each scenario followed a four-part structure, comprising an “anticipation” phase (three segments) and a hotspot phase (the emotionally intense moment in criticism memories, or interaction in other scenarios):

#### Anticipation phase

1. Self-Imagery: Description begins with a standardised phrase such as “Imagine yourself as a [age]-year-old girl/boy….” The participant is referred to by their own name as they appear in the memory.
2. Surroundings Description: The setting is described in the present tense with sensory details (e.g., “You hear...,” “You see...,” “You can smell...”) to enhance immersion and evoke vivid mental imagery.
3. Person (Critic): A depiction of the person interacting with the participant, typically focusing on a parent or parental figure in the case of criticism-related memories (or other person in non-criticism scenario).

#### Hotspot phase

1. Critical Interaction (Hotspot): A detailed narration of the most emotionally intense part of the memory. In the treatment condition, this is the moment of criticism that is later targeted by rescripting. In neutral or negative control conditions, it depicts a routine, emotionally neutral interaction.

**Figure 3:**
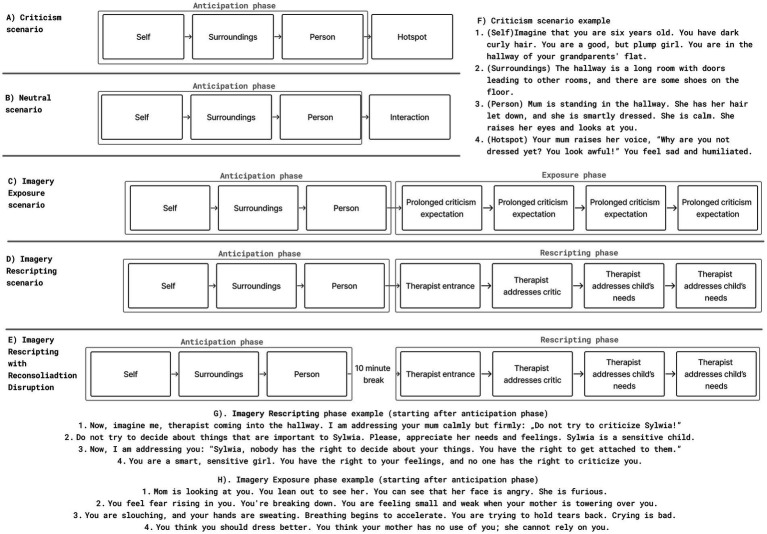
Imagery scenarios framework and examples **(A)** Framework of criticism scenario **(B)** Framework of neutral scenario **(C)** Framework of Imagery Exposure scenario **(D)** Framework of Imagery Rescripting **(E)** Framework of Imagery Rescripting with Reconsolidation Disruption Scenario **(F)** Criticism scenario example (anticipation + hotspot) **(G)** Imagery Rescripting scenario example **(H)** Imagery Exposure scenario example.

This structured scenario framework was used in the Memory Reactivation Task (Strohm et al., 2021), during which participants listened to the audio-recorded scripts and were instructed to imagine each scene as vividly as possible, as if it were occurring in the present moment (See Figure 3 and Appendix for scenario framework and examples).

As mentioned above, we decided to treat the hotspot as an unconditioned stimulus (UCS), similar to the previous study performed by Siegesleitner et al. (2020). In our study, the experience of criticism was considered as the one eliciting automatic aversive responses and having the potential to change the meaning of originally neutral stimuli (Ehlers et al., 2004). For example, recollection of the classroom can have a neutral connotation of everyday classes, or even a positive one, when associated with friends. However, once the criticism (hotspot) takes place, neutral scenery begins to be associated with the aversive experience. This poses some difficulty, as in psychotherapy hotspot is often presented along anticipation (i.e., “late” rescripting, see Discussion). However, considering the hotspot as UCS in our experiment required us to separate it from the rest of the scenario for the sake of the Reinstatement procedure. Furthermore, this translation from behavioral studies has some limitations that pose difficulty in later interpretation, for example, criticism not only evokes fear but also more complex emotions such as guilt or shame (Grey et al., 2001). Yet, we aimed to optimise our research, by treating Hotspot as UCS, which allowed conducting behavioral procedures such as reinstatement. We believe that both behavioral researchers can benefit from testing procedures on more ecologically valid stimuli, as well as psychotherapeutic research can benefit challenging interventions in a laboratory setting using verified behavioral procedures.

### Interventions

During the 2 weeks between pre- and post-treatment, participants participated in four imagery intervention sessions, in which they listened to an audio-recorded treatment scenario. The treatment scenario started in the same way as one of the criticism scenarios, with the anticipation phase. However, the imagery of the critic, instead of the hotspot, was followed by the intervention part. In Imagery Rescripting conditions:

1. The therapist enters the scene and prevents the criticism
2. The therapist addresses a critic and points out the child’s needs
3. The therapist addresses the child and acknowledges their needs
4. The therapist suggests to the child to perform an activity that would meet their needs (see Figure 3 and Appendix A).

The rescripting part was presented immediately after the imagery of the critic (ImRs group; see Figure 3D) or after a 10-min break, to provide intervention in the reconsolidation disruption window (ImRs-DSR group; See Figure 3E). During the break, subjects were watching a neutral documentary (Schiller et al., 2013).

In the Imagery Exposure (IE) condition, the treated criticism scenario continued without rescripting but four additional segments were inserted to promote focused exposure. After the appearance of the critic, participants were instructed to focus on their bodily sensations, expectations, and on the image of the critic (see Figure 3). This condition aimed to facilitate emotional processing through focused exposure without altering the original memory content.

### Recall, renewal, and reinstatement procedures

At pre-treatment, participants listened to scenarios of criticism, neutral, and negative situations (see Memory Reactivation Task & Imagery Scenarios). At post-treatment and follow-up sessions, criticism scenarios were presented using behavioral procedures: recall (listening to scenarios in the familiar treatment room), renewal (listening to scenarios in an unknown room), and reinstatement (back to the original room, listening to the hotspot and then scenarios) (see Appendix for further elaboration).

### Prediction error

In the present study, prediction error (PE) was conceptualised as a moment of surprise – when the content of the scenario unfolded differently than what might have been expected by the participant. In the rescripting conditions (ImRs and ImRs-DSR), criticism was replaced by a positive therapist intervention, potentially violating participants’ expectations. As no self-reported ratings of surprise were collected, PE was not measured directly. Instead, it was operationalised indirectly through the recording of skin conductance level (SCL) as a physiological index of arousal. An increase in SCL observed immediately after the expected moment of criticism - which, according to the scenario structure, was supposed to occur but was replaced by the therapist’s intervention - may indicate a reaction associated with a violation of expectation. A similar arousal pattern has been described in studies on emotional learning and memory updating, where prediction error is linked to transient increases in physiological activity (e.g., Stemerding et al., 2022).

### Outcome measures

#### Primary outcome measures

Primary outcomes were selected to evaluate the emotional and physiological effects of the interventions.

Self-reported fear of failure was assessed using the Performance Failure Appraisal Inventory (PFAI; Conroy et al., 2002; Polish adaptation: Golińska, 2017), a 25-item scale measuring beliefs about the aversive consequences of failure. This measure served as a primary indicator of cognitive-affective change following the intervention.

Physiological reactivity was captured through two complementary indicators:

Skin Conductance Level (SCL): SCL was recorded during imagery exposure to experimental scenarios using Biopac Systems (MP160 EDA-MRI; sampling rate: 2000 Hz; Ag/AgCl electrodes placed on the index and middle finger of the non-dominant hand). Signals were resampled to 1,000 Hz, smoothed (median over 100 samples), and filtered with a high-pass 1 Hz filter. Baseline SCL was calculated from the 3 s preceding the first scenario fragment. Normalised reactivity was computed as (Sugimine et al., 2020):


$$\text{SCLchange}=100\times ({\mathrm{SCL}}_{\text{stim}}-{\mathrm{SCL}}_{\text{baseline}})/{\mathrm{SCL}}_{\text{baseline}}.$$

For analyses, SCL was extracted separately for anticipation and hotspot (emotional peak) phases. SCL is a well-validated marker of sympathetic nervous system arousal and emotional activation (Dawson et al., 2007).

Salivary alpha-amylase (sAA): sAA was used as a non-invasive marker of acute sympathetic-adrenal-medullary (SAM) activation. Saliva samples were collected using cotton rolls or Salivette collection tubes, chewed for 1 min. Samples were obtained immediately before and after the scenario presentation, stored at 4 °C, and later analysed at the Institute of Human Genetics (Polish Academy of Sciences). sAA is considered a reliable biomarker of short-term physiological stress responses (Nater and Rohleder, 2009).

Subjective emotional response was also included as a primary outcome: Participants rated each scenario fragment on valence, arousal, immersion, and basic emotions (sadness, guilt, fear, anger, disgust) using a 9-point Likert scale.

#### Secondary outcome measures

Secondary outcomes included a broader range of self-report measures assessing comorbid traits and symptoms, collected for exploratory purposes and to contextualise individual differences in treatment response. These were:

- Beck Depression Inventory-II (BDI-II; Beck et al., 1996; Zawadzki et al., 2009) – depressive symptoms,
- Liebowitz Social Anxiety Scale (LSAS; Liebowitz, 1987) - social anxiety,
- Frost Multidimensional Perfectionism Scale (Frost MPS; Frost et al., 1990; Piotrowski and Bojanowska, 2021) - trait perfectionism,
- Pure Procrastination Scale (PPS; Steel, 2007; Polish translation: Stępień and Cieciuch, 2013) – procrastination tendencies.

All questionnaires were administered at pre-treatment and post-treatment. LSAS, PPS, and Frost MPS were additionally repeated at 3-month and 6-month follow-ups (Table 3).

**Table 3:** ITT analysis intention-to-treatment analysis results.

Analysis	Time			Group			Time x Group		
	*F*	*p*	Effect Size (η^2^p)	*F*	*p*	Effect Size (η^2^p)	*F*	*p*	Effect Size (η^2^p)
SCL anticipation treated criticism recall	57.750 (2.537,342.519)	<0.001	0.3	1.529 (2,135)	0.221	0.022	1.972 (5.074,342.519)	0.081	0.028
SCL anticipation treated criticism reinstatement	98.901 (2.112,285.096)	<0.001	0.423	2.347 (2,135)	0.100	0.034	1.050 (4.224,285.096)	0.384	0.015
SCL anticipation treated criticism renewal	108.084 (2.327,314.189)	<0.001	0.445	1.948 (2,135)	0.147	0.028	1.087 (4.655,314.189)	0.366	0.016
SCL hotspot treated criticism reinstatement	18.160 (2.597,35.546)	<0.001	0.119	3.045 (2,135)	0.051	0.043	1.684 (5.193,35.546)	0.135	0.024
SCL anticipation untreated criticism recall	3.708 (2.806,375.986)	0.014	0.027	0.255 (2,134)	0.775	0.004	0.855 (5.612,375.986)	0.522	0.013
SCL anticipation untreated criticism reinstatement	0.861 (2.855,382.552)	0.457	0.006	1.348 (2,134)	0.263	0.02	0.791 (5.710,382.552)	0.571	0.012
SCL anticipation untreated criticism renewal	0.704 (2.972,398.307)	0.549	0.005	3.105 (2,134)	0.048	0.044	0.743 (5.945,398.307)	0.614	0.011
SCL anticipation future criticism	35.541 (1.488,184.558)	<0.001	0.223	2.155 (2,124)	0.120	0.034	0.783 (2.977,184.558)	0.504	0.012
SCL hotspot future criticism	52.141 (1.285,159.401)	<0.001	0.296	3.123 (2,124)	0.048	0.048	1.236 (2.571,159.401)	0.298	0.02
Treated criticism anticipation arousal	3.159 (1.948,27.766)	<0.001	0.178	0.580 (2,139)	0.561	0.008	0.441 (3.896,27.766)	0.774	0.006
Treated criticism anticipation disgust	24.085 (2.033,282.643)	<0.001	0.148	1.309 (2,139)	0.273	0.018	0.836 (4.067,282.643)	0.505	0.012
Treated criticism anticipation fear	82.538 (1.795,249.478)	<0.001	0.184	1.127 (2,139)	0.327	0.008	1.116 (3.590,249.478)	0.347	0.005
Treated criticism anticipation guilt	76.635 (1.557,216.363)	<0.001	0.355	1.718 (2,139)	0.183	0.024	1.764 (3.113,216.363)	0.153	0.025
Treated criticism anticipation sadness	101.711 (1.587,22.644)	<0.001	0.423	0.510 (2,139)	0.601	0.007	3.409 (3.175,22.644)	0.016	0.047
Treated criticism reactivation anger	36.818 (2.095,291.206)	<0.001	0.209	0.911 (2,139)	0.404	0.013	0.732 (4.190,291.206)	0.576	0.01
Treated criticism hotspot anger	29.513 (1.989,276.470)	<0.001	0.175	0.933 (2,139)	0.396	0.013	1.139 (3.978,276.470)	0.338	0.016
Treated criticism hotspot arousal	18.481 (2.047,284.506)	<0.001	0.117	1.400 (2,139)	0.250	0.02	2.103 (4.094,284.506)	0.079	0.029
Treated criticism hotspot disgust	3.937 (2.233,31.350)	<0.001	0.182	1.499 (2,139)	0.227	0.021	0.797 (4.465,31.350)	0.540	0.011
Treated criticism hotspot fear	7.831 (2.040,283.491)	<0.001	0.338	0.187 (2,139)	0.830	0.003	0.621 (4.079,283.491)	0.651	0.009
Treated criticism hotspot guilt	83.091 (2.102,292.151)	<0.001	0.374	0.781 (2,139)	0.460	0.011	0.607 (4.204,292.151)	0.666	0.009
Treated criticism hotspot sadness	106.990 (2.220,308.537)	<0.001	0.435	0.373 (2,139)	0.689	0.005	0.336 (4.439,308.537)	0.871	0.005
Future anticipation anger	154.739 (1.529,197.211)	<0.001	0.545	3.179 (2,129)	0.045	0.047	0.804 (3.058,197.211)	0.495	0.012
Future anticipation arousal	36.434 (2.092,269.814)	<0.001	0.22	1.213 (2,129)	0.301	0.018	0.862 (4.183,269.814)	0.491	0.013
Future anticipation disgust	66.927 (1.561,201.351)	<0.001	0.342	4.814 (2,129)	0.010	0.069	1.254 (3.122,201.351)	0.291	0.019
Future anticipation fear	73.228 (1.669,215.362)	<0.001	0.362	0.423 (2,129)	0.656	0.007	0.384 (3.339,215.362)	0.786	0.006
Future Anticipation Guilt	266.145 (1.789,23.746)	<0.001	0.674	1.333 (2,129)	0.267	0.02	1.759 (3.577,23.746)	0.145	0.027
Future anticipation sadness	413.148 (1.858,239.714)	<0.001	0.762	1.849 (2,129)	0.162	0.028	1.631 (3.716,239.714)	0.171	0.025
Future criticism hotspot anger	11.410 (2.320,299.282)	<0.001	0.081	1.168 (2,129)	0.314	0.018	1.291 (4.640,299.282)	0.270	0.02
Future hotspot arousal	8.960 (2.247,289.811)	<0.001	0.065	0.389 (2,129)	0.679	0.006	1.030 (4.493,289.811)	0.397	0.016
Future hotspot disgust	7.824 (2.351,303.282)	<0.001	0.057	3.578 (2,129)	0.031	0.053	0.411 (4.702,303.282)	0.831	0.006
Future hotspot fear	29.787 (2.195,283.209)	<0.001	0.188	0.229 (2,129)	0.795	0.004	1.262 (4.391,283.209)	0.283	0.019
Future hotspot guilt	4.302 (2.283,294.566)	<0.001	0.238	0.444 (2,129)	0.642	0.007	0.660 (4.567,294.566)	0.640	0.01
Future hotspot sadness	67.590 (2.025,261.216)	<0.001	0.344	0.430 (2,129)	0.652	0.007	0.680 (4.050,261.216)	0.608	0.01
Untreated anticipation anger	59.576 (1.978,274.881)	<0.001	0.3	1.067 (2,139)	0.347	0.015	1.245 (3.955,274.881)	0.292	0.018
Untreated anticipation arousal	37.047 (1.923,267.233)	<0.001	0.21	0.506 (2,139)	0.604	0.007	0.541 (3.845,267.233)	0.698	0.008
Untreated anticipation disgust	38.278 (2.054,285.505)	<0.001	0.216	2.311 (2,139)	0.103	0.032	0.999 (4.108,285.505)	0.410	0.014
Untreated anticipation fear	115.634 (1.666,231.514)	<0.001	0.454	0.595 (2,139)	0.553	0.008	0.485 (3.331,231.514)	0.713	0.007
Untreated anticipation guilt	132.129 (1.559,216.763)	<0.001	0.487	1.677 (2,139)	0.191	0.024	0.400 (3.119,216.763)	0.760	0.006
Untreated anticipation sadness	168.716 (1.609,223.649)	<0.001	0.548	1.008 (2,139)	0.368	0.014	1.677 (3.218,223.649)	0.169	0.024
Untreated hotspot anger	37.973 (2.404,334.092)	<0.001	0.215	0.965 (2,139)	0.384	0.014	1.423 (4.807,334.092)	0.217	0.02
Untreated hotspot arousal	19.068 (2.105,292.546)	<0.001	0.121	0.245 (2,139)	0.783	0.004	0.802 (4.209,292.546)	0.530	0.011
Untreated hotspot disgust	16.912 (2.172,301.856)	<0.001	0.108	2.409 (2,139)	0.094	0.034	1.489 (4.343,301.856)	0.201	0.021
Untreated hotspot fear	65.302 (2.093,29.974)	<0.001	0.32	0.169 (2,139)	0.845	0.002	0.556 (4.187,29.974)	0.703	0.008
Untreated hotspot guilt	85.918 (2.043,283.933)	<0.001	0.382	2.379 (2,139)	0.096	0.033	0.392 (4.085,283.933)	0.818	0.006
Untreated hotspot sadness	109.211 (2.258,313.804)	<0.001	0.44	0.596 (2,139)	0.552	0.009	0.277 (4.515,313.804)	0.911	0.004

#### Screening measures

To determine eligibility for participation, a comprehensive screening battery was used:

- Mini-International Neuropsychiatric Interview (M. I. N. I. 5.0.0; Sheehan et al., 1998; Polish translation: Masiak and Przychoda, 1998) – Axis I disorders,
- Structured Clinical Interview for DSM-5 Personality Disorders (SCID-5-PD; APA, 2013; Polish translation: Popiel et al., 2019),
- Brief symptom checklists for anxiety, PTSD, OCD, panic, and agoraphobia (APA, 2013; back-translated version),
- Alcohol Use Disorders Identification Test (AUDIT) and Drug Abuse Screening Test (DAST-10).

### Data analysis

All analyses were conducted following the *intention-to-treat* (ITT) principle using a *last observation carried forward* (LOCF) approach for missing data imputation (analysed sample consisted of 145 participants). To verify the robustness of the findings, additional *per-protocol* (PP) sensitivity analyses were conducted and are reported in the Supplementary material (sample differs depending on modality of measure).

To assess treatment short- and long-term efficacy and stability (H1, H2), we conducted a series of 3 (Group: ImRs, ImRs-DSR, IE) × 4 (Time: Pre-treatment, Post-treatment, 3-month follow-up, 6-month follow-up) mixed ANOVAs with repeated measures on the Time factor. These analyses were conducted separately for each outcome variable and each experimental context (recall, renewal, reinstatement), with focus on both subjective and physiological measures (self-reported arousal and emotions, skin conductance level [SCL], and salivary alpha-amylase [sAA]). All time points were analysed simultaneously, using Bonferoni correction at post-hoc, to control multiple comparisons. For each outcome, effect sizes (η^2^_p_) are reported. Where relevant, Greenhouse–Geisser corrections were applied.

Importantly, physiological reactivity was analysed separately for the anticipation phase (the first three segments of the audio script), which was present in all procedures (recall, renewal, reinstatement), and the hotspot phase (the emotionally intense segment), which was included only in the reinstatement procedure and only for SCL recordings. As labelling emotions during the imagery task could distort the emotional experience (see Torre and Lieberman, 2018), subjective emotional ratings were collected exclusively at the end of each procedure (i.e., post-treatment, 3-month and 6-month follow-ups), and therefore reflect retrospective evaluations of emotional experience during scenario imagery.

To test our specific hypotheses regarding treatment effects over time, we additionally performed planned contrasts focused on interaction effects. Specifically, we compared change from pre- to post-treatment, pre- to 3-month follow-up, and pre- to 6-month follow-up between selected pairs of groups:

- ImRs > IE, Pre > Post, to test the therapeutic superiority of rescripting over exposure
- ImRs-DSR > ImRs, Pre > Post, to test the added value of reconsolidation disruption

The generalisation and stability of treatment effects (H3) was examined by comparing physiological and subjective responses to *untreated past criticism scenarios* and *future criticism scenarios*, presented in contexts different from the treatment setting (i.e., renewal and reinstatement procedures). To obtain pre-treatment SCL and rating values for the future scenario, we averaged the data from criticism scenarios at pre-treatment (both treated and untreated). These analyses followed the same 3 groups × 4 time points mixed ANOVA structure, with planned contrasts testing for between-group differences in generalisation.

Mediation analyses examined prediction error (PE) as a potential mechanism of change (H4). PE was operationalised as a physiological arousal shift (increase in SCL) immediately following the expected hotspot (replaced by rescripting) during the first intervention session. We used binary Group factor- both rescripting conditions collapsed into one, and IE as a second. We also operationalised Therapy Efficacy as a difference between pre- versus post-treatment in Skin Conductance Level during hotspot phase. Our model tested whether binary Group predicted Therapy Efficacy, and whether this relationship is mediated by PE.

All statistical analyses were conducted using JASP (v0.19). The significance threshold was set at *α* = 0.05. Sensitivity analyses using the per-protocol approach are reported in the Supplementary materials.

## Results

### Treated criticism scenario—physiological response (ITT analysis, summary)

Physiological responses to the treated criticism scenario were examined using repeated-measures ANOVAs across four time points and three intervention groups (IE, ImRs, ImRs-DSR),separately for recall, renewal and reinstatement.

During the anticipation phase, significant main effects of time were observed across all three experimental procedures – recall (*F* = 57.75, *p* < 0.001, η^2^_p_ = 0.30), renewal (*F* = 108.08, *p* < 0.001, η^2^_p_ = 0.45), and reinstatement (*F* = 98.90, *p* < 0.001, η^2^_p_ = 0.42) – indicating substantial and consistent reductions in anticipatory arousal following treatment. Time × Group interactions were non-significant or only marginally approached significance, suggesting comparable improvement across groups, although contrast analyses revealed some differences in SCL reduction over time between groups (see Contrast analysis section).

In the hotspot phase, reflecting peak emotional reactivity, SCL also decreased significantly over time, *F*(2.60, 342.52) = 18.16, *p* < 0.001, η^2^_p_ = 0.119. Although the Time x Group interaction was not statistically significant (*p* = 0.135), descriptive data suggested slightly greater reductions in the ImRs group, while the IE group showed a modest rebound at follow-up.

Overall, the ITT analyses demonstrated robust and sustained reductions in physiological arousal across time and procedures, with comparable benefits observed across all three intervention types.

**Figure 4:**
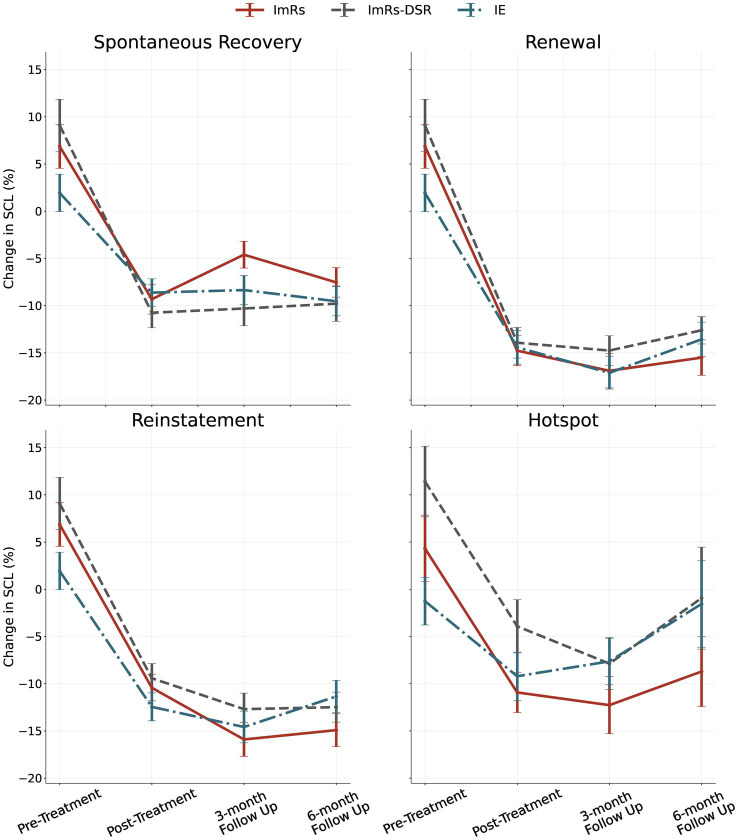
SCL results during anticipation phases of Recall, Renewal, Reinstatement and during Hotspot at Reinstatement for each group at different follow ups (bars represent 95% CI).

Levels of salivary alpha-amylase (sAA) did not show consistent evidence of treatment-related change. A main effect of time was not significant, *F*(2.67, 338.70) = 1.02, *p* = 0.378, η^2^_p_ = 0.008, and no group or time × group interaction effects emerged (all *p*s > 0.23). This indicates that the intervention procedures had no measurable impact on physiological stress reactivity as indexed by sAA across the study period (see Figure 4 for SCL results and Tables 3, 4).

**Table 4:** ITT descriptive intention-to-treatment descriptive analysis.

Analysis	tp1			tp6			tp7			tp8		
	IE	ImRs	ImRs-DSR	IE	ImRs	ImRs-DSR	IE	ImRs	ImRs-DSR	IE	ImRs	ImRs-DSR
SCL anticipation treated criticism recall	1.932 (13.482)	6.866 (16.444)	9.094 (17.826)	−8.735 (9.892)	−9.444 (10.805)	−11.232 (10.604)	−8.855 (9.815)	−4.900 (9.511)	−10.500 (12.211)	−11.231 (10.401)	−10.021 (11.742)	−9.316 (10.988)
SCL anticipation treated criticism reinstatement	1.932 (13.482)	6.866 (16.444)	9.094 (17.826)	−13.102 (10.461)	−11.514 (9.911)	−10.136 (10.410)	−14.938 (9.651)	−15.753 (12.001)	−13.252 (10.787)	−12.997 (10.895)	−15.072 (11.871)	−11.897 (8.995)
SCL anticipation treated criticism renewal	1.932 (13.482)	6.866 (16.444)	9.094 (17.826)	−14.377 (12.043)	−14.437 (10.976)	−13.589 (10.799)	−16.393 (10.555)	−16.063 (12.201)	−14.955 (9.950)	−14.617 (11.359)	−16.558 (12.294)	−12.119 (8.419)
SCL hotspot treated criticism reinstatement	−1.269 (17.063)	4.313 (24.876)	11.409 (24.246)	−9.159 (18.481)	−13.355 (15.902)	−6.914 (19.828)	−9.100 (15.701)	−13.104 (17.190)	−8.684 (16.084)	−3.457 (27.945)	−13.176 (20.579)	−5.251 (29.474)
SCL anticipation future criticism	−1.101 (6.986)	0.690 (10.986)	2.804 (11.755)	7.511 (4.848)	7.783 (5.472)	8.595 (5.153)	6.829 (4.785)	5.764 (4.899)	7.217 (5.072)	5.803 (4.270)	6.020 (4.525)	7.356 (4.655)
SCL anticipation untreated criticism recall	−5.664 (11.090)	−7.404 (12.488)	−6.620 (7.580)	−1.010 (12.280)	−3.335 (10.080)	−3.143 (10.318)	−8.529 (10.678)	−5.936 (9.482)	−4.492 (9.724)	−6.157 (15.838)	−4.560 (14.467)	−3.719 (12.210)
SCL anticipation untreated criticism reinstatement	−5.664 (11.090)	−7.404 (12.488)	−6.620 (7.580)	−7.882 (9.519)	−7.591 (8.727)	−5.806 (9.064)	−9.198 (10.633)	−6.172 (11.120)	−5.731 (9.931)	−10.166 (10.514)	−8.440 (10.862)	−6.408 (9.123)
SCL anticipation untreated criticism renewal	−5.664 (11.090)	−7.404 (12.488)	−6.620 (7.580)	−9.121 (9.314)	−9.150 (10.461)	−5.278 (9.933)	−9.399 (12.451)	−9.785 (13.052)	−5.827 (11.759)	−9.576 (11.708)	−7.507 (10.417)	−4.561 (12.018)
SCL anticipation future criticism	−1.101 (6.986)	0.690 (10.986)	2.804 (11.755)	7.511 (4.848)	7.783 (5.472)	8.595 (5.153)	6.829 (4.785)	5.764 (4.899)	7.217 (5.072)	5.803 (4.270)	6.020 (4.525)	7.356 (4.655)
SCL hotspot future criticism	−4.818 (10.484)	−3.037 (16.326)	1.350 (13.333)	7.417 (4.516)	7.608 (5.129)	9.112 (5.086)	6.536 (4.480)	5.941 (4.976)	7.215 (5.025)	5.865 (4.231)	5.980 (4.420)	7.329 (4.565)
Rating of anticipation phase treated criticism arousal	4.490 (1.835)	4.850 (1.923)	4.284 (1.984)	3.271 (1.710)	3.640 (1.787)	3.489 (1.900)	3.063 (1.841)	3.280 (1.896)	3.261 (1.927)	3.115 (1.877)	3.430 (1.974)	3.307 (1.989)
Rating of anticipation phase treated criticism disgust	2.146 (1.574)	2.540 (1.937)	2.523 (1.923)	1.344 (0.716)	1.310 (0.782)	1.784 (1.480)	1.479 (0.805)	1.580 (1.239)	1.875 (1.343)	1.510 (1.044)	1.770 (1.679)	1.739 (1.251)
Rating of anticipation phase treated criticism fear	3.729 (1.822)	4.170 (2.212)	3.875 (2.038)	2.104 (1.225)	2.130 (1.151)	2.455 (1.524)	1.896 (1.180)	1.980 (1.160)	2.477 (1.772)	1.906 (1.266)	2.200 (1.669)	2.398 (1.797)
Rating of anticipation phase treated criticism FOC	6.625 (1.468)	7.100 (1.317)	6.886 (1.156)	6.667 (1.674)	6.030 (1.931)	6.250 (1.999)	6.010 (1.867)	5.510 (2.069)	5.795 (1.960)	5.979 (1.984)	5.560 (2.152)	5.455 (2.347)
Rating of anticipation phase treated criticism guilt	3.792 (2.324)	4.290 (2.699)	3.693 (1.905)	1.635 (0.892)	1.940 (0.978)	2.409 (1.395)	1.906 (1.119)	2.200 (1.374)	2.432 (1.535)	1.813 (1.040)	2.150 (1.575)	2.330 (1.529)
Rating of anticipation phase treated criticism IMM	6.698 (1.620)	7.000 (1.351)	6.886 (1.151)	7.010 (1.655)	6.460 (1.720)	6.511 (1.978)	6.458 (1.747)	5.910 (2.087)	6.170 (2.051)	6.240 (1.919)	5.810 (2.215)	5.977 (2.295)
Rating of anticipation phase treated criticism sadness	3.833 (1.790)	4.300 (2.378)	3.534 (1.515)	1.906 (1.055)	2.030 (1.012)	2.500 (1.479)	1.969 (1.205)	1.920 (0.986)	2.273 (1.264)	1.948 (1.107)	2.170 (1.288)	2.125 (1.206)
Rating of anticipation phase treated criticism anger	2.708 (1.688)	2.870 (1.681)	2.614 (1.667)	1.448 (0.753)	1.530 (0.752)	1.784 (1.374)	1.438 (0.719)	1.810 (1.328)	1.739 (1.009)	1.542 (1.224)	1.870 (1.622)	1.705 (0.904)
Rating of anticipation phase future criticism anger	2.739 (1.351)	2.707 (1.220)	2.744 (1.423)	1.543 (0.900)	1.734 (1.010)	1.526 (0.924)	1.446 (0.717)	1.915 (1.431)	1.872 (1.613)	1.413 (0.702)	1.862 (1.087)	1.590 (0.993)
Rating of anticipation phase future criticism arousal	4.592 (1.605)	4.585 (1.643)	4.199 (1.715)	4.424 (1.963)	4.564 (1.935)	4.385 (1.801)	3.598 (2.056)	4.138 (1.820)	3.641 (2.173)	3.522 (1.903)	3.989 (1.804)	3.705 (2.095)
Rating of anticipation phase future criticism disgust	2.033 (1.260)	2.590 (1.616)	2.635 (1.654)	1.370 (0.627)	1.809 (1.509)	1.679 (1.259)	1.337 (0.700)	2.011 (1.699)	1.718 (1.281)	1.326 (0.634)	1.947 (1.537)	1.769 (1.063)
Rating of anticipation phase future criticism fear	3.908 (1.705)	4.059 (1.756)	3.910 (1.732)	3.272 (1.905)	3.543 (2.103)	3.077 (1.979)	2.783 (1.772)	3.160 (2.038)	2.936 (1.759)	2.620 (1.799)	3.053 (1.926)	2.833 (1.615)
Rating of anticipation phase future criticism guilt	3.826 (1.733)	4.027 (1.817)	4.071 (1.515)	2.435 (1.672)	2.894 (1.936)	2.077 (1.485)	2.337 (1.626)	2.819 (1.831)	2.154 (1.582)	1.989 (1.327)	2.638 (1.744)	2.333 (1.515)
Rating of anticipation phase future criticism sadness	3.957 (1.385)	3.957 (1.572)	3.840 (1.485)	1.989 (1.108)	2.553 (1.623)	1.859 (1.262)	1.957 (1.277)	2.574 (1.827)	2.051 (1.642)	1.891 (1.229)	2.426 (1.460)	2.000 (1.318)
Rating of anticipation phase untreated criticism anger	2.724 (1.344)	2.750 (1.245)	2.636 (1.392)	1.427 (0.686)	1.555 (0.678)	1.807 (1.430)	1.422 (0.675)	1.790 (0.960)	1.801 (1.039)	1.500 (1.116)	1.800 (1.030)	1.642 (0.808)
Rating of anticipation phase untreated criticism arousal	4.604 (1.607)	4.705 (1.681)	4.136 (1.757)	3.641 (1.604)	3.805 (1.722)	3.597 (1.829)	3.156 (1.725)	3.270 (1.792)	3.114 (1.718)	3.120 (1.804)	3.410 (1.771)	3.182 (1.829)
Rating of anticipation phase untreated criticism disgust	2.021 (1.244)	2.520 (1.598)	2.551 (1.603)	1.318 (0.633)	1.350 (0.720)	1.710 (1.429)	1.411 (0.689)	1.625 (0.947)	1.790 (1.229)	1.401 (0.716)	1.685 (1.196)	1.665 (0.937)
Rating of anticipation phase untreated criticism fear	3.849 (1.699)	4.085 (1.737)	3.858 (1.664)	2.172 (1.058)	2.350 (1.101)	2.335 (1.328)	2.010 (1.059)	2.135 (1.212)	2.313 (1.373)	1.906 (0.994)	2.195 (1.464)	2.267 (1.393)
Rating of anticipation phase untreated criticism guilt	3.781 (1.716)	4.095 (1.911)	3.955 (1.511)	1.771 (0.863)	2.115 (1.009)	2.290 (1.304)	1.922 (1.011)	2.210 (1.316)	2.216 (1.279)	1.818 (0.858)	2.100 (1.372)	2.278 (1.358)
Rating of anticipation phase untreated criticism sadness	3.901 (1.410)	4.025 (1.599)	3.727 (1.446)	1.922 (0.845)	2.180 (0.983)	2.426 (1.298)	1.922 (0.975)	2.145 (0.992)	2.176 (1.142)	1.839 (0.824)	2.235 (1.093)	2.102 (1.178)
Rating of hotspot phase treated criticism anger	4.063 (2.462)	4.980 (2.810)	4.091 (2.380)	2.646 (1.918)	3.020 (1.964)	2.795 (1.995)	2.667 (1.982)	3.220 (2.324)	3.091 (2.044)	2.729 (2.322)	2.740 (1.882)	2.977 (2.226)
Rating of hotspot phase treated criticism arousal	5.333 (2.244)	6.180 (2.106)	4.636 (2.677)	4.271 (2.050)	4.720 (2.241)	4.614 (2.345)	4.083 (2.277)	4.440 (2.476)	4.114 (2.470)	3.979 (2.302)	4.460 (2.435)	4.068 (2.500)
Rating of hotspot phase treated criticism disgust	3.063 (2.338)	3.640 (2.827)	4.000 (2.795)	1.875 (1.734)	1.840 (1.419)	2.591 (2.149)	2.271 (2.008)	2.280 (2.071)	2.705 (2.226)	2.146 (1.774)	2.080 (1.988)	2.455 (2.017)
Rating of hotspot phase treated criticism fear	5.188 (2.367)	5.320 (2.759)	5.136 (2.557)	3.167 (2.272)	3.080 (2.098)	3.455 (2.118)	3.271 (2.359)	3.000 (2.449)	3.227 (2.371)	2.958 (2.324)	2.660 (2.096)	3.227 (2.271)
Rating of hotspot phase treated criticism guilt	5.917 (2.616)	6.180 (2.716)	6.205 (2.288)	3.292 (2.163)	3.280 (2.080)	4.091 (2.291)	3.708 (2.423)	3.700 (2.682)	4.068 (2.618)	3.500 (2.388)	3.300 (2.501)	3.886 (2.335)
Rating of hotspot phase treated criticism sadness	6.063 (2.177)	6.320 (2.386)	6.432 (1.910)	3.771 (2.076)	3.560 (1.831)	3.955 (2.382)	3.833 (2.504)	3.640 (2.248)	4.068 (2.225)	3.604 (2.439)	3.360 (2.136)	3.705 (2.278)
Rating of hotspot phase future criticism anger	4.261 (2.203)	4.926 (2.226)	4.167 (1.941)	3.348 (2.253)	3.936 (2.230)	4.308 (2.319)	3.239 (2.330)	3.596 (2.300)	3.846 (2.323)	3.130 (2.166)	3.553 (2.114)	3.308 (2.041)
Rating of hotspot phase future criticism arousal	5.457 (1.840)	5.819 (1.909)	4.872 (2.430)	5.500 (2.229)	5.638 (2.048)	5.487 (2.050)	4.652 (2.273)	4.936 (2.068)	4.897 (2.583)	4.543 (2.168)	4.809 (2.007)	4.795 (2.577)
Rating of hotspot phase future criticism disgust	2.783 (1.876)	3.691 (2.566)	4.013 (2.553)	2.261 (1.769)	3.064 (2.363)	2.974 (2.121)	2.196 (1.572)	2.809 (1.930)	2.974 (2.345)	2.304 (1.645)	3.000 (2.303)	2.846 (2.277)
Rating of hotspot phase future criticism fear	5.261 (2.076)	5.213 (2.166)	5.167 (2.374)	4.087 (2.374)	4.319 (2.314)	3.359 (2.378)	3.435 (2.218)	3.617 (2.299)	3.692 (2.525)	3.239 (2.272)	3.660 (2.362)	3.590 (2.702)
Rating of hotspot phase future criticism guilt	6.011 (2.112)	6.138 (2.317)	6.436 (1.957)	4.609 (2.745)	5.128 (2.281)	4.615 (2.662)	4.130 (2.680)	4.383 (2.410)	4.615 (2.642)	3.826 (2.719)	4.149 (2.587)	4.462 (2.594)
Rating of hotspot phase future criticism sadness	6.304 (1.638)	6.340 (2.124)	6.692 (1.680)	4.500 (2.373)	4.915 (2.205)	4.462 (2.360)	3.935 (2.361)	4.362 (2.540)	4.051 (2.449)	3.826 (2.407)	4.340 (2.434)	3.846 (2.651)
Rating of hotspot phase future criticism anger	4.458 (2.518)	5.120 (2.370)	4.068 (2.172)	2.833 (1.971)	3.200 (1.761)	3.182 (2.160)	2.917 (1.998)	3.120 (1.945)	3.159 (2.134)	2.708 (2.133)	3.260 (2.058)	2.841 (2.112)
Rating of hotspot phase future criticism arousal	5.521 (1.989)	5.720 (2.138)	5.091 (2.541)	4.521 (2.183)	4.960 (2.407)	4.886 (2.274)	4.458 (2.240)	4.460 (2.451)	4.386 (2.563)	4.188 (2.275)	4.460 (2.332)	4.182 (2.789)
Rating of hotspot phase future criticism disgust	2.604 (2.181)	3.740 (2.679)	4.068 (2.823)	1.979 (1.523)	2.300 (1.821)	2.773 (2.301)	2.333 (1.950)	2.620 (2.194)	2.818 (2.326)	2.208 (1.924)	2.580 (2.269)	2.682 (2.133)
Rating of hotspot phase future criticism fear	5.125 (2.438)	5.280 (2.391)	5.295 (2.681)	3.500 (2.297)	3.780 (2.427)	3.568 (2.491)	3.250 (2.302)	3.080 (2.266)	3.545 (2.565)	2.771 (2.224)	2.960 (2.303)	3.227 (2.560)
Rating of hotspot phase future criticism guilt	5.958 (2.405)	6.160 (2.444)	6.705 (2.319)	3.792 (2.250)	4.080 (2.538)	4.682 (2.494)	3.708 (2.518)	3.520 (2.620)	4.568 (2.636)	3.375 (2.358)	3.380 (2.364)	4.318 (2.595)
Rating of hotspot phase future criticism sadness	6.417 (1.944)	6.560 (2.082)	6.841 (1.879)	4.146 (2.212)	4.440 (2.251)	4.727 (2.224)	3.917 (2.431)	3.820 (2.439)	4.295 (2.184)	3.792 (2.333)	4.020 (2.317)	4.091 (2.504)
Beck depression inventory	14.936 (6.370)	13.380 (5.646)	15.000 (6.094)	12.723 (8.200)	12.860 (6.007)	11.465 (6.957)	12.191 (7.655)	12.140 (5.990)	13.047 (7.518)	9.340 (7.530)	10.200 (6.518)	11.651 (9.240)
Frost multidimensional perfectionism scale	3.062 (0.637)	3.251 (0.578)	3.128 (0.617)	2.965 (0.649)	3.126 (0.616)	3.135 (0.596)	3.088 (0.610)	3.144 (0.608)	3.130 (0.590)	3.062 (0.701)	3.099 (0.651)	3.082 (0.647)
Leibowitz social anxiety scale	2.073 (0.533)	2.059 (0.456)	2.008 (0.493)	2.072 (0.496)	2.037 (0.447)	2.010 (0.513)	2.036 (0.535)	2.007 (0.461)	1.974 (0.555)	1.984 (0.561)	2.026 (0.490)	1.935 (0.546)
Performance failure appraisal inventory (fear of failure scale)	120.149 (9.184)	120.680 (9.155)	118.744 (11.320)	111.723 (21.251)	115.880 (18.721)	111.535 (22.183)	112.149 (23.605)	115.760 (20.346)	113.442 (16.836)	108.638 (27.182)	111.760 (22.091)	106.279 (23.054)
Pure procrastination scale	2.877 (1.051)	2.978 (0.955)	2.709 (0.952)	2.783 (1.025)	3.067 (0.996)	2.782 (0.848)	2.857 (1.026)	2.926 (0.987)	2.742 (0.841)	2.830 (1.082)	2.892 (1.012)	2.820 (0.933)
Salivary alpha amylase	140.527 (91.230)	111.418 (76.090)	139.882 (89.356)	138.194 (91.869)	101.254 (70.448)	126.414 (90.184)						
PTSD DSM-V scale	0.412 (0.588)	0.507 (0.868)	0.447 (0.699)							0.343 (0.687)	0.288 (0.621)	0.310 (0.609)
General anxiety disorder	1.945 (0.538)	1.898 (0.540)	1.881 (0.566)							1.689 (0.556)	1.744 (0.518)	1.758 (0.471)
Panic disorder DSM-V scale	1.283 (0.376)	1.280 (0.421)	1.323 (0.514)							1.213 (0.416)	1.170 (0.343)	1.233 (0.343)
Yale-Brown obsessive-compulsive scale	13.170 (5.787)	12.280 (6.111)	10.930 (5.663)							10.957 (6.963)	11.500 (5.733)	10.884 (6.695)

### Subjective response during treated criticism scenario (ITT analysis)

**Figure 5:**
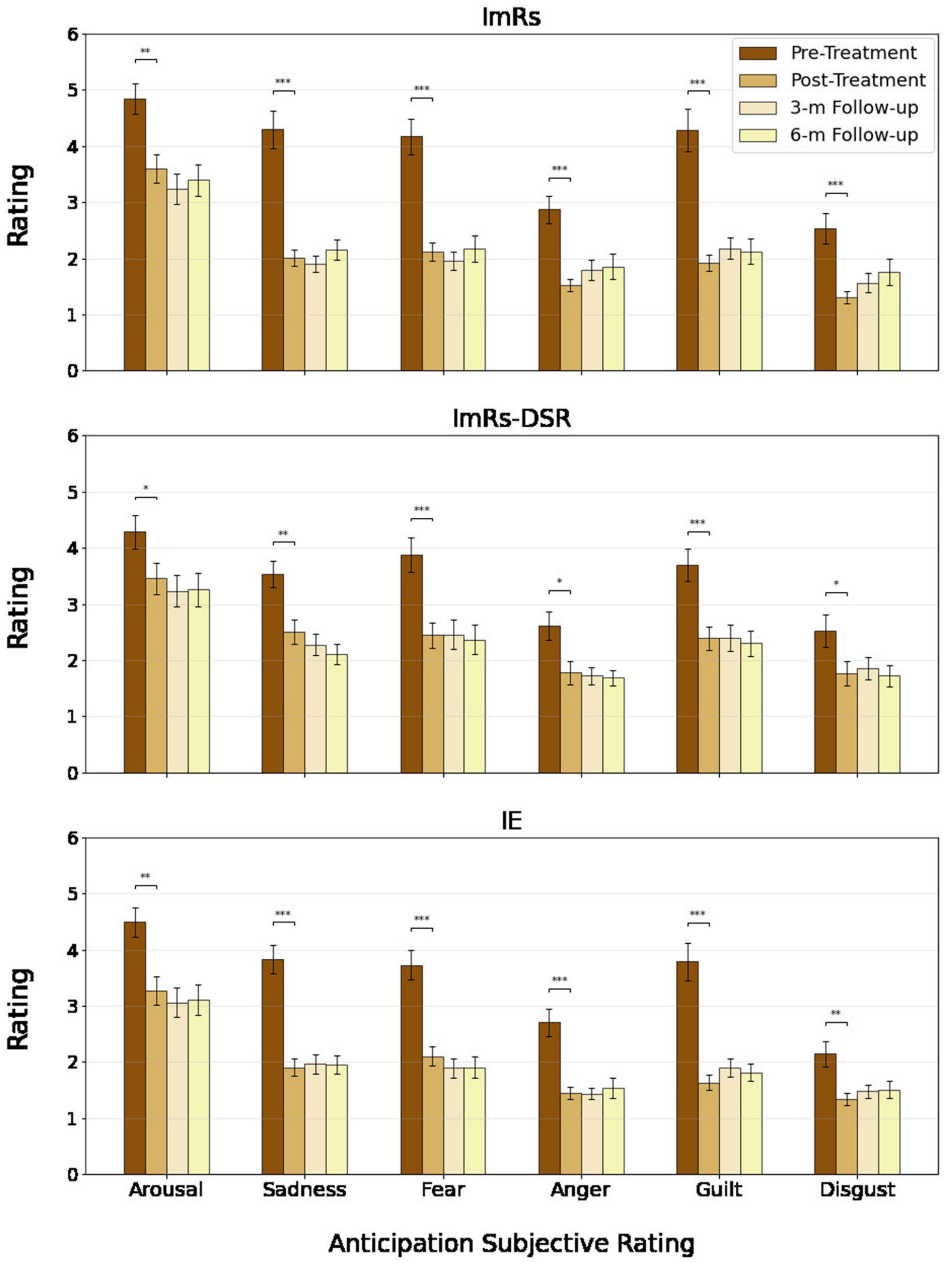
Rating of anticipation phase of treated criticism across groups and time points.

**Figure 6:**
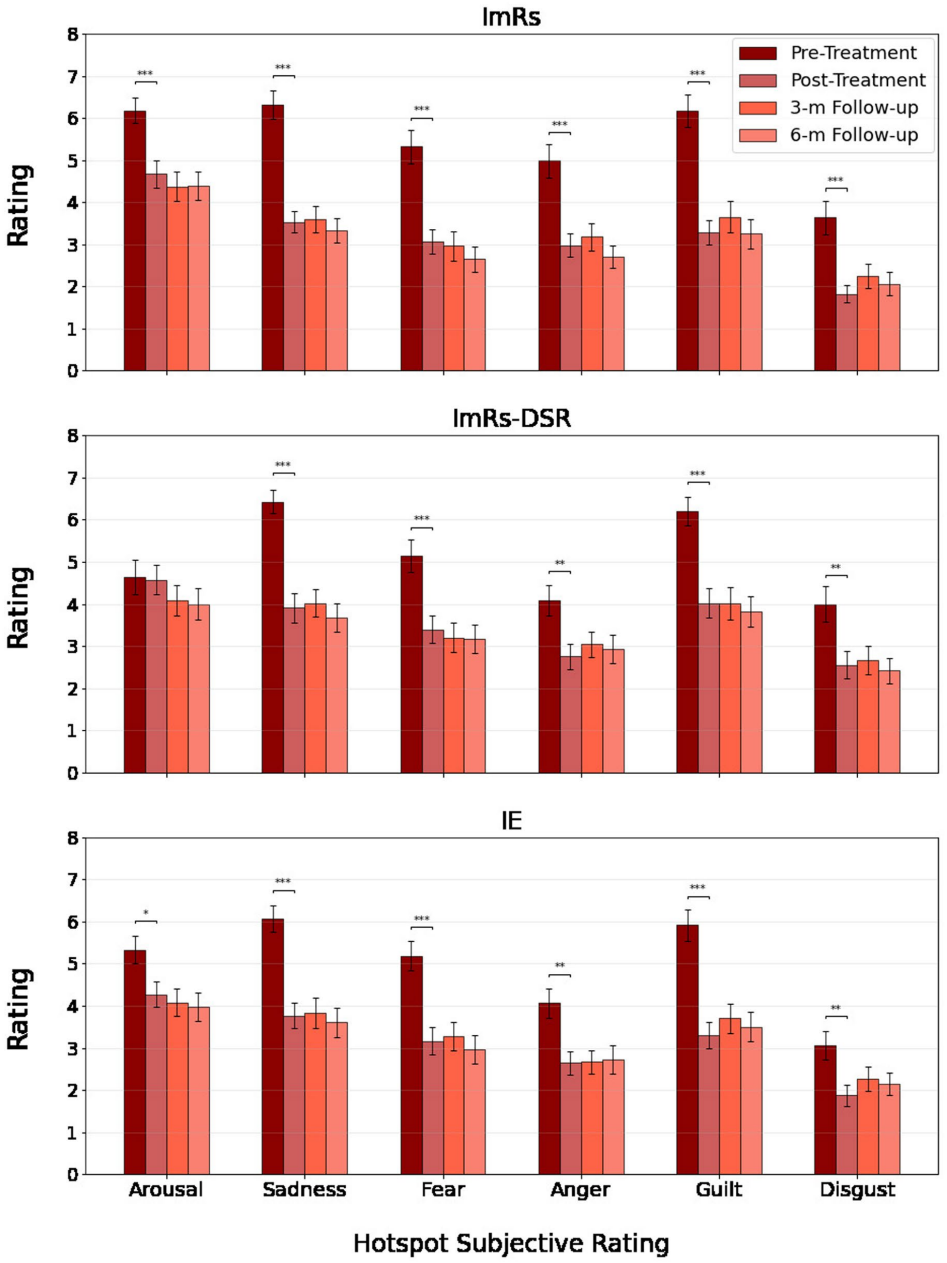
Hotspot rating of treated criticism across groups and time points.

Across both anticipation and hotspot phases, repeated-measures ANOVAs revealed robust main effects of time for all assessed negative emotions, indicating significant and sustained reductions in subjective distress from pre-treatment to post-treatment and across the 3- and 6-month follow-ups (see Figures 5, 6). Effect sizes for time effects ranged from moderate to very large (η^2^_p_ = 0.12–0.44 in anticipation; η^2^_p_ = 0.12–0.44 in hotspot). The only significant time × group interaction was observed for sadness during anticipation (η^2^_p_ = 0.047), reflecting somewhat greater post-treatment decreases in the ImRs and IE groups compared to ImRs-DSR. No other main effects of group or interactions were significant (all *p*s > 0.07), although contrast analysis revealed interaction for arousal rating (see Contrast analysis section and Figure 6.). Overall, results demonstrate that all three interventions led to durable decreases in subjective negative affect during anticipation and peak emotional reactivity, with improvements remaining stable through follow-up.

### Fear of failure results (ITT analysis)

Analyses of the fear of failure (PFAI) did not reveal significant group or time × group interaction effects (all ps > 0.14). A main effect of time was observed, *F*(2.55, 354.12) = 6.48, *p* < 0.001, η^2^_p_ = 0.045, reflecting a small overall reduction in PFAI scores across assessments. *Post hoc* comparisons indicated that reductions from pre-treatment to post-treatment (ΔM = −3.21, *p* = 0.009, *d* = 0.28) and to the 3-month follow-up (tp7; ΔM = −3.85, *p* = 0.006) were significant, but changes did not remain robust at the 6-month follow-up (tp8, *p* = 0.087). These results suggest modest improvements in fear of failure following intervention, but without clear differentiation between conditions.

### Generalizability and stability SCL—untreated and future criticism (ITT analysis)

For the untreated criticism scenario, physiological responses showed no consistent time effects across anticipation, renewal, or reinstatement phases. Only a weak reduction emerged in the recall procedure (η^2^_p_ ≈ 0.03), with no lasting changes at follow-ups. No significant group or interaction effects were observed, indicating stable arousal across interventions.

In contrast, future criticism elicited robust increases in physiological arousal over time. Significant main effects of time were found for both anticipation and hotspot phases, with small to medium effect sizes (η^2^_p_ ≈ 0.20–0.30). SCL rose markedly from pre-treatment to post-treatment and remained elevated across follow-ups, with no differences between groups.

Overall, these findings suggest that interventions did not generalise to untreated autobiographical criticism, consistent across all conditions. They were also associated with heightened reactivity to imagined future criticism.

### Generalizability and stability—subjective measures (ITT analysis)

In both the future and untreated criticism scenarios, participants reported significant changes in emotional reactivity during anticipation phase over time (*p* < 0.001 in all main effects of time), with no significant main effects of condition (all *p*s > 0.20) or Time × Group interactions (all *p*s > 0.13). This indicates comparable improvement patterns across intervention groups.

For the future criticism scenario (anticipation and hotspot phase), large time effects were found for sadness (η^2^_p_ ≈ 0.41–44), fear (η^2^_p_l ≈ 0.32–34), and guilt (η^2^_p_ ≈ 0.23–24), all showing robust decreases from pre-treatment to post-treatment that remained stable through follow-ups. Anger, arousal, and disgust also declined significantly over time, though with smaller effect sizes (η^2^_p_ ≈ 0.05–0.09).

For the untreated criticism scenario, significant reductions were observed across both anticipation and hotspot phases for most negative emotions. The largest effects were seen for sadness (η^2^_p_ ≈ 0.41–0.44), fear (η^2^_p_ ≈ 0.34–0.45), and guilt (η^2^_p_ ≈ 0.22–0.49), with moderate declines in anger, arousal, and disgust (η^2^_p_ ≈ 0.09–0.30). Across both scenarios, the absence of significant group differences and interactions suggests that reductions in subjective emotional reactivity were non-specific to treatment type. Worth noting, this finding is contradictory to psychophysiological results, although may be the result of recency effect (see Discussion).

### Contrast analysis (ITT and PP analysis)

Although the majority of exploratory contrasts did not reach statistical significance, several noteworthy effects emerged. First, a rebound effect was observed in the IE group compared to ImRs at the 6-month follow-up, reflected in increased SCL responses to the hotspot during reinstatement [*t*(135) = −2.231, *p* = 0.027] and, at the trend level, to the anticipation phase during renewal [*t*(135) = −1.814, *p* = 0.072] and reinstatement [*t*(135) = −1.774, *p* = 0.078]. In addition, analyses demonstrated the superiority of ImRs over ImRs-DSR in reducing subjective ratings of guilt and sadness for the anticipation phase at post treatment [guilt: t(139) = −2.344, *p* = 0.021; sadness: t(139) = −3.192, *p* = 0.002]. A consistent pattern further indicated stronger reductions in the arousal ratings of the hotspot phase in the ImRs group relative to ImRs-DSR, both immediately post-treatment and across follow-up sessions [post-treatment: t(139) = −2.665, *p* = 0.009; 3-month follow-up: t(139) = −2.227, *p* = 0.028; 6-month follow-up: t(139) = −2.033, *p* = 0.044; see Figure 6].

Sensitivity analyses using the per-protocol dataset largely confirmed these findings. The rebound effect in IE compared to ImRs was again present at the 6-month follow-up, although here it emerged to be significant during the anticipation phase at reinstatement [*t*(79) = −2.305, *p* = 0.024], but not during hotspot [*t*(76) = −1.279, *p* = 0.205]. Moreover, the superiority of ImRs over ImRs-DSR in reducing arousal ratings was also observed for these analyses at pre-treatment and at the 3-month follow-up [pre-treatment: *t*(87) = −2.139, *p* = 0.035; 3-month follow-up: *t*(87) = −2.063, *p* = 0.042] (Table 5).

**Table 5:** ITT contrast analysis.

Analysis	ImRs>IE Pre>Post-Treatment		ImRs>IE Pre-Treatment>3-m F-up		ImRs>IE Pre-Treatment>6-m F-up		ImRs-DSR>ImRs Pre>Post-Treatment		ImRs-DSR>ImRs Pre-Treatment>3-m F-up		ImRs-DSR>ImRs Pre-Treatment>6-m F-up	
	*t* (df)	*p*	*t* (df)	*p*	*t* (df)	*p*	*t* (df)	*p*	*t* (df)	*p*	*t* (df)	*p*
SCL anticipation treated criticism recall	−1.495 (df = 135)	0.137	−0.244 (df = 135)	0.808	−0.970 (df = 135)	0.334	1.039 (df = 135)	0.301	1.902 (df = 135)	0.059	0.387 (df = 135)	0.699
SCL anticipation treated criticism reinstatement	−0.903 (df = 135)	0.368	−1.388 (df = 135)	0.168	−1.774 (df = 135)	0.078	0.224 (df = 135)	0.823	−0.064 (df = 135)	0.949	−0.234 (df = 135)	0.815
SCL anticipation treated criticism renewal	−1.238 (df = 135)	0.218	−1.130 (df = 135)	0.26	−1.814 (df = 135)	0.072	0.334 (df = 135)	0.739	0.269 (df = 135)	0.789	−0.570 (df = 135)	0.57
SCL hotspot treated criticism reinstatement	−1.741 (df = 135)	0.084	−1.651 (df = 135)	0.101	−2.231 (df = 135)	0.027	0.114 (df = 135)	0.909	0.450 (df = 135)	0.654	−0.118 (df = 135)	0.906
SCL anticipation untreated criticism recall	−0.199 (df = 134)	0.843	1.418 (df = 134)	0.159	0.974 (df = 134)	0.332	0.194 (df = 134)	0.846	−0.208 (df = 134)	0.836	−0.016 (df = 134)	0.987
SCL anticipation untreated criticism reinstatement	0.687 (df = 134)	0.493	1.563 (df = 134)	0.12	1.245 (df = 134)	0.215	−0.326 (df = 134)	0.745	0.108 (df = 134)	0.914	−0.433 (df = 134)	0.666
SCL anticipation untreated criticism renewal	0.561 (df = 134)	0.576	0.420 (df = 134)	0.675	1.215 (df = 134)	0.227	−0.977 (df = 134)	0.33	−0.949 (df = 134)	0.344	−0.665 (df = 134)	0.507
SCL anticipation future criticism	−0.625 (df = 124)	0.533	−1.190 (df = 124)	0.236	−0.704 (df = 124)	0.483	0.520 (df = 124)	0.604	0.267 (df = 124)	0.79	0.337 (df = 124)	0.737
SCL hotspot future criticism	−0.544 (df = 124)	0.588	−0.794 (df = 124)	0.428	−0.574 (df = 124)	0.567	0.956 (df = 124)	0.341	1.009 (df = 124)	0.315	1.014 (df = 124)	0.312
Salivary alpha amylase	−0.826 (df = 123)	0.411					0.330 (df = 123)	0.742				
Rating of anticipation phase treated criticism arousal	0.019 (df = 139)	0.985	−0.297 (df = 139)	0.767	−0.094 (df = 139)	0.925	−0.890 (df = 139)	0.375	−1.112 (df = 139)	0.268	−0.901 (df = 139)	0.369
Rating of anticipation phase treated criticism disgust	−1.274 (df = 139)	0.205	−0.857 (df = 139)	0.393	−0.382 (df = 139)	0.703	−1.430 (df = 139)	0.155	−0.892 (df = 139)	0.374	0.039 (df = 139)	0.969
Rating of anticipation phase treated criticism guilt	−0.436 (df = 139)	0.664	−0.441 (df = 139)	0.66	−0.336 (df = 139)	0.737	−2.344 (df = 139)	0.021	−1.744 (df = 139)	0.083	−1.586 (df = 139)	0.115
Rating of anticipation phase treated criticism sadness	−0.906 (df = 139)	0.367	−1.322 (df = 139)	0.188	−0.606 (df = 139)	0.545	−3.192 (df = 139)	0.002	−2.805 (df = 139)	0.006	−1.747 (df = 139)	0.083
Rating of anticipation phase treated criticism anger	−0.225 (df = 139)	0.822	0.617 (df = 139)	0.538	0.422 (df = 139)	0.673	−1.412 (df = 139)	0.16	−0.530 (df = 139)	0.597	−0.225 (df = 139)	0.822
Rating of anticipation phase future criticism anger	0.731 (df = 129)	0.466	1.408 (df = 129)	0.162	1.518 (df = 129)	0.131	0.770 (df = 129)	0.443	0.213 (df = 129)	0.831	0.932 (df = 129)	0.353
Rating of anticipation phase future criticism arousal	0.313 (df = 129)	0.755	1.060 (df = 129)	0.291	0.933 (df = 129)	0.353	−0.421 (df = 129)	0.674	0.205 (df = 129)	0.838	−0.192 (df = 129)	0.848
Rating of anticipation phase future criticism disgust	−0.350 (df = 129)	0.727	0.307 (df = 129)	0.759	0.177 (df = 129)	0.86	0.488 (df = 129)	0.626	0.855 (df = 129)	0.394	0.598 (df = 129)	0.551
Rating of anticipation phase future criticism fear	0.225 (df = 129)	0.822	0.447 (df = 129)	0.656	0.557 (df = 129)	0.578	0.571 (df = 129)	0.569	0.143 (df = 129)	0.887	0.135 (df = 129)	0.893
Rating of anticipation phase future criticism guilt	0.584 (df = 129)	0.561	0.668 (df = 129)	0.505	1.060 (df = 129)	0.291	1.861 (df = 129)	0.065	1.611 (df = 129)	0.11	0.790 (df = 129)	0.431
Rating of anticipation phase future criticism sadness	1.478 (df = 129)	0.142	1.499 (df = 129)	0.136	1.324 (df = 129)	0.188	1.449 (df = 129)	0.15	0.943 (df = 129)	0.347	0.732 (df = 129)	0.465
Rating of anticipation phase untreated criticism anger	0.348 (df = 139)	0.729	1.245 (df = 139)	0.215	0.936 (df = 139)	0.351	−1.219 (df = 139)	0.225	−0.444 (df = 139)	0.658	0.148 (df = 139)	0.882
Rating of anticipation phase untreated criticism arousal	0.159 (df = 139)	0.874	0.031 (df = 139)	0.975	0.445 (df = 139)	0.657	−0.879 (df = 139)	0.381	−0.974 (df = 139)	0.332	−0.782 (df = 139)	0.436
Rating of anticipation phase untreated criticism disgust	−1.769 (df = 139)	0.079	−1.082 (df = 139)	0.281	−0.750 (df = 139)	0.455	−1.219 (df = 139)	0.225	−0.495 (df = 139)	0.622	0.175 (df = 139)	0.861
Rating of anticipation phase untreated criticism fear	−0.177 (df = 139)	0.86	−0.318 (df = 139)	0.751	0.141 (df = 139)	0.888	−0.633 (df = 139)	0.528	−1.129 (df = 139)	0.261	−0.780 (df = 139)	0.437
Rating of anticipation phase untreated criticism guilt	0.089 (df = 139)	0.929	−0.071 (df = 139)	0.944	−0.086 (df = 139)	0.932	−0.904 (df = 139)	0.368	−0.394 (df = 139)	0.694	−0.853 (df = 139)	0.395
Rating of anticipation phase untreated criticism sadness	0.467 (df = 139)	0.641	0.331 (df = 139)	0.741	0.873 (df = 139)	0.384	−1.852 (df = 139)	0.066	−1.074 (df = 139)	0.285	−0.516 (df = 139)	0.606
Rating of hotspot phase treated criticism anger	−1.046 (df = 139)	0.297	−0.617 (df = 139)	0.538	−1.497 (df = 139)	0.137	−1.250 (df = 139)	0.213	−1.258 (df = 139)	0.21	−1.818 (df = 139)	0.071
Rating of hotspot phase treated criticism arousal	−0.754 (df = 139)	0.452	−0.917 (df = 139)	0.361	−0.661 (df = 139)	0.51	−2.665 (df = 139)	0.009	−2.227 (df = 139)	0.028	−2.033 (df = 139)	0.044
Rating of hotspot phase treated criticism disgust	−1.336 (df = 139)	0.184	−1.142 (df = 139)	0.255	−1.326 (df = 139)	0.187	−0.833 (df = 139)	0.406	−0.127 (df = 139)	0.899	−0.029 (df = 139)	0.977
Rating of hotspot phase treated criticism fear	−0.454 (df = 139)	0.651	−0.763 (df = 139)	0.447	−0.815 (df = 139)	0.416	−1.130 (df = 139)	0.26	−0.760 (df = 139)	0.449	−1.389 (df = 139)	0.167
Rating of hotspot phase treated criticism guilt	−0.540 (df = 139)	0.59	−0.472 (df = 139)	0.637	−0.823 (df = 139)	0.412	−1.510 (df = 139)	0.133	−0.584 (df = 139)	0.56	−0.976 (df = 139)	0.331
Rating of hotspot phase treated criticism sadness	−0.987 (df = 139)	0.325	−0.893 (df = 139)	0.373	−0.975 (df = 139)	0.331	−0.583 (df = 139)	0.561	−0.613 (df = 139)	0.541	−0.442 (df = 139)	0.659
Rating of hotspot phase future criticism anger	−0.138 (df = 129)	0.89	−0.524 (df = 129)	0.601	−0.439 (df = 129)	0.661	−1.956 (df = 129)	0.053	−1.644 (df = 129)	0.103	−0.892 (df = 129)	0.374
Rating of hotspot phase future criticism arousal	−0.427 (df = 129)	0.67	−0.144 (df = 129)	0.886	−0.179 (df = 129)	0.858	−1.451 (df = 129)	0.149	−1.591 (df = 129)	0.114	−1.639 (df = 129)	0.104
Rating of hotspot phase future criticism disgust	−0.206 (df = 129)	0.837	−0.540 (df = 129)	0.59	−0.374 (df = 129)	0.709	0.766 (df = 129)	0.445	0.272 (df = 129)	0.786	0.799 (df = 129)	0.426
Rating of hotspot phase future criticism fear	0.464 (df = 129)	0.644	0.414 (df = 129)	0.679	0.808 (df = 129)	0.42	1.449 (df = 129)	0.15	−0.209 (df = 129)	0.835	0.039 (df = 129)	0.969
Rating of hotspot phase future criticism guilt	0.726 (df = 129)	0.469	0.217 (df = 129)	0.829	0.337 (df = 129)	0.737	1.437 (df = 129)	0.153	0.108 (df = 129)	0.914	−0.025 (df = 129)	0.98
Rating of hotspot phase future criticism sadness	0.734 (df = 129)	0.465	0.688 (df = 129)	0.493	0.810 (df = 129)	0.419	1.493 (df = 129)	0.138	1.116 (df = 129)	0.266	1.373 (df = 129)	0.172
Rating of hotspot phase untreated criticism anger	−0.665 (df = 139)	0.507	−0.909 (df = 139)	0.365	−0.223 (df = 139)	0.824	−2.279 (df = 139)	0.024	−2.115 (df = 139)	0.036	−1.255 (df = 139)	0.212
Rating of hotspot phase untreated criticism arousal	0.538 (df = 139)	0.591	−0.399 (df = 139)	0.691	0.146 (df = 139)	0.884	−1.218 (df = 139)	0.225	−1.096 (df = 139)	0.275	−0.684 (df = 139)	0.495
Rating of hotspot phase untreated criticism disgust	−1.858 (df = 139)	0.065	−1.704 (df = 139)	0.091	−1.530 (df = 139)	0.128	−0.322 (df = 139)	0.748	0.255 (df = 139)	0.799	0.443 (df = 139)	0.658
Rating of hotspot phase untreated criticism fear	0.300 (df = 139)	0.765	−0.619 (df = 139)	0.537	0.066 (df = 139)	0.948	0.533 (df = 139)	0.595	−0.837 (df = 139)	0.404	−0.473 (df = 139)	0.637
Rating of hotspot phase untreated criticism guilt	0.177 (df = 139)	0.86	−0.704 (df = 139)	0.482	−0.370 (df = 139)	0.712	−0.115 (df = 139)	0.909	−0.889 (df = 139)	0.375	−0.724 (df = 139)	0.471
Rating of hotspot phase untreated criticism sadness	0.357 (df = 139)	0.722	−0.492 (df = 139)	0.623	0.171 (df = 139)	0.865	−0.015 (df = 139)	0.988	−0.390 (df = 139)	0.697	0.413 (df = 139)	0.68
Beck depression inventory	1.372 (df = 137)	0.172	1.165 (df = 137)	0.246	1.486 (df = 137)	0.14	2.386 (df = 137)	0.018	0.540 (df = 137)	0.59	0.101 (df = 137)	0.919
Frost multidimensional perfectionism scale	−0.389 (df = 137)	0.698	−1.592 (df = 137)	0.114	−1.683 (df = 137)	0.095	−1.855 (df = 137)	0.066	−1.304 (df = 137)	0.195	−1.164 (df = 137)	0.246
Leibowitz social anxiety scale	−0.411 (df = 137)	0.682	−0.232 (df = 137)	0.817	0.825 (df = 137)	0.411	−0.476 (df = 137)	0.635	−0.278 (df = 137)	0.781	0.582 (df = 137)	0.562
Performance failure appraisal inventory (fear of failure scale)	0.881 (df = 137)	0.38	0.824 (df = 137)	0.411	0.575 (df = 137)	0.566	0.572 (df = 137)	0.568	0.100 (df = 137)	0.921	0.769 (df = 137)	0.443
Pure procrastination scale	1.635 (df = 137)	0.104	−0.251 (df = 137)	0.802	−0.231 (df = 137)	0.817	0.139 (df = 137)	0.889	−0.669 (df = 137)	0.505	−1.167 (df = 137)	0.245
PTSD DSM-V scale	−1.123 (df = 137)	0.264					−0.596 (df = 137)	0.552				
General anxiety disorder	0.882 (df = 137)	0.379					−0.262 (df = 137)	0.794				
Panic disorder DSM-V scale	−0.468 (df = 137)	0.641					−0.222 (df = 137)	0.825				
Yale-Brown obsessive-compulsive scale	1.506 (df = 137)	0.134					−0.753 (df = 137)	0.453				

### Secondary outcomes (ITT analysis)

For secondary psychopathology measures (LSAS, PPS, Frost MPS, BDI-II, PTSD Checklist), analyses revealed no significant main effects of group or time × group interactions (all *p*s > 0.19). Main effects of time revealed modest overall reductions in social anxiety and perfectionism across all participants (η^2^_p_ range = 0.02–0.09). For example, LSAS scores showed a small but significant decline over time, *F*(2.90, 392.10) = 4.72, *p* = 0.003, η^2^_p_ = 0.034, with reductions from baseline to post-treatment (ΔM = −5.10, *p* = 0.041, d = 0.25). Similarly, Frost MPS scores decreased modestly, *F*(2.68, 363.15) = 5.18, *p* = 0.002, η^2^_p_ = 0.037. In contrast, PTSD symptoms (PTSD DSM-V Scale) and depressive symptoms (BDI-II) did not show significant time effects (*p*s > 0.10).

### Sensitivity analyses (comparison of ITT and PP)

Overall, results from the per protocol analyses closely mirrored the ITT findings, supporting the robustness of the main conclusions. In both approaches, participants showed substantial and sustained reductions in SCL and subjective distress during the treated criticism scenario, alongside modest improvements in secondary psychopathology. However, some notable differences emerged. First, in the SCL data, per protocol analyses yielded slightly stronger time effects and, in the recall procedure, a significant Time × Group interaction (η^2^_p_ ≈ 0.055), suggesting somewhat greater differentiation between interventions than in the ITT sample, where such effects were absent or only approached significance. Similarly, for salivary alpha-amylase, ITT analyses showed no evidence of change, whereas per protocol analyses indicated a small but significant overall reduction from baseline to post-treatment, consistent across groups. In the self-report data, both analytic approaches revealed large and stable time effects without group differences, though effect sizes were somewhat larger in the per protocol sample. For PFAI, ITT analyses showed only modest short-term reductions, whereas per protocol analyses revealed clearer and sustained decreases across follow-ups. Finally, secondary outcomes showed a comparable overall pattern across ITT and per protocol analyses, but in the per protocol sample more domains (e.g., generalised anxiety, PTSD, perfectionism) reached statistical significance.

Taken together, sensitivity analyses confirm that the primary ITT findings were robust, while per protocol results tended to produce slightly stronger effects and, in some instances, additional significant improvements. These differences likely reflect increased statistical power and reduced noise when analyses were restricted to participants with full protocol adherence.

### Prediction error as a mediator of therapy efficacy

We tested whether the effect of Group (rescripting vs. exposure) on Therapy Efficacy was mediated by PE. The direct effect of intervention type on SCL decrease to the hotspot after treatment was not significant (*B* = −0.58, SE = 5.89, *p* = 0.921). However, the indirect effect via prediction error was significant, *B* = 7.45, SE = 2.93, 95% CI [1.71, 13.19], *z* = 2.54, *p* = 0.011. Specifically, Rescripting elicited higher prediction errors than Exposure (*B* = 23.19, SE = 4.08, *p* < 0.001), and higher PE predicted stronger therapy effects (*B* = 0.32, SE = 0.11, *p* = 0.004).

**Figure 7:**
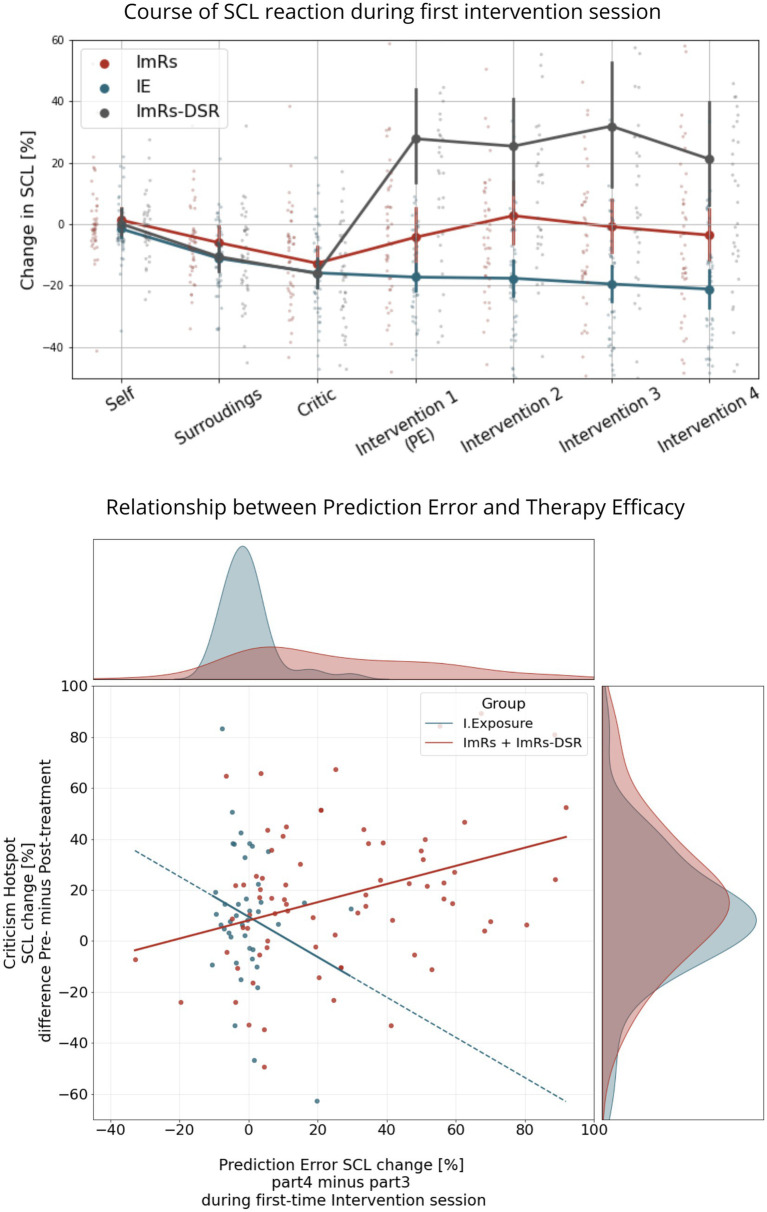
Course of SCL during first intervention session with PE visible during 4th part of scenario (above); Relationship between PE and Therapy Outcome, operationalised as a difference in Hotspot Criticism Reaction post- minus pre-treatment.

Although the total effect of intervention type on therapy outcome was not significant, mediation analysis revealed a significant indirect pathway via prediction error. This suggests that rescripting enhanced therapy effects only through its capacity to induce greater prediction error, rather than through a direct effect of the intervention itself (Figure 7).

## Discussion

The aim of the present randomised controlled trial (RCT) was to compare three imagery-based approaches targeting autobiographical memories of childhood criticism: Imagery Rescripting (ImRs), Imagery Rescripting with a 10-min break intended to disrupt the memory reconsolidation process (ImRs-DSR), and Imagery Exposure (IE) as an active control. The study evaluated both the short-term efficacy of these interventions and the durability of their effects over 3- and 6-month follow-up periods, as well as the generalisation of outcomes to other memories and novel contexts.

In addition to these primary objectives, two potential mechanisms of change were examined. The first was the role of prediction error (PE) in therapeutic change induced by rescripting. The underlying assumption was that violating participants’ expectations about the course of the imagined scene would facilitate memory updating and lead to greater reductions in emotional reactivity. The second was whether introducing a 10-min break between memory reactivation and rescripting – timed to coincide with the so-called reconsolidation window – would enhance the effectiveness of ImRs compared to its standard format.

A wide range of outcome measures was used to evaluate the interventions. Subjective measures included fear of failure scores and emotion ratings in response to autobiographical scenarios. Objective physiological indicators included skin conductance level (SCL) and salivary alpha-amylase (sAA), which are markers of sympathetic activation. Behavioral procedures assessed generalisation and durability of effects, including recall, renewal, and reinstatement.

Hypotheses 1 and 2 predicted that all three groups (IE, ImRs, ImRs-DSR) would show a significant reduction in emotional and physiological responses to the treated childhood criticism scenario after the intervention (which would be maintained over time, that is, at 3- and 6-month follow ups), with larger reductions in the rescripting groups - particularly in ImRs-DSR, due to presumed reconsolidation disruption.

Analyses across four time points (pre-treatment, post-treatment, 3-month follow-up, and 6-month follow-up) showed that all interventions produced marked and lasting reductions in physiological reactivity. Both SCL and sAA showed consistent post-treatment reductions, maintained over time (although the latter showed reduction only in per protocol analysis). SCL reductions persisted for at least 6 months and were resistant to renewal, spontaneous recovery (tested with recall), and reinstatement.

Self-report measures indicated a reduction in fear of failure (PFAI), although the effect was small and not consistently sustained at the 6-month follow-up. Negative emotions (sadness, fear, guilt, anger, disgust) and arousal decreased across all groups after treatment, although contrast analysis shows inferiority of ImRs-DSR. In addition, modest but significant decreases were observed in perfectionism (Frost MPS) and social anxiety (LSAS), suggesting that the interventions may have impacted broader domains of emotional and cognitive functioning. At the same time, no significant changes were found in depressive symptoms (BDI-II) or PTSD-related measures, highlighting the limited generalisation of effects. Notably, these questionnaire changes did not differ between groups, supporting the interpretation that common factors (e.g., emotional engagement, confrontation with aversive memories) were the primary drivers of these subjective improvements. Taken together with the physiological data, this pattern suggests that while subjective improvements generalised more broadly, not all domains of psychopathology were sensitive to the short-term, standardised imagery-based interventions.

Contrary to expectations, standard ImRs appeared more effective than ImRs-DSR in reducing subjective arousal, guilt, and sadness ratings for anticipation and hotspot phases of treatment scenarios, both post-treatment and at follow-ups. These effects, although modest, may indicate that the immediate integration of rescripting following memory reactivation is more beneficial than introducing a delay, at least when assessed through self-report indices of emotional reactivity (Strohm et al., 2021; Siegesleitner et al., 2020). One interpretation is that disrupting reconsolidation through a fixed 10-min delay may not enhance therapeutic outcomes in the context of autobiographical memories of criticism. This is consistent with recent debates on the boundary conditions of reconsolidation effects in humans, indicating that clinical applications may be more complex than suggested by basic laboratory paradigms (Bui and Milton, 2023). Moreover, the observation that standard ImRs showed more consistent subjective benefits relative to ImRs-DSR suggests that immediate intervention may facilitate deeper emotional processing, possibly through sustained engagement with the activated memory trace without an artificial interruption. Beyond these differences between the rescripting variants, all three interventions led to notable and lasting decreases in a wide range of self-reported negative emotions, including fear, sadness, anger, guilt, and disgust. These changes were observed both during the anticipation phase and at the hotspot, and were maintained for at least 6 months. This broad improvement suggests that participants not only experienced temporary relief but also underwent a more durable shift in emotional response to autobiographical criticism. At the same time, the absence of clear group differences in overall emotional change suggests that all interventions may have contributed to similar reductions in negative affect, although possibly through partially distinct mechanisms. In the case of rescripting, the decrease in emotional distress may reflect not only habituation but also a shift in the emotional meaning of the memory brought about by higher prediction error. In contrast, improvements following exposure may be primarily driven by habituation and increased tolerance of anticipated discomfort. However, based on subjective emotion data alone, it is not possible to determine whether distinct mechanisms underpinned these changes in different conditions. Reduction across a broad spectrum of negative emotional states may also have been influenced by nonspecific factors, such as intense emotional engagement during imagery, or the passage of time, which may have naturally diminished the emotional charge of the memories. Therefore, further research is needed for instance, including qualitative reports of participants’ experience or measures of belief change to better understand the processes responsible for emotional improvement across intervention types.

Regarding the hypothesis of superiority of ImRs over IE, the analyses revealed only subtle evidence. Specifically, participants in the IE condition demonstrated a rebound effect at the 6-month follow-up, with increased physiological arousal to their treatment scenarios during the reinstatement procedure, and to some extent renewal, compared to the ImRs group. These results suggest that the aversive potential of memories targeted by intervention may be reduced more stably by ImRs than IE. However, in our study we did not find evidence that this effect had any impact on a better subjective functioning. Moreover, it is important to acknowledge that the effects mentioned here were determined with exploratory contrast analyses and were not detected in main analyses, in which corrections for multiple comparisons were applied.

The lack of the expected superiority of ImRs-DSR over standard ImRs, and only subtle ImRs superiority over exposure, invites several possible explanations. The intensity and repetition of all interventions may have led to a ceiling effect, whereby participants reached the maximum detectable improvement with the measures used (Andrade, 2021). The non-clinical nature of the sample - individuals with elevated fear of failure but without severe psychopathology – may have increased responsiveness to general therapeutic factors such as emotional engagement, perceived support, and safe processing of distressing content, regardless of the specific method (Baier-Mosch et al., 2025). Further, mechanisms of change may have overlapped in different conditions: repeated engagement with emotionally salient autobiographical material might have activated common therapeutic processes such as habituation, emotion regulation, and corrective learning, which are not specific to rescripting (Cuijpers et al., 2019). It is conceivable that in the exposure condition, some participants may have spontaneously reinterpreted the meaning of the memory (akin to non-verbal rescripting), while rescripting conditions inevitably involved habituation processes typical of exposure (Cuijpers et al., 2019). Finally, the measures applied - emotional ratings and broad physiological indices – may have been insufficiently sensitive to detect subtle cognitive changes in meaning or belief structures, which might require more targeted assessments (Baier-Mosch et al., 2025).

Hypothesis 3 predicted that treatment effects would generalise from the targeted autobiographical memory to other, non-treated childhood criticism memories. It was expected that rescripting, especially ImRs-DSR, would show broader generalisation effects than exposure.

The findings partly supported this hypothesis. Participants reported lower negative emotions not only for the treated memory but also for non-treated memories of criticism. These improvements were visible in both rescripting and exposure groups and remained stable across follow-ups. However, physiological measures (SCL) did not show clear or consistent reductions for non-treated memories, suggesting that generalisation occurred mainly at the subjective level.

One explanation is that self-reported emotions are more flexible and easier to generalise than physiological responses, which are often more specific to particular stimuli (Hermans et al., 2006). Working with one highly relevant criticism memory may also have changed broader self-related beliefs, which then influenced how participants perceived their emotions when recalling other memories (Arntz, 2012). This could explain why emotional ratings changed, but physiological arousal did not (LeDoux and Pine, 2016). Another explanation is methodological: physiological measures usually require strong and standardised stimuli to detect transfer effects, while autobiographical memories vary in intensity and meaning, which increases variability and reduces sensitivity (Olatunji et al., 2007). It may also be accounted for recency effect, as scenarios used for generalisation were presented for the first time ever (future scenario), or after longer passage of time (untreated scenario) (Becker and Rohleder, 2019). This sudden presentation of scenarios, other than treated one, may lead to violation of expectancy. This may have caused an increased reaction on the physiological level, but subjectively, those scenarios were still less distressing.

Overall, the results indicate that treatment effects generalised more strongly in self-reported emotions than in physiological reactivity. This highlights the importance of including both subjective and physiological measures in future studies, as they may capture different but complementary aspects of change.

Hypothesis 4 addressed the role of prediction error (PE), the second key mechanism of change under investigation. It was expected that participants in the rescripting conditions (ImRs and ImRs-DSR) would experience higher PE than those in the exposure condition, due to the introduction of an unexpected, positive change in the course of the memory. PE was defined as the discrepancy between the expected negative outcome of the memory and its actual, more positive and surprising ending (Exton-McGuinness et al., 2015; Sinclair et al., 2021). We further predicted that higher PE would be associated with greater post-treatment improvement, operationalised as a larger reduction in physiological arousal to the treated memory. Since the content of the rescripting scenes was identical in both ImRs conditions, their PE levels were expected to be similar.

The mediation analyses confirmed that higher PE predicted greater therapeutic benefit, but only in the rescripting groups. In these conditions, the greater the surprise during the scene, the larger the reduction in physiological arousal after treatment. This finding aligns with models of emotional learning and memory reconsolidation, which propose that expectation violation promotes memory rewriting and reduces emotional impact (Lee et al., 2017). No such association was observed in the exposure group, where improvement likely stemmed from habituation - gradual reduction of emotional responses through repeated recall of negative content in a safe context (Craske et al., 2014). Similar to ImRs, the exposure condition did not include confrontation with the moment of criticism (hotspot), although its presentation might have been expected as it was primed by the pretreatment session. Therefore, it is possible that our IE participants also experienced PE, however the methodology of the current study did not allow us to detect it. Specifically, the experimental procedure might have been insufficient to detect the dynamics of PE during imagery exposure. Moreover, we did not include the passive control group that would allow comparison to no-intervention - and no PE whatsoever - which would compare reaction in the exposure group to no-intervention and detect it, even if the effect was small in comparison to rescripting groups. The current study also lacked subjective expectation measures, which would directly measure PE (however, using SCL as a psychophysiological measure of PE is grounded practice; Stemerding et al., 2022). As a caveat it has to be noted that, although the IE condition was intended to trigger anxious apprehension of criticism, we cannot exclude the possibility that the worst consequences participants expected was crying or falling apart.

The pattern of PE-outcome associations limited to the rescripting groups suggests that ImRs induces a salient violation of expectations at the critical moment: the anticipated escalation of criticism is abruptly replaced by a corrective or protective intervention. From the perspective of reconsolidation models, such a discrepancy between what is expected and what actually occurs serves as a signal to “open” the memory trace for updating and to integrate new, non-aversive information, thereby altering its emotional meaning (Sinclair et al., 2021; Exton-McGuinness et al., 2015). In contrast, no such mechanism may be present in IE, where symptom reduction is better explained by habituation and inhibitory learning - that is, the gradual weakening of responses through repeated exposure to aversive material in a safe context (Hamlett et al., 2023). This distinction helps explain why PE predicted therapeutic change only in rescripting.

It is also important to note that our operationalization of PE - a phasic increase in SCL following the hotspot replaced by therapeutic intervention - was naturally tailored to rescripting, where a sudden surprise element is present. In IE, where the memory proceeds in line with expectations, such a “surprise signal” was absent. Thus, the observed specificity of the PE-outcome relationship may partly reflect the measurement approach used in this study.

The absence of a superiority effect for ImRs-DSR over standard ImRs further suggests that synchronising the timing of change in the course of events with the reconsolidation window (a 10-min delay before rescripting) is insufficient to enhance efficacy. Instead, the quality and intensity of the PE (e.g., the strength of the surprise and its congruence with core self-schemas) may be more important. Moreover, introducing a delay may even have disrupted the continuity of emotional engagement, which is crucial for integrating the new information. This interpretation resonates with current debates on the boundary conditions of reconsolidation in clinical applications, where timing may be necessary but not sufficient unless accompanied by a meaningful expectation violation (Schiller et al., 2013; Bui and Milton, 2023; Woelk et al., 2022).

In summary, our findings most strongly support the view that rescripting induces a more stable decrease in physiological reactivity to aversive memories possibly through prediction error that promotes schema updating and revaluation, while exposure may rather work through habituation and inhibitory learning, without explicit violation of expectations. This distinction was amplified by our measurement of PE, which was particularly sensitive to the sudden, content-based surprise characteristic of rescripting.

### Limitations

Despite the strengths of this study, several limitations should be considered when interpreting the findings. The design required participants to repeatedly engage with the same autobiographical criticism memory across four sessions. While this ensured standardisation and intensive exposure, it may also have induced habituation effects independent of the specific mechanisms targeted by each intervention. In clinical practise, rescripting is typically applied flexibly across multiple memories, which may lead to qualitatively different patterns of change than those captured here.

Another important limitation concerns the operationalization of prediction error. In this study, PE was indexed solely through changes in skin conductance level within a predefined time window, reflecting the physiological component of surprise. This approach inevitably relied on experimenter-defined assumptions about when PE should occur, meaning that PE was not a purely objective measure but partly a construct imposed by the research design. Moreover, this narrow physiological definition neglects cognitive and affective dimensions of PE, such as shifts in interpretation or meaning. As a result, the role of PE may have been underestimated, and important aspects of its multidimensional nature left unexamined. At the same time, alternative operationalisations such as subjective ratings of PE also carry substantial methodological limitations (e.g., demand characteristics, expectancy effects), so our choice should be seen as a trade-off rather than a methodological flaw.

The measurement strategy more broadly also imposes constraints. Physiological markers such as SCL and sAA, although objective, are susceptible to variability unrelated to the intervention, including circadian fluctuations, physical activity, and individual differences in autonomic reactivity. Subjective ratings, while sensitive to change, remain vulnerable to demand characteristics and expectancy effects. Without complementary measures such as implicit tasks, narrative analyses, or neuroimaging indices, conclusions about the precise mechanisms underlying therapeutic change remain limited. In addition, subjective emotional ratings were collected retrospectively at the end of each session rather than during imagery, which may have increased susceptibility to recall bias and reduced the temporal precision of affective assessment.

The protocol itself was brief, highly standardised, and delivered through audio guidance, which enhanced internal validity and comparability across conditions. However, this format differs substantially from clinical practise, where rescripting is individualised, interactive, and embedded in the therapeutic relationship. The intensity of the intervention, four sessions over 2 weeks, may also have produced ceiling effects, leading to strong and relatively uniform improvements that obscured more subtle between-condition differences. Furthermore, ImRs is a personalised treatment that may be influenced by various nonspecific factors, such as the therapeutic alliance or an individual’s attitude toward their memories (for example, modifying a memory may be less effective in individuals with a strong sense of loyalty toward a parent) (Morina et al., 2017). In our study, we aimed to balance standardisation and ecological validity by having all scenarios recorded by the same psychotherapist, who had established a therapeutic relationship with participants during preparatory sessions. However, as with all laboratory designs, this approach remains limited by the inherently artificial conditions of the study (Siegesleitner et al., 2019).

Generalizability is further limited by the non-clinical sample. Although participants were selected for elevated fear of failure, they did not present with severe psychopathology. It remains uncertain whether the same mechanisms would operate in patients whose autobiographical memories are more deeply entrenched and emotionally disruptive. The 6-month follow-up provides valuable evidence of medium-term stability, yet the durability of effects beyond this time frame is unknown.

Finally, the analytic approach focused primarily on group-level averages and linear patterns of change. It is possible that meaningful processes occurred at the individual level, with only a subset of participants experiencing PE strongly enough to drive reconsolidation-related updating. Without idiographic analyses or modelling of heterogeneous trajectories, important nuances in treatment response may have been overlooked.

Another limitation relates to sample adherence. Dropout was relatively low (≈8%), which may have enhanced internal validity and statistical power. However, this low attrition rate likely reflects the non-clinical nature of the sample, the relatively short and structured intervention, and the financial incentives provided. In clinical populations with higher levels of distress or comorbidity, dropout is typically higher, which may affect both feasibility and effect sizes. Thus, the present results may somewhat overestimate adherence and tolerability compared to real-world clinical contexts.

It is also possible that the reason for the lack of any substantial group effects is due to the methodological shortcomings of the study. The first limitation is that the hotspot was not presented along with anticipation during the intervention. Previous study (Dibbets and Arntz, 2016) showed that using ImRs after the hotspot (called “late rescripting”) provided better results than rescripting without the presentation of the hotspot (called “early rescripting”). In our study, we provided early rescripting, which was a decision we made (and that was consulted with and reviewed by one of the authors from Dibbets and Arntz, 2016) to challenge the treatment effects using renewal and reinstatement procedures, which required to treat the hotspot (that we conceptualised as an unconditioned stimulus, UCS) as a separate stimulus. Worth noting, the study published by Dibbets and Arntz (2016) focused on therapeutic effects after 1 intervention session, while in our study the intervention was presented four times in 2 weeks and provided evidence of efficacy in this setting, thus differences between early and late rescripting may vary depending on how it was provided.

Furthermore, the fact that the hotspot was not presented during the intervention, creates a methodological discrepancy between our study design and one conducted by Schiller et al. (2013). In that study disruption of reconsolidation was performed by intervening after recalling the source of the fear, that is the unconditioned stimulus. In our study we reactivated the memory of criticism, however, we did not present the unconditioned stimuli, only the conditioned ones (reactivation). Thus, the lack of evidence for the beneficial effect of reconsolidation may be due to this methodological difference. Finally, the sample of the study was relatively small, and the study may have been underpowered to detect subtle group × time effects. Furthermore the study sample was overrepresented by women, which limits interpretability of results, but is common in studies of young adults (Dickinson et al., 2012).

### Clinical implications and future directions

The present findings hold several implications for clinical practise and the future refinement of memory-focused interventions. The observation that both exposure and rescripting produced substantial and durable reductions in distress highlights the potential utility of imagery-based methods more broadly, even outside of clinical populations. This underscores that repeated, emotionally engaging confrontation with autobiographical memories, when conducted in a safe and structured manner, can generate meaningful change irrespective of the specific technique employed.

At the same time, the unique association between prediction error and treatment benefit in the rescripting conditions points to the clinical value of deliberately eliciting expectation violation. Therapists may consider structuring rescripting interventions to maximize PE by explicitly activating patients’ negative expectancies and then introducing corrective, emotionally salient outcomes. Developing reliable, multidimensional measures of PE – integrating physiological, cognitive, and subjective indices – would be a crucial step toward systematically embedding this mechanism into clinical protocols.

Considering the fact that the effects were largely comparable across conditions may indicate the important role of common therapeutic processes, such as emotional engagement, corrective emotional experiences, and the safe processing of distressing autobiographical material. These common factors may be particularly important in settings where resource constraints make simpler interventions, such as structured exposure, more feasible than full rescripting. At the same time, understanding the unique contribution of rescripting remains important, especially for patient groups where entrenched maladaptive schemas and rigid self-beliefs are central.

Future research should extend these findings in several directions. Clinical trials with patients suffering from trauma-related disorders, personality pathology, or chronic self-critical tendencies are needed to establish the external validity of the mechanisms observed here. Long-term follow-ups beyond 6 months are essential to evaluate whether reconsolidation-based updating leads to enduring restructuring of memory and self-schemas, or whether effects attenuate over time. The integration of idiographic approaches, implicit measures, and neurobiological methods such as functional neuroimaging could provide more fine-grained insights into the processes by which exposure and rescripting exert their effects.

Finally, advancing translational work on reconsolidation and prediction error has the potential to move psychotherapy toward greater mechanistic precision. By identifying when and for whom PE-based interventions are most effective, clinicians may be able to tailor treatments that not only reduce distress in the short term but also target the deeper cognitive and emotional structures that sustain psychopathology.

## Data Availability

The datasets presented in this study can be found in online repositories. The names of the repository/repositories and accession number(s) can be found in the article/Supplementary material.
