# Supplementary material for: Imagine yourself as a little girl…—efficacy and psychophysiology of imagery techniques targeting adverse autobiographical childhood experiences- multi-arm randomised controlled trial
Source: Front Psychol. 2026 Jan 16;16:1710963. doi: 10.3389/fpsyg.2025.1710963 (PMC12857063; doi:10.3389/fpsyg.2025.1710963)
Supplement: Supplementary file 1 [file Supplementary_file_1.docx]

# Appendix A

[Appendix A 1](#_jj6aqqetk6u3)

[Experimental procedure 2](#_ektknr40endv)

[Standardized framework for memory activation 3](#_yve6bdq1d9z4)

[Example neutral scenario 3](#_u14xvtts4d68)

[Criticism scenario 3](#_emp6w79uap0j)

[ImRs imagery treatment scenario 3](#_ylw0nq4qspox)

[IE imagery exposure treatment scenario 4](#_daxaqqr5ushc)

[Subjective measures 4](#_f7epp6lsgfn4)

[Primary measures (subjective) 4](#_639xwjq1ida)

[Secondary measures (subjective) 5](#_a7v37qt649nr)

[Intention-to-Treatment Analyses 6](#_fiv7idw7z3kh)

[Treated Criticism Scenario – SCL Response (ITT analysis) 6](#_jm4xcyyt5n8u)

[Salivary Alpha-Amylase (sAA) 7](#_shnv1w5p7o7j)

[Subjective response during anticipation of the treated criticism scenario (ITT analysis) 7](#_kegaaxibotzh)

[Subjective response during the hotspot phase of the treated criticism scenario (ITT analysis) 8](#_pwch7oddhykp)

[PFAI (Performance Failure Appraisal Inventory) 9](#_twa8btc96c74)

[Generalizability SCL – Untreated & Future criticism scenarios SCL (ITT analysis) 9](#_hfuwj37lnc61)

[Generalizability subjective measures – Future Criticism Scenario (ITT Analysis) 10](#_fjsyd925qfld)

[Subjective Measures – Untreated Criticism Scenario (ITT Analysis) 11](#_o331wvq9h8be)

[Secondary Outcomes – ITT Analysis 13](#_6pu4qnpf06lb)

[Per Protocol analyses 13](#_7z4nvpgrj4l5)

[Short- & Long-term Treatment Effects - Treated Criticism Scenario SCL Response (analyses per protocol) 13](#_fh4x7389sh00)

[Salivary Alpha-Amylase (sAA) (Per Protocol analysis) 14](#_acsw7u57gh9)

[Subjective response during anticipation of the treated criticism scenario (Per Protocol analysis) 14](#_n1299lr7y4i9)

[Emotional Reactivity During Hotspot (Per Protocol Analysis) 15](#_qsheddq0vmj1)

[PFAI (Performance Failure Appraisal Inventory, Per Protocol analysis) 16](#_ulzlfmsjm3st)

[Generalizability SCL Response– Untreated & Future criticism scenarios (Per protocol analysis) 16](#_eehb0vcc7ok1)

[Subjective measures Untreated & Future criticism scenarios (Per Protocol analysis) 17](#_yqzhk617ingu)

[Secondary Outcomes – Per Protocol 17](#_3z0kpcj3kixs)

[Questions regarding therapy attendance 19](#_k2zv0qehe8hx)

[Verification of punitive parental experiences 20](#_6cc4ql1b7uls)

[References 22](#_yrli32s9et67)

[Supplementary Table 1. Per Protocol Analysis 28](#_nosznw6mdbtg)

[Supplementary Table 2. Descriptive data Per Protocol Mean (SD) 38](#_8em0e1fauwu6)

[Supplementary Table 3 Per Protocol Contrast Analysis 44](#_2kukb08u7drb)

##

## Experimental procedure

Preparatory meetings with therapists were conducted online. Pre- and post-treatment sessions were scheduled between 8 am-11 am, due to daily fluctuations of sAA (with several deviations, when sessions were rescheduled due to participants' availability). Hourly timing of intervention sessions was unrestricted (however, we ensured that sessions were scheduled in 2 weeks between pre-and post-treatment, every 2 days if possible).

After all scenarios were fragmented and prepared as standardized audio recordings (see below), subjects were invited to the laboratory. On the first day (pre-treatment), subjects filled out questionnaires (PPS, LSAS, FROST), after which they were instructed to sit in a semi-dark room in front of a computer screen, where all instructions & ratings were to be presented during the study. Then, the baseline salivary sample was collected. Next, SCL electrodes were placed on the index and middle fingers of the non-dominant hand, and a 2-minute SCL baseline was collected, during which subjects were asked to sit and rest. Then the instructions regarding the presentation of scenarios were displayed. To acquaint subjects with the scenarios, one of 3 trial neutral stories was randomly presented. Afterward, experimental scenarios (anticipation & hotspot) were presented. The first story was always the criticism scenario, that was later used for treatment. There was a 10-minute break after the presentation of this scenario, during which participants completed two questionnaires (GSES, MMCS). After the break, we collected a sample of sAA, and then the second scenario of criticism was presented, followed by negative and neutral scenarios played in a random order. Once the presentation of all scenarios finished, subjects were asked to listen to each scenario once more and rate each of its fragments for valence, immersion, focus, arousal, and basic emotions.

Following the pre-treatment session, subjects participated in 4 meetings that took place in the same room, during which they listened to a rescripted version of one of their criticism scenarios (“treatment scenario”, see Imagery Scenarios & Interventions) with or without reconsolidation period (see Imagery Scenarios & Interventions). Next, the scenario was rated as in the pre-treatment session.

After 2 weeks of treatment, the post-treatment session was conducted. This session began in the same room as previous meetings (spontaneous recovery procedure). Participants first listened to their criticism scenario targeted during treatment, followed by the other criticism scenario, and then negative and neutral scenarios presented in random order. Next, subjects were asked to change the room (second context), and the same set of scenarios was presented (renewal). During the spontaneous recovery and renewal procedures scenarios were presented without hotspots. Afterward, participants went back to the original room and listened to the criticism part from the scenario targeted during treatment (“hotspot”; reinstatement), which was followed by a 10-minute break, during which they completed a set of questionnaires (as at pre-treatment session), which ended with a collection of sAA sample. Then, they listened to treated and untreated criticism scenarios (without hotspot, only anticipation). Next, neutral, negative, and future criticism scenarios were presented in a random order. The post-treatment procedure was repeated after 3 months (3-month follow-up) and after 6 months (6-month follow-up), without the collection of the sAA sample. At the end of each time point scenarios were rated as at pre-treatment, after 6-month follow-up we additionally collected questionnaire data as in screening.

##

##

## Standardized framework for memory activation

To make memories comparable between subjects, we standardized each memory to the following framework:

1. *Self imagination & surroundings* - the description of the subject who is imagining the memory, starting with normalized “Imagine yourself as an (age) years old girl/boy…”; it is important that it is presented as if it was happening in the present moment; elements of modalities must appear (You hear…, You see…, You can smell…); furthermore, in this scenario, the subject is addressed as a name of him/her when the memory is taking the place
2. *Surroundings -* further description of the surroundings*;*
3. *Person -* description of the person, that interacts with the client; in our treatment condition we are focusing on Parental criticism
4. *Interaction with the person (Hot Spot/Spot) -* description of the interaction with the person, the hot spot of memory, thus in treatment condition it is a moment in which the criticism takes place and which is rescribed in ImRs condition; in the neutral condition, this part is describing neutral, daily interaction with a person

### Example neutral scenario

1. Imagine that you are in the building of your work. It's a typical day at work. It's late afternoon. It is stuffy.
2. You are sitting in a very comfortable chair by the window at the end of the row. You are wearing a blue work shirt. Behind you is a wall with the company logo.
3. You just have a break between conversations with clients. In front of you, you can see your colleague Stefan, who has just finished talking to the client.
4. Stefan is upset and complains about the client. You are amused by his anger. After a moment, you laugh together and talk. You feel employee solidarity.

### Criticism scenario

1. Imagine that you are six years old. You have dark curly hair. You are a good, but plump girl. You are in the hallway of your grandparents' flat.
2. The hallway is a long room with doors leading to other rooms, and there are some shoes on the floor.
3. Mum is standing in the hallway. She has her hair let down, and she is smartly dressed. She is calm. She raises her eyes and looks at you.
4. Your mum raises her voice, “Why are you not dressed yet? You look awful!” You feel sad and humiliated.

### ImRs imagery treatment scenario

1. Imagine that you are six years old. You have dark curly hair. You are a good, but plump girl. You are in the hallway of your grandparents' flat.
2. The hallway is a long room, there are doors leading to other rooms, and there are some shoes on the floor.
3. Mum is standing in the hallway. She has her hair let down, and she is smartly dressed. She is calm. She raises her eyes and looks at you.
4. Now, imagine me, therapist coming into the hallway. I am addressing your mum calmly but firmly: „Do not try to criticize Sylwia!”
5. Do not try to decide about things that are important to Sylwia. Please, appreciate her needs and feelings. Sylwia is a sensitive child.
6. Now, I am addressing you: "Sylwia, nobody has the right to decide about your things. You have the right to get attached to them.”
7. You are a smart, sensitive girl. You have the right to your feelings, and no one has the right to criticize you.

### IE imagery exposure treatment scenario

1. Imagine that you are six years old. You have dark curly hair. You are a good, but plump girl. You are in the hallway of your grandparents' flat.
2. The hallway is a long room, there are doors leading to other rooms, and there are some shoes on the floor.
3. Mum is standing in the hallway. She has her hair let down, and she is smartly dressed. She is calm. She raises her eyes and looks at you.
4. Mom is looking at you. You lean out to see her. You can see that her face is angry. She is furious.
5. You feel fear rising in you. You're breaking down. You are feeling small and weak when your mother is towering over you.
6. You are slouching, and your hands are sweating. Breathing begins to accelerate. You are trying to hold tears back. Crying is bad.
7. You think you should dress better. You think your mother has no use of you; she cannot rely on you.

## Subjective measures

Primary outcome was fear of failure was verified with The Performance Failure Appraisal Inventory (PFAI; Conroy et al., 2002; Polish translation: Golińska, 2017), depression with Beck Depression Inventory (BDI; Beck et al., 1987; Polish translation: Łojek & Stańczak, 2019), obsessive-compulsive disorder with Y-BOCS (Goodman et al., 1989; Polish translation: Nowakowska-Domagała et al., 2016), personality disorders SCID (First et al., 2016, Polish translation: Zawadzki, 2017), agoraphobia, GAD and PTSD with DSM scales (Craske et al., 2013a, 2013b, 2013c, 2014d; own translation), social anxiety with Liebowitz Social Anxiety Scale (LSAS; Heimberg et al., 1999; Polish translation: Chodkiewicz & Miniszewska, 2015) We also used the Mini-Mental State Examination (Cockrell & Folstein, 2002) for an interview regarding mental problems. Further, imagery scenarios were rated for arousal, evoked emotions (sadness, guilt, fear, anger, disgust), focus, and immersion using a 9-point Likert scale (0-very low, 9-very high). Our self-report primary self-report outcomes were fear of failure and arousal ratings. Other questionnaires (depression, social anxiety, etc.) and ratings (emotions, focus, etc.) were our secondary outcomes .

## Primary measures (subjective)

**The Performance Failure Appraisal Inventory** (PFAI; Conroy, 2001, Polish translation: Golińska, 2017) was used to assess fear of failure. Polish adaptation is a 35-item questionnaire that measures the strength of subjective beliefs about the consequences of failure. The PFAI has five subscales: fear of experiencing shame and embarrassment; fear of devaluing one’s self-esteem; fear of having an uncertain future; fear of important others losing interest, and fear of upsetting important others.

**Subjective ratings at the end of all sessions**- participants were asked to evaluate each fragment of the presented scenarios according to several measures: arousal, sadness, guilt, fear, anger, and disgust on a 9-point Likert scale (very low-very high).

## Secondary measures (subjective)

**Beck Depression Inventory** second edition (BDI-II; Beck, Steer, & Brown, 2005; Polish translation: Łojek & Stańczak, 2019) BDI-II is a self-report scale using 21 items regarding the presence and strength of depression symptoms.

**Yale-Brown Obsessive-Compulsive** self-report severity scale Y-BOCS-SR (Goodman et al., 1989; Polish translation: translation made by the authors using a standard back-translation method, 2020) a 10-item, self-report questionnaire created to evaluate OCD severity.

**The Alcohol Use Disorders Identification Test** AUDIT (Saunders et al., 1993; Polish translation: Babor et al., 1998) is a self-reported tool containing 10 items used to assess recent alcohol consumption, alcohol dependence symptoms, and problems related to alcohol consumption.

**Drug Abuse Screen Test** DAST 10 (Skinner, 1982; Polish translation: translation made by the authors using a standard back-translation method, 2020) is a self-reported questionnaire to detect drug use disorders.

**DSM Scales** (Craske et al., 2013a, 2013b, 2013c, 2014d, own translation); is a set of brief dimensional self-rating questionnaires for social anxiety disorder, agoraphobia, panic disorder, generalized anxiety disorder, and Post Traumatic Stress Symptoms (American Psychiatric Association, 2013, Polish translation: translation made by the authors using a standard back-translation method, 2020). Each scale consists of 10 items relating to the thoughts, feelings, and behaviors the subjects have experienced in the last 7 days. The answers are marked on a 4-point Likert scale (0=never, 4=all the time).

**SCID-5-PD** (First et al., 2016, Polish translation: Zawadzki, 2017) is a semistructured clinical interview that evaluates DSM-5 personality disorders under three clusters of A, B, and C, and other specific personality disorders.

**M.I.N.I. Mini International Neuropsychiatric Interview** (Sheehan, et al., 1998, Polish translation: Masiak & Przychoda, 1998) is a short structured interview for DSM IV and ICD 10 disorders, used to assess mental disorders: major depressive disorder, dysthymic disorder, suicidality, mania, panic disorder, agoraphobia, social phobia, specific phobia, obsessive-compulsive disorder, post-traumatic stress disorder, alcohol dependence/abuse, drug dependence/abuse, antisocial personality disorder.

**Liebowitz Social Anxiety Scale** (LSAS; Liebowitz, 1987) is a 24-item scale to evaluate fear and avoidance in social situations such as social interaction, public speaking, being observed by others, eating and drinking in public.

**Pure Procrastination Scale** (PPS; Steel, 2010; Polish adaptation by Cieciuch and Stępień, with alternations introduced by Stępień and Topolewska, 2014, pp. 152–154) is composed of 12 items, which measures three components of procrastination: decisional delay, implemental delay and delays in lateness/timeliness.

**Frost Multidimensional Perfectionism Scale** (Frost MPS; Stöber, 1998; Polish translation: Piotrowski and Bojanowska, 2021) was used to assess overall perfectionism and its dimensions: Personal Standards, Organization, Concern Over Mistakes, Doubts About Actions, Parental Expectations, and Parental Criticism.

**Subjective ratings at the end of all sessions**- participants were asked to evaluate each fragment of the presented scenarios according to several measures: immersion, focus, emotions (happiness, sadness, guilt, fear, anger, disgust) on a 9-point Likert scale (very low-very high), and valence (very negative-very positive).

## Intention-to-Treatment Analyses

### Treated Criticism Scenario – SCL Response (ITT analysis)

Physiological responses during the anticipation and peak emotional phases of the treated criticism scenario were examined across four time points (pre-treatment, post-treatment, 3-month follow-up, 6-month follow-up) using repeated-measures ANOVAs with Time (4 levels) and Condition (IE, ImRs, ImRs-DSR) as factors.

For anticipation phase, in the recall procedure, a robust main effect of time was observed, F(2.54, 342.52) = 57.75, p < .001, η²_partial = .30, indicating substantial reductions in anticipatory arousal. The Time × Condition interaction approached significance, F(5.07, 342.52) = 1.97, p = .081, η²_partial = .028, suggesting slight variation between groups. Post hoc tests showed significant decreases from pre-treatment to post-treatment (ΔM = 15.77, SE = 1.58, t = 10.01, pBonf < .001, d = 1.29), 3-month follow-up (ΔM = 14.05, pBonf < .001), and 6-month follow-up (ΔM = 16.15, pBonf < .001), with no significant differences between post-treatment and follow-ups.

In the renewal procedure, anticipatory SCL also decreased significantly, F(2.33, 314.19) = 108.08, p < .001, η²_partial = .45. The interaction with Condition was not significant, F(4.65, 314.19) = 1.09, p = .366, η²_partial = .016. Reductions from pre-treatment to post-treatment (ΔM = 20.10, pBonf < .001), 3-month follow-up (ΔM = 21.77, pBonf < .001), and 6-month follow-up (ΔM = 20.40, pBonf < .001) were large and consistent across groups.

In the reinstatement procedure, we again found a strong time effect for anticipatory SCL, F(2.11, 285.10) = 98.90, p < .001, η²_partial = .42. The Time × Condition interaction was again non-significant (p = .384), and post hoc comparisons confirmed large reductions from baseline to all subsequent time points across all groups (e.g., pre-treatment–post-treatment: ΔM = 17.55, d = 1.44, pBonf < .001). Between-group comparisons did not reveal any consistent superiority.

In the hotspot phase, which represents the peak of emotional reactivity, we observed a significant main effect of time, F(2.60, 342.52) = 18.16, p < .001, η²_partial = .119. SCL decreased significantly from pre-treatment to post-treatment (ΔM = 14.63, SE = 2.35, t = 6.24, pBonf < .001, d = 0.70), 3-month follow-up (ΔM = 15.11, pBonf < .001), and 6-month follow-up (ΔM = 12.11, pBonf < .001). No significant Time × Condition interaction was found, F(5.19, 350.55) = 1.68, p = .135, η²_partial = .024, although descriptively, the ImRs group showed the most pronounced reduction at follow-up, while IE rebounded slightly at 6-month follow-up.

Across all procedures, the ITT analysis confirms robust and lasting reductions in SCL following intervention, consistent across the three experimental groups. Although some numerical trends were suggesting stronger reductions in the rescripting conditions, particularly ImRs-DSR, these did not reach statistical significance in interaction terms. Reductions remained stable over time, indicating durable physiological benefits of the interventions.

### Salivary Alpha-Amylase (sAA)

Levels of salivary alpha-amylase (sAA) did not show consistent evidence of treatment-related change. A main effect of time was not significant, F(2.67, 338.70) = 1.02, p = .378, η²_partial = .008, and no group or time × group interaction effects emerged (all ps > .23). This indicates that the intervention procedures had no measurable impact on physiological stress reactivity as indexed by sAA across the study period.

### Subjective response during anticipation of the treated criticism scenario (ITT analysis)

A 3 (group: IE vs. ImRs vs. ImRs-DSR) × 4 (time point: pre-treatment [pre-treatment], post-treatment [post-treatment], 3-month follow-up [3-month follow-up], and 6-month follow-up [6-month follow-up]) repeated-measures ANOVA was conducted for each self-reported emotion assessed during the anticipation phase of the treated criticism scenario.
 The analyses revealed significant main effects of time for all negative emotional responses, reflecting consistent reductions in emotional reactivity from pre- to post-treatment and across follow-up time points.

**Sadness.** A strong time effect was observed, F(1.59, 220.64) = 101.71, p < .001, η²_partial = .423. Post hoc comparisons (Bonferroni corrected) confirmed significant decreases from pre-treatment to post-treatment (ΔM = 1.74, SE = 0.16, t = 11.08, pBonf < .001, d = 1.24), 3-month follow-up (ΔM = 1.84, t = 11.32, pBonf < .001), and 6-month follow-up (ΔM = 1.81, t = 10.78, pBonf < .001). No significant differences were found between post-treatment and follow-up time points. The only significant time × group interaction was observed for sadness, F(3.18, 220.64) = 3.41, p = .016, η²_partial = .047, indicating greater post-treatment decreases in the ImRs and IE groups compared to the ImRs-DSR group.

**Fear.** A robust main effect of time was found, F(1.80, 249.48) = 82.54, p < .001, η²_partial = .184. Post hoc tests showed significant reductions from pre-treatment to post-treatment and both follow-ups, with no differences between follow-up assessments. No significant group or interaction effects were observed (ps > .34).

**Guilt.** Ratings of guilt significantly decreased over time, F(1.56, 216.36) = 76.64, p < .001, η²_partial = .355. Post hoc comparisons indicated large and sustained reductions from pre-treatment to post-treatment and across follow-ups. No significant group or interaction effects emerged (ps > .15).

**Anger.** A significant main effect of time was observed, F(2.10, 291.38) = 36.82, p < .001, η²_partial = .209. Ratings decreased significantly from pre-treatment to post-treatment (ΔM = 1.03, SE = 0.16, t = 6.25, pBonf < .001, d = 0.80), with effects remaining stable at both follow-ups. No group or interaction effects were found (ps > .57).

**Disgust.** Ratings significantly declined across time, F(2.03, 282.64) = 24.09, p < .001, η²_partial = .148, with stable effects across follow-ups. Neither group differences nor interaction effects were significant (ps > .27).

**Arousal.** A significant main effect of time emerged, F(1.95, 270.77) = 30.16, p < .001, η²_partial = .178. Pairwise comparisons showed significant decreases from pre-treatment to post-treatment (ΔM = 1.08, SE = 0.19, t = 5.68, pBonf < .001, d = 0.57), 3-month follow-up (ΔM = 1.34, t = 6.70, pBonf < .001), and 6-month follow-up (ΔM = 1.26, t = 6.30, pBonf < .001), with no significant differences between follow-up points. Group and interaction effects were non-significant (ps > .77).

The only significant time × group interaction was observed for sadness, F(3.18, 220.64) = 3.41, p = .016, η²_partial = .047, indicating greater post-treatment decreases in the ImRs and IE groups compared to the ImRs-DSR group. No significant main effects of group were observed for any emotion (all ps > .18), nor were any other significant time × group interaction effects detected (all ps > .13). These findings indicate that all three interventions led to comparable and sustained reductions in subjective negative affect during the anticipation of autobiographical criticism memories, and these effects remained stable across the 3- and 6-month follow-ups.

### Subjective response during the hotspot phase of the treated criticism scenario (ITT analysis)

To assess emotional reactivity during the most emotionally intense part of the criticism scenario (hotspot), we conducted repeated-measures ANOVAs with 3 (group: IE, ImRs, ImRs-DSR) × 4 (time point: pre-treatment [pre-treatment], post-treatment [post-treatment], 3-month follow-up [3-month follow-up], and 6-month follow-up [6-month follow-up]) factors for self-reported emotions using the intention-to-treat (ITT) sample. Significant main effects of time point were found for all assessed emotions, indicating a robust and sustained reduction in negative affect following intervention.

Arousal. A significant time effect was also found, F(2.05, 285.99) = 18.48, p < .001, η²_partial = .117. Ratings decreased from pre-treatment to post-treatment (ΔM = 0.85, SE = 0.22, t = 3.87, pBonf = .001, d = 0.36), with reductions remaining stable at both follow-ups.

Anger. A significant main effect of time was observed, F(1.99, 276.47) = 29.51, p < .001, η²_partial = .175. Post hoc comparisons (Bonferroni corrected) confirmed significant reductions from pre-treatment to post-treatment (ΔM = 1.56, SE = 0.22, t = 7.21, pBonf < .001, d = 0.70), 3-month follow-up (ΔM = 1.39, t = 5.64, pBonf < .001), and 6-month follow-up (ΔM = 1.56, t = 6.20, pBonf < .001). No significant differences were observed between the follow-up assessments.

Fear. Ratings of fear decreased significantly over time, F(2.04, 283.49) = 70.83, p < .001, η²_partial = .338. Post hoc tests showed robust reductions from pre-treatment to post-treatment (ΔM = 1.98, SE = 0.20, t = 9.87, pBonf < .001, d = 0.85) as well as to 3-month follow-up and 6-month follow-up, with no evidence of rebound effects.

Sadness. A large and sustained reduction was found, F(2.22, 308.54) = 106.99, p < .001, η²_partial = .435. Ratings dropped significantly from pre-treatment to all subsequent time points (e.g., pre-treatment to post-treatment: ΔM = 2.51, SE = 0.20, t = 12.72, pBonf < .001, d = 1.13) and remained stable through the 6-month follow-up.

Guilt. A strong main effect of time was observed, F(2.10, 292.15) = 83.09, p < .001, η²_partial = .374. Post hoc comparisons confirmed significant decreases from pre-treatment to post-treatment, 3-month follow-up, and 6-month follow-up, with no significant differences between follow-up assessments.

Disgust. Ratings significantly declined across time, F(2.23, 310.35) = 30.94, p < .001, η²_partial = .182, with post hoc tests showing consistent decreases from pre-treatment to post-treatment, 3-month follow-up, and 6-month follow-up.

Summary. No significant main effects of group were observed for any emotion (all ps > .23), nor were any significant time × group interactions detected (all ps > .07). These findings indicate that all three interventions produced comparable and sustained reductions in subjective emotional reactivity during the core traumatic memory segment, and these effects remained stable across the 3- and 6-month follow-ups.

### PFAI (Performance Failure Appraisal Inventory)

Analyses of the fear of failure (PFAI) did not reveal significant group or time × group interaction effects (all ps > .14). A main effect of time was observed, F(2.55, 354.12) = 6.48, p < .001, η²_partial = .045, reflecting a small overall reduction in PFAI scores across assessments. Post hoc comparisons indicated that reductions from pre-treatment (pre-treatment) to post-treatment (post-treatment; ΔM = –3.21, p = .009, d = 0.28) and to the 3-month follow-up (3-month follow-up; ΔM = –3.85, p = .006) were significant, but changes did not remain robust at the 6-month follow-up (6-month follow-up, p = .087). These results suggest modest improvements in fear of failure following intervention, but without clear differentiation between conditions.

### Generalizability SCL – Untreated & Future criticism scenarios SCL (ITT analysis)

To test the generalization of treatment effects, we conducted ANOVAs with 3 (group: IE, ImRs, ImRs-DSR) × 4 (time point: pre-treatment [pre-treatment], post-treatment [post-treatment], 3-month follow-up [3-month follow-up], and 6-month follow-up [6-month follow-up]) factors for self-reported emotions using the intention-to-treat (ITT) sample.

For the untreated criticism scenario, no consistent evidence for time effects was found in the anticipation phase. In the recall procedure, a weak but significant main effect of time was observed, F(2.81, 375.99) = 3.71, p = .014, η²_partial = .027, driven by a significant reduction in SCL from pre-treatment to post-treatment (ΔM = –4.07, SE = 1.24, t = –3.29, pBonf = .008, d = 0.35). However, no further changes were observed at follow-up time points (all pBonf > .67), and no significant Time × Condition interaction was detected.

In the renewal and reinstatement procedures, no significant main effects of time were found (Fs < 0.9, ps > .45), indicating stable SCL levels throughout. There were no significant effects of condition or time × condition interactions in any of the procedures. These results suggest that physiological arousal in response to untreated autobiographical criticism remained largely unaffected by the interventions across all groups.

In contrast, the anticipation of future criticism showed a robust increase in physiological arousal over time. A significant main effect of time was observed, F(1.29, 160.72) = 52.14, p < .001, η²_partial = .296. Post hoc tests revealed significant increases in SCL from pre-treatment to post-treatment (ΔM = 10.21, SE = 1.22, t = 8.38, pBonf < .001, d = 1.29), 3-month follow-up (ΔM = 8.73, t = 7.00, pBonf < .001), and 6-month follow-up (ΔM = 8.56, t = 7.07, pBonf < .001). SCL remained elevated across all follow-ups. No significant time × condition interaction or between-group effects were detected, indicating comparable trajectories in all three groups.

A similar pattern emerged during the hotspot phase of the future criticism scenario. Again, a strong main effect of time was observed, F(1.29, 160.72) = 52.14, p < .001, η²_partial = .296. SCL values increased significantly from pre-treatment to post-treatment (ΔM = 10.21, SE = 1.22, t = 8.38, pBonf < .001, d = 1.29), 3-month follow-up (ΔM = 8.73, t = 7.00, pBonf < .001), and 6-month follow-up (ΔM = 8.56, t = 7.07, pBonf < .001). As with anticipation, no group or interaction effects reached significance.

These results indicate that while physiological reactivity to untreated autobiographical criticism scenarios remained largely stable, responses to imagined future criticism increased significantly following intervention and remained elevated over time. This rise in SCL was not moderated by intervention type, and may reflect increased salience or anticipatory threat appraisal of imagined future events that were not directly targeted by the intervention procedures.

### Generalizability subjective measures – Future Criticism Scenario (ITT Analysis)

In the future criticism scenario, participants across all three intervention groups (IE, ImRs, ImRs-DSR) reported significant reductions in negative emotions during the anticipation phase. Repeated-measures ANOVAs revealed robust main effects of time for most emotions, with no significant main effects of condition or time × condition interactions, indicating comparable improvements across interventions.

**Sadness.** Ratings showed the strongest decline, F(2.20, 305.60) = 95.74, p < .001, η²_partial = .41. Post hoc tests (Bonferroni corrected) indicated significant decreases from pre-treatment to post-treatment (ΔM = 2.05, p < .001, d = 0.91), 3-month follow-up (ΔM = 2.49, p < .001), and 6-month follow-up (ΔM = 2.51, p < .001), with stability across follow-ups.

**Fear.** A significant time effect emerged, F(2.21, 307.70) = 65.82, p < .001, η²_partial = .32. Reductions were observed from pre-treatment to post-treatment (ΔM = 1.76, p < .001), 3-month follow-up (ΔM = 2.30, p < .001), and 6-month follow-up (ΔM = 2.42, p < .001).

**Guilt.** Ratings also decreased significantly, F(2.18, 303.55) = 41.26, p < .001, η²_partial = .24, with significant reductions from pre-treatment to post-treatment (ΔM = 1.39, p < .001), 3-month follow-up (ΔM = 1.77, p < .001), and 6-month follow-up (ΔM = 1.95, p < .001).

**Anger.** A more modest but significant decline was found, F(2.25, 310.40) = 12.87, p < .001, η²_partial = .09. Reductions were most pronounced from pre-treatment to 6-month follow-up (ΔM = 1.05, p < .001), while earlier pairwise contrasts showed smaller or marginal effects.

**Arousal.** Ratings declined significantly over time, F(2.10, 289.60) = 10.12, p < .001, η²_partial = .07. The only significant post hoc contrast was from pre-treatment to 6-month follow-up (ΔM = 0.69, p = .024, d = 0.32).

**Disgust.** A small but significant time effect was found, F(2.15, 298.80) = 8.41, p < .001, η²_partial = .06. Post hoc tests indicated that only the pre-treatment–post-treatment reduction (ΔM = 0.70, p = .007) was significant, while changes at follow-ups did not reach significance.

These results indicate that anticipation of future criticism elicited reductions in negative emotional responses following intervention. The strongest and most consistent effects were observed for sadness, fear, and guilt, while anger, arousal, and disgust showed more modest changes. Importantly, all effects were consistent across the three interventions and remained stable over time.

In the future criticism scenario, participants across all three intervention groups (IE, ImRs, ImRs-DSR) reported significant reductions in negative emotional responses during the hotspot phase. Repeated-measures ANOVAs revealed robust main effects of time for most emotions, with no significant main effects of condition or time × condition interactions, indicating comparable improvements across interventions.

**Sadness.** Ratings exhibited the most pronounced decline over time, F(2.16, 300.30) = 108.93, p < .001, η²_partial = .44. Post hoc comparisons confirmed highly significant reductions from pre-treatment (pre-treatment) to all subsequent time points: post-treatment (post-treatment; ΔM = 2.17, p < .001, d = 0.97), 3-month follow-up (3-month follow-up; ΔM = 2.60, p < .001), and 6-month follow-up (6-month follow-up; ΔM = 2.64, p < .001). These effects remained stable during follow-up.

**Fear.** Ratings similarly declined, F(2.27, 315.19) = 67.59, p < .001, η²_partial = .33. Reductions from pre-treatment to post-treatment (ΔM = 1.82, p < .001), 3-month follow-up (ΔM = 2.33, p < .001), and 6-month follow-up (ΔM = 2.44, p < .001) were statistically significant and reflected medium-to-large effect sizes.

**Guilt.** Ratings also decreased significantly over time, F(2.20, 304.53) = 40.30, p < .001, η²_partial = .23, with meaningful reductions from pre-treatment to post-treatment (ΔM = 1.41, p < .001), 3-month follow-up (ΔM = 1.82, p < .001), and 6-month follow-up (ΔM = 2.05, p < .001). While changes between post-treatment and later follow-ups were smaller, they remained statistically robust.

**Anger.** A more modest decline was observed, F(2.19, 302.20) = 11.41, p < .001, η²_partial = .08. Significant decreases were found particularly between pre-treatment and 6-month follow-up (ΔM = 1.12, p < .001), though reductions between pre-treatment and post-treatment were only marginally significant after correction (pBonf = .078; pHolm = .039).

**Arousal.** Ratings decreased significantly, F(2.13, 293.50) = 8.96, p < .001, η²_partial = .06, with a significant change from pre-treatment to 6-month follow-up (ΔM = 0.67, p = .026, d = 0.31). Earlier time point comparisons showed smaller or marginal effects.

**Disgust.** Ratings also declined, albeit with smaller effect size, F(2.18, 300.30) = 7.82, p < .001, η²_partial = .05. Only the reduction from pre-treatment to post-treatment (ΔM = 0.73, p = .006) reached significance in post hoc tests, while changes across follow-ups were no longer statistically significant.

These results confirm that participants’ emotional reactivity to imagined future criticism diminished significantly following intervention. Although initial emotional intensity was generally lower than in the untreated autobiographical condition, significant time-related improvements were observed across all affective domains. Crucially, these improvements were consistent across intervention types and remained stable over the follow-up period, supporting the generalizability of therapeutic effects to future-oriented threat scenarios.

### Subjective Measures – Untreated Criticism Scenario (ITT Analysis)

To assess the generalization of therapeutic effects to autobiographical memories that were not directly targeted during the intervention, we analyzed participants’ emotional responses during the anticipation and hotspot phases of the untreated criticism scenario. Repeated-measures ANOVAs with time (pre-treatment, post-treatment, 3-month follow-up, 6-month follow-up) and group (IE, ImRs, ImRs-DSR) as factors revealed significant main effects of time for all examined emotions. However, no significant main effects of group or time × group interactions were observed, indicating comparable trajectories across all intervention conditions.

Anticipation Phase

During the anticipation phase, significant reductions over time were observed across most negative emotional states. Fear showed a particularly strong time effect, F(1.67, 232.35) = 114.94, p < .001, η²_partial = .452, with significant decreases from pre-treatment to post-treatment (ΔM = 1.64, pBonf < .001, d = 1.12), 3-month follow-up (ΔM = 1.85, pBonf < .001), and 6-month follow-up (ΔM = 2.05, pBonf < .001). Sadness followed a similar pattern, F(1.66, 231.21) = 96.67, p < .001, η²_partial = .410, with large and stable decreases across all post-treatment time points (all pBonf < .001).

Guilt also decreased significantly over time, F(1.65, 229.69) = 132.13, p < .001, η²_partial = .487. The reduction between pre-treatment and post-treatment (ΔM = 1.88, pBonf < .001, d = 1.42) remained stable at both follow-ups (3-month follow-up and 6-month follow-up, pBonf < .001).

Anger showed a significant time effect as well, F(1.98, 274.03) = 59.58, p < .001, η²_partial = .300, with reductions from pre-treatment to post-treatment (ΔM = 1.11, pBonf < .001, d = 1.04), 3-month follow-up (ΔM = 1.03, pBonf < .001), and 6-month follow-up (ΔM = 1.06, pBonf < .001), with no further changes between follow-up assessments.

For arousal, the time effect was smaller but still significant, F(1.92, 267.15) = 13.77, p < .001, η²_partial = .091. The decrease from pre-treatment to post-treatment (ΔM = 0.67, pBonf = .009) was statistically significant, though no further differences emerged between follow-up points.

Disgust also decreased significantly, F(2.05, 282.99) = 13.13, p < .001, η²_partial = .087, though effects were smaller and primarily limited to the comparison between pre-treatment and post-treatment.

No significant main effects of group or time × group interactions were observed for any emotion (all ps > .20), indicating that the observed emotional changes occurred independently of intervention type.

Hotspot Phase

A similar pattern emerged during the hotspot phase, with significant and sustained reductions across all negative emotions. Sadness showed the strongest effect of time, F(2.26, 313.59) = 109.21, p < .001, η²_partial = .440, with significant decreases from baseline to post-treatment and both follow-up time points (all pBonf < .001). The largest decrease occurred between pre-treatment and 6-month follow-up (ΔM = 2.64, d = 1.18).

Fear also declined substantially, F(2.17, 301.34) = 37.97, p < .001, η²_partial = .215. Reductions from pre-treatment to post-treatment (ΔM = 1.48, pBonf < .001, d = 0.70), 3-month follow-up (ΔM = 1.48, pBonf < .001), and 6-month follow-up (ΔM = 1.61, pBonf < .001) were all significant and sustained.

Guilt followed the same trajectory, F(2.17, 301.34) = 37.97, p < .001, η²_partial = .215, with stable post-treatment improvements. Anger also declined significantly, F(2.40, 334.51) = 37.97, p < .001, η²_partial = .215, with effects persisting throughout follow-up.

For disgust, a significant time effect was observed, F(2.17, 301.34) = 16.91, p < .001, η²_partial = .108, although changes were less pronounced. Arousal also showed a moderate decline, F(2.17, 301.34) = 16.91, p < .001, η²_partial = .108.

Again, no significant main effects of group or time × group interactions were found (all ps > .20), reinforcing the conclusion that reductions in subjective emotional reactivity occurred independently of intervention condition.

These ITT results demonstrate that participants experienced substantial and sustained reductions in negative emotional responses to autobiographical criticism memories that were not directly targeted during therapy. The strongest effects were observed for sadness and fear, with improvements consistent across both anticipation and hotspot phases. Importantly, emotional attenuation occurred independently of treatment condition, supporting the idea of non-specific generalization of therapeutic gains to related but untreated emotional memories.

### Secondary Outcomes – ITT Analysis

For secondary psychopathology measures (BDI-II, LSAS, PPS, Frost MPS, PTSD Checklist, SGAD, SPANIC, YBOCS), no significant main effects of group or time × group interactions were found (all ps > .10). Several small-to-moderate main effects of time emerged, indicating modest overall symptom reductions across participants.

Depression (BDI-II) showed the clearest effect, F(2.66, 364.82) = 17.01, p < .001, η²_partial = .11, with consistent decreases across all follow-ups. Generalized anxiety (SGAD) also declined robustly, F(1, 137) = 13.76, p < .001, η²_partial = .09, while smaller but significant reductions were observed for worry (SPANIC), PTSD symptoms (PCL-5), and obsessive–compulsive symptoms (YBOCS) (η²_partial ≈ .05). More modest changes were found for social anxiety (LSAS), F(2.33, 319.04) = 3.62, p = .022, η²_partial = .03, and perfectionism (Frost MPS), which showed only trend-level improvement (p = .082). In contrast, Pure Perfectionism Scale (PPS) remained unchanged (p = .902).

Summary. In the ITT sample, interventions were associated with reliable improvements in depression, generalized anxiety, worry, PTSD, and obsessive–compulsive symptoms, with smaller and less consistent effects on social anxiety and perfectionism. Importantly, all improvements were comparable across groups, with no evidence for differential intervention effects.

## Per Protocol analyses

### Short- & Long-term Treatment Effects - Treated Criticism Scenario SCL Response (analyses per protocol)

Physiological responses during the anticipation of criticism were assessed by analyzing skin conductance level (SCL) across four time points (pre-treatment, post-treatment, 3-month follow-up, 6-month follow-up) within three experimental procedures: recall, renewal, and reinstatement. Repeated-measures ANOVAs with the factors Group (IE, ImRs, ImRs-DSR) and Time (4 levels) were conducted separately for each procedure (analyses per protocol).

In the recall procedure, SCL during anticipation showed a robust and significant decrease over time, F(2.50, 257.52) = 49.87, p < .001, η²p = .33, reflecting a substantial reduction in physiological arousal following the intervention. A significant Time × Group interaction was also observed, F(5.00, 257.52) = 2.99, p = .012, η²p = .055, indicating that the magnitude of reduction varied somewhat between groups. However, post-hoc tests showed that all groups achieved significant and maintained reductions from pre-treatment to all subsequent time points (post-treatment, 3-month, 6-month follow-ups), with no consistent superiority of any one group across all comparisons.

In the renewal procedure, anticipatory SCL also declined significantly over time, F(2.34, 229.74) = 91.25, p < .001, η²p = .48. The interaction between Time and Group was not significant, F(4.69, 229.74) = 1.77, p = .124, η²p = .035, suggesting comparable reductions across intervention groups. Descriptive trends indicated the ImRs-DSR group had numerically greater baseline reductions compared to the IE and ImRs groups, although these differences were not statistically significant.

For the reinstatement procedure, anticipatory SCL decreased significantly as well, F(2.13, 168.11) = 64.22, p < .001, η²p = .45. The Time × Group interaction approached significance, F(4.26, 168.11) = 1.95, p = .101, η²p = .047, but post-hoc comparisons revealed no consistent significant differences between groups. Reductions in physiological arousal remained stable across the follow-up period in all groups.

Additionally, physiological reactivity during the hotspot phase—representing peak emotional activation—within the reinstatement procedure showed a significant main effect of time, F(2.54, 192.84) = 6.76, p < .001, η²p = .08, indicating reduced autonomic arousal across sessions. The Time × Group interaction was not significant, F(5.08, 192.84) = 0.94, p = .454, η²p = .024. Descriptively, all groups showed a decline in SCL reactivity during the hotspot. The ImRs-DSR group exhibited the largest absolute reduction from baseline, but these group differences did not reach statistical significance, and the observed trends remained stable across follow-up assessments.

### Salivary Alpha-Amylase (sAA) (Per Protocol analysis)

Analyses of salivary alpha-amylase revealed a small but significant main effect of time, F(1, 123) = 4.61, p = .034, η²_partial = .036. Post hoc comparisons indicated a modest reduction in sAA from pre-treatment (pre-treatment) to post-treatment (post-treatment; ΔM = 8.65, SE = 4.03, t = 2.15, pBonf = .034, d = 0.10). However, no significant group or time × group interaction effects were observed (all ps > .13), suggesting that this change was consistent across interventions.

### Subjective response during anticipation of the treated criticism scenario (Per Protocol analysis)

A 3 (group: IE vs. ImRs vs. ImRs-DSR) × 4 (time point: pre-treatment [pre-treatment], post-treatment [post-treatment], 3-month follow-up [3-month follow-up], and 6-month follow-up [6-month follow-up]) repeated-measures ANOVA was conducted for each self-reported emotion assessed during the anticipation phase of the treated criticism scenario.

The analyses revealed significant main effects of time for all negative emotional responses, indicating a consistent reduction in emotional reactivity from pre- to post-treatment and across follow-up time points, independent of group allocation. For sadness, the main effect of time was significant, F(1.72, 150.02) = 65.21, p < .001, η²_partial = .43. Post hoc comparisons showed significant decreases from pre-treatment (pre-treatment) to all subsequent time points: post-treatment (post-treatment; ΔM = 1.76, SE = 0.19, t = 9.52, pBonf < .001, d = 1.25), 3-month follow-up (3-month follow-up; ΔM = 1.84, SE = 0.20, t = 9.24, pBonf < .001), and 6-month follow-up (6-month follow-up; ΔM = 1.74, SE = 0.21, t = 8.41, pBonf < .001). No significant differences were observed between the three follow-up time points.

A similar pattern emerged for fear, where a robust main effect of time was observed, F(2.05, 177.95) = 50.14, p < .001, η²_partial = .20. Fear ratings significantly declined from pre-treatment to post-treatment (ΔM = 1.76, SE = 0.21, t = 8.47, pBonf < .001), 3-month follow-up (ΔM = 1.89, SE = 0.21, t = 8.95, pBonf < .001), and 6-month follow-up (ΔM = 1.81, SE = 0.24, t = 7.57, pBonf < .001). Likewise, guilt ratings also showed a significant main effect of time, F(1.76, 153.12) = 39.51, p < .001, η²_partial = .31, with post hoc tests revealing significant reductions at all post-treatment time points relative to baseline. Disgust followed a similar trajectory, F(2.15, 187.42) = 15.84, p < .001, η²_partial = .15, with all pairwise comparisons to pre-treatment reaching significance.

For arousal, a significant time effect was again observed, F(2.08, 180.64) = 21.85, p < .001, η²_partial = .20. Arousal ratings dropped significantly from pre-treatment to post-treatment (ΔM = 1.08, SE = 0.25, t = 4.42, pBonf < .001), 3-month follow-up (ΔM = 1.56, SE = 0.26, t = 5.90, pBonf < .001), and 6-month follow-up (ΔM = 1.42, SE = 0.27, t = 5.37, pBonf < .001). Finally, anger ratings also decreased over time, F(2.41, 139.76) = 13.19, p < .001, η²_partial = .19, with significant reductions from baseline to each follow-up.

No significant main effects of group were observed for any emotion (all ps > .4), nor were there any significant time × group interaction effects (all ps > .05). These results suggest that all three intervention conditions led to comparable and sustained reductions in negative affect during the anticipation of the critical memory, and that these improvements were maintained at 3- and 6-month follow-ups.

### Emotional Reactivity During Hotspot (Per Protocol Analysis)

To assess emotional reactivity during the most emotionally intense part of the criticism scenario (hotspot), we conducted repeated-measures ANOVAs with 3 (condition: IE, ImRs, ImRs-DSR) × 4 (time point: pre-treatment [pre-treatment], post-treatment [post-treatment], 3-month follow-up [3-month follow-up], 6-month follow-up [6-month follow-up]) factors for self-reported emotions.

**Anger** showed a significant main effect of time, *F*(2.09, 181.62) = 20.11, *p* < .001, η²_partial = .188. Post hoc comparisons (Bonferroni corrected) indicated a significant reduction in anger from pre-treatment to post-treatment (ΔM = 1.56, *SE* = 0.27, *t* = 5.82, *p*Bonf < .001, *d* = 0.69), 3-month follow-up (ΔM = 1.54, *SE* = 0.32, *t* = 4.89, *p*Bonf < .001), and 6-month follow-up (ΔM = 1.70, *SE* = 0.32, *t* = 5.31, *p*Bonf < .001). No significant main effects of condition or condition × time interactions were found.

**Fear** also showed a robust time effect, *F*(2.35, 204.31) = 52.55, *p* < .001, η²_partial = .377. Fear significantly decreased from pre-treatment to post-treatment (ΔM = 2.23, *SE* = 0.26, *t* = 8.72, *p*Bonf < .001, *d* = 0.94), 3-month follow-up (ΔM = 2.27, *SE* = 0.28, *t* = 8.07, *p*Bonf < .001), and 6-month follow-up (ΔM = 2.63, *SE* = 0.28, *t* = 9.55, *p*Bonf < .001). Again, no significant differences emerged between conditions or in interaction terms.

Sadness ratings showed a similarly strong time effect, *F*(2.46, 214.32) = 64.56, *p* < .001, η²_partial = .426. Pairwise comparisons indicated significant decreases in sadness from pre-treatment to post-treatment (ΔM = 2.48, *SE* = 0.25, *t* = 10.02, *p*Bonf < .001, *d* = 1.10), 3-month follow-up (ΔM = 2.47, *SE* = 0.27, *t* = 9.00, *p*Bonf < .001), and 6-month follow-up (ΔM = 2.92, *SE* = 0.28, *t* = 10.49, *p*Bonf < .001). No group or interaction effects were significant.

For guilt, a significant effect of time was observed, *F*(2.36, 205.69) = 48.03, *p* < .001, η²_partial = .356. Guilt dropped significantly from pre-treatment to post-treatment (ΔM = 2.46, *SE* = 0.25, *t* = 9.76, *p*Bonf < .001), 3-month follow-up (ΔM = 2.20, *SE* = 0.30, *t* = 7.34, *p*Bonf < .001), and 6-month follow-up (ΔM = 2.58, *SE* = 0.29, *t* = 8.82, *p*Bonf < .001). No significant condition or interaction effects were found.

A significant time effect was also present for arousal, *F*(2.11, 183.87) = 13.94, *p* < .001, η²_partial = .138. Arousal decreased from pre-treatment to post-treatment (ΔM = 0.87, *SE* = 0.28, *t* = 3.10, *p*Bonf = .016), 3-month follow-up (ΔM = 1.31, *SE* = 0.29, *t* = 4.52, *p*Bonf < .001), and 6-month follow-up (ΔM = 1.40, *SE* = 0.31, *t* = 4.59, *p*Bonf < .001). Some minor group × time trends were visible but did not reach statistical significance.

Disgust followed a similar pattern with a significant time effect, *F*(2.40, 209.16) = 16.48, *p* < .001, η²_partial = .159. Post hoc tests showed significant reductions from pre-treatment to post-treatment (ΔM = 1.33, *SE* = 0.23, *t* = 5.81, *p*Bonf < .001), 3-month follow-up (ΔM = 1.06, *SE* = 0.27, *t* = 3.93, *p*Bonf = .001), and 6-month follow-up (ΔM = 1.38, *SE* = 0.26, *t* = 5.24, *p*Bonf < .001).

No significant main effects of condition or time × condition interactions were observed for any emotion, suggesting that all groups benefited comparably in terms of reduced subjective emotional reactivity during the hotspot phase.

### PFAI (Performance Failure Appraisal Inventory, Per Protocol analysis)

For fear of failure, a significant main effect of time was found, F(2.43, 209.25) = 9.72, p < .001, η²_partial = .102. Post hoc analyses showed significant decreases from pre-treatment (pre-treatment) to post-treatment (post-treatment; ΔM = 9.85, SE = 2.56, t = 3.85, pBonf = .001, d = 0.47), 3-month follow-up (3-month follow-up; ΔM = 8.09, SE = 2.32, t = 3.48, pBonf = .005), and 6-month follow-up (6-month follow-up; ΔM = 11.48, SE = 2.58, t = 4.44, pBonf < .001, d = 0.55). No significant time × group interaction or between-group differences were observed (all ps > .29), indicating comparable improvements across conditions.

### Generalizability SCL Response– Untreated & Future criticism scenarios (Per protocol analysis)

To test the generalization of treatment effects, we conducted separate 3 (condition: IE, ImRs, ImRs-DSR) × 4 (time point: pre-treatment [pre-treatment], post-treatment [post-treatment], 3-month follow-up [3-month follow-up], and 6-month follow-up [6-month follow-up]) repeated-measures ANOVAs on skin conductance level (SCL) for the untreated and future criticism scenarios, across anticipation and hotspot phases.

For the untreated criticism scenario (anticipation phase), no significant main effect of time was found in the recall (F(2.82, 290.25) = 3.08, p = .031, η²_partial = .029), renewal (F(2.92, 286.00) = 0.87, p = .455, η²_partial = .009), or reinstatement procedures (F(2.85, 236.74) = 2.03, p = .114, η²_partial = .024). A post hoc comparison revealed a significant reduction in SCL from pre-treatment to post-treatment during the recall procedure (ΔM = –3.91, SE = 1.41, t = –2.77, pBonf = .039, d = 0.33). No other comparisons reached significance. There were no significant main effects of condition or time × condition interactions in any procedure.

For the future criticism scenario, strong time effects emerged in both anticipation and hotspot phases. In the anticipation phase, a robust main effect of time was observed, F(1.36, 115.15) = 27.75, p < .001, η²_partial = .246. Post hoc tests (Bonferroni corrected) showed significant increases in SCL from pre-treatment to post-treatment (ΔM = 9.61, SE = 1.54, t = 6.26, pBonf < .001), 3-month follow-up (ΔM = 7.60, SE = 1.54, t = 4.92, pBonf < .001), and 6-month follow-up (ΔM = 7.57, SE = 1.47, t = 5.16, pBonf < .001). SCL remained elevated at follow-ups compared to baseline. No significant condition or interaction effects were found.

In the hotspot phase, a similar pattern was found with a significant main effect of time, F(1.34, 113.80) = 28.65, p < .001, η²_partial = .252. Post hoc comparisons indicated SCL increased significantly from pre-treatment to post-treatment (ΔM = 9.66, SE = 1.52, t = 6.38, pBonf < .001), 3-month follow-up (ΔM = 7.59, SE = 1.53, t = 4.96, pBonf < .001), and 6-month follow-up (ΔM = 7.67, SE = 1.47, t = 5.21, pBonf < .001). Again, no main effects of condition or interaction effects were observed.

These results suggest that while physiological reactivity to untreated criticism remained mostly stable, SCL responses to future criticism scenarios increased significantly after the intervention and remained elevated at follow-up. This increase may reflect heightened salience or novelty of future-oriented threat imagery not directly targeted by treatment, and it was comparable across all intervention variants.

### Subjective measures Untreated & Future criticism scenarios (Per Protocol analysis)

In the future criticism scenario, participants in all three conditions (IE, ImRs, ImRs-DSR) reported significant reductions in subjective emotional intensity over time during the hotspot phase. Significant main effects of time were found for sadness, F(2.26, 192.90) = 50.32, p < .001, η²_partial = .372, with Bonferroni-corrected comparisons confirming large and sustained decreases from pre-treatment (pre-treatment) to post-treatment (pBonf < .001, d = 0.86), 3-month follow-up (pBonf < .001), and 6-month follow-up (pBonf < .001). Similar robust effects were observed for fear, F(2.33, 198.86) = 21.13, p < .001, η²_partial = .199, and guilt, F(2.45, 209.52) = 27.62, p < .001, η²_partial = .245, both showing significant reductions from pre-treatment to all post-treatment time points (psBonf < .001).

Anger also decreased significantly, F(2.52, 213.56) = 9.40, p < .001, η²_partial = .100, though with slightly smaller effect sizes. For instance, a significant reduction was found between pre-treatment and 6-month follow-up (pBonf < .001, d = 0.58), while intermediate differences were smaller or non-significant. Additionally, subjective arousal decreased over time, F(2.38, 202.22) = 7.26, p < .001, η²_partial = .079, with a significant drop between pre-treatment and 6-month follow-up (pBonf = .021). Disgust ratings showed a more modest reduction, F(2.63, 224.50) = 5.61, p = .002, η²_partial = .062, with a significant decrease only from pre-treatment to post-treatment or 3-month follow-up (psBonf < .05), but not between post-treatment and follow-up sessions.

No significant condition effects or time × condition interactions were observed for any emotion (all ps > .05), indicating comparable reductions across all three intervention types.

These results suggest that, although initial emotional intensity in the future criticism scenario was generally lower compared to the untreated autobiographical scenario, participants still showed meaningful reductions in negative emotional responses and physiological arousal over time. Importantly, these effects were not specific to any intervention condition and remained stable during the follow-up period.

### Secondary Outcomes – Per Protocol

Analyses of secondary psychopathology measures (LSAS, PPS, Frost MPS, BDI-II, PTSD Checklist, and additional anxiety-related indices) revealed no significant main effects of group or time × group interactions (all ps > .17). Several small-to-moderate main effects of time emerged, indicating modest overall symptom reductions across participants.

Depression (BDI-II) showed the clearest pattern, F(2.79, 220.38) = 10.17, p < .001, η²_partial = .11, with consistent decreases from baseline to all follow-ups. PTSD symptoms (PCL-5) also declined significantly over time, F(1, 75) = 6.96, p = .010, η²_partial = .09, mainly from pre- to post-treatment. Additional small but significant reductions were observed for social anxiety (LSAS), F(2.37, 272.30) = 2.90, p = .048, η²_partial = .03, and perfectionism (Frost MPS), F(2.61, 281.72) = 2.77, p = .050, η²_partial = .03. Furthermore, generalized anxiety (SGAD: F(1, 75) = 14.34, p < .001, η²_partial = .16), worry (SPANIC: F(1, 75) = 6.76, p = .011, η²_partial = .08), and obsessive–compulsive symptoms (YBOCS: F(1, 75) = 6.29, p = .014, η²_partial = .08) showed significant decreases from pre- to post-treatment. By contrast, PPS scores did not change significantly (ps > .48).

Concluding, in the per protocol sample, interventions were associated with modest but reliable improvements in depressive symptoms, generalized anxiety, fear- and worry-related measures, and selected aspects of social anxiety and perfectionism. Importantly, these effects were comparable across intervention groups, and no differential treatment effects emerged.

## Questions regarding therapy attendance

| ORIGINAL (PL) | TRANSLATION (ENG) |
| --- | --- |
| Czy obecnie bierze Pan/Pani udział w zajęciach grupowych (treningi interpersonalne, grupy wsparcia, kursy w dziedzinie samorozwoju) ?  Tak (1)  Nie (2)  Trudno powiedzieć (3) | Do you currently participate in group activities (interpersonal training, support groups, self-development courses) ?  Yes (1)  No (2)  Hard to say (3) |
| Jakie? | Which ones? |
| Czy uczęszcza Pan/Pani na psychoterapię?  Tak (1)  Nie (2) | Do you attend psychotherapy?  Yes (1)  No (2) |
| Czy uczęszczał/a Pan/Pani na psychoterapię w przeszłości?  Tak (1)  Nie (2) | Have you attended psychotherapy in the past?  Yes (1)  No (2) |
| Ile trwała terapia? | How long did the therapy last? |
| Kiedy się skończyła? | When did it end? |
| W jakim była nurcie? | What was the type of therapy? |
| TiOT7 Czy planował Pan/Pani rozpocząć psychoterapię lub kursy samorozwojowe w najbliższym czasie?  Tak (1)  Nie (2) | Did you plan to start psychotherapy or self-development courses in the near future?  Yes (1)  No (2) |
| Czy jest Pan/Pani gotowy/a odroczyć takie decyzje do zakończenia projektu (okres 7 miesięcy)?  Tak (1)  Nie (2)  Trudno powiedzieć (3) | Are you willing to postpone such decisions until the end of the project (7-month period)?  Yes (1)  No (2)  Hard to say (3) |
| Czy przyjmuje Pan/Pani stale leki, mające wpływ na stan psychiczny (neurologiczne, psychiatryczne, endokrynologiczne itp.)?  Tak (1)  Nie (2)  Trudno powiedzieć (3) | Are you constantly taking medications that affect your mental state (neurological, psychiatric, endocrine, etc.)?  Yes (1)  No (2)  Hard to say (3) |

## Verification of punitive parental experiences

| ORIGINAL (PL) | TRANSLATION (ENG) |
| --- | --- |
| Czy w dzieciństwie doświadczył/a Pan/Pani: | Have you experienced the following in your childhood: |
| ...przemocy fizycznej ze strony:  rodzica/opiekuna  Tak (1) Nie (2)  rodziców/opiekunów  Tak (1) Nie (2)  innych osób dorosłych  Tak (1) Nie (2)  rówieśników Tak (1) Nie (2) | ...physical violence by:  parent/guardian Yes (1) No (2)  parents/guardiansYes (1) No (2)  other adults Yes (1) No (2)  peers Yes (1) No (2) |
| Jaka to była przemoc? | What kind of violence was it? |
| Czy to było wydarzenie jednorazowe czy trwało przez jakiś czas?  Jednorazowe (1)  Trwało pewien czas (2) | Was it a one-time event or did it go on for some time?  One-time (1)  Lasted for some time (2) |
| Jak długo? | For how long? |
| ...poniżania, agresji słowna, wyszydzania, wyśmiewania, wykluczenia ze strony:  Rodzica/opiekuna Yes (1) No (2)  Rodziców/opiekunów Yes (1) No (2)  Innych osób dorosłych Yes (1) No (2)  Rówieśników Yes (1) No (2) | ...humiliation, verbal aggression, mocking, ridicule, exclusion from the side of:  Parent/guardian Yes (1) No (2)  Parents/guardians Yes (1) No (2)  Other adults Yes (1) No (2)  Peers Yes (1) No (2) |
| Czy to było wydarzenie jednorazowe czy trwało przez jakiś czas?  Jednorazowe (1)  Trwało pewien czas (2) | Was this a one-time event or did it last for a while?  One-time (1)  Lasted for some time (2) |
| Jak długo? | How long? |
| ...wykorzystywania seksualnego przez rodzica/ opiekuna, rodziców/opiekunów? | ...sexual abuse by parent/guardian, parents/guardians? |
| Ze strony kogo? | From whose side? |
| ...naruszenia własnych granic a aspekcie seksualnym( gwałt, molestowanie, niechciany dotyk, nadmierne wnoszenie tematu seksu w dzieciństwie bądź bycie świadkiem takiej sytuacji)?  Tak (1)  Nie (2) | ...violation of your boundaries in a sexual aspect (rape, molestation, unwanted touching, excessive thematizing of the topic of sex in childhood) or being a witness to such a situation?  Yes (1)  No (2) |

## References

Babor, T. F., Fuente, J. R., Saunders, J., & Grant, M. (1998). Test Rozpoznawania Zaburzeń Związanych z Piciem Alkoholu AUDIT, tł. Mira Prajsner. *Warszawa, Wyd. WHO/PARPA, 36.*

Beck, A. T., Steer, R. A., & Brown, G. K. (1987). Beck depression inventory. *New York: Harcourt Brace Jovanovich.*

Conroy, D. E., Willow, J. P., & Metzler, J. N. (2002). Multidimensional fear of failure measurement: The performance failure appraisal inventory. *Journal of applied sport psychology, 14(2), 76-90.*

Craske M, Wittchen U, Bogels S, Stein M, Andrews G, Lebeu R. (2013a) Severity Measure for Agoraphobia—Adult, *American Psychiatric Association.*

Craske M, Wittchen U, Bogels S, Stein M, Andrews G, Lebeu R. (2013b) Severity Measure for Generalized Anxiety Disorder—Adult, *American Psychiatric Association.*

Craske M, Wittchen U, Bogels S, Stein M, Andrews G, Lebeu R. (2013c) *S*everity Measure for Panic Disorder—Adult, *American Psychiatric Association.*

Craske M, Wittchen U, Bogels S, Stein M, Andrews G, Lebeu R. (2013d) Severity of Posttraumatic Stress Symptoms—Adult, *American Psychiatric Association.*

First, M. B., Williams, J. B., Karg, R. S., & Spitzer, R. L. (2016). User's guide for the SCID-5-CV Structured Clinical Interview for DSM-5® disorders: Clinical version. *American Psychiatric Publishing, Inc..*

Golińska, L. (2017). Polska adaptacja Inwentarza do Oceny Porażki (The Performance Failure Appraisal Inventory–PFAI) Davida Conroya. *Pedagogika Rodziny, 7(3), 89-105.*

Goodman, W. K., Price, L. H., Rasmussen, S. A., Mazure, C., Delgado, P., Heninger, G. R., & Charney, D. S. (1989). The yale-brown obsessive compulsive scale: II. Validity. Archives of general psychiatry, 46(11), 1012-1016.

Heimberg, R. G., Horner, K. J., Juster, H. R., Safren, S. A., Brown, E. J., Schneier, F. R., & Liebowitz, M. R. (1999). Psychometric properties of the Liebowitz social anxiety scale. *Psychological medicine, 29(1), 199-212.*

Łojek, E., & Stańczak, J. (2019). Inwentarz Depresji Becka–Drugie Wydanie [Beck Depression Inventory–Second Edition; BDI-II]. *Warszawa: Pracownia Testów Psychologicznych.*

Masiak, M., & Przychoda, J. M. I. N. I. (1998). MINI Mini international neuropsychiatric interview. Polish version 5.0. 0. *Katedra i Klinika Psychiatrii Akademii Medycznej, Lublin*

Piotrowski, K., & Bojanowska, A. (2021). Factor structure and psychometric properties of a Polish adaptation of the Frost Multidimensional Perfectionism Scale. *Current Psychology, 40(6), 2754-2763.*

Saunders, J. B., Aasland, O. G., Babor, T. F., De la Fuente, J. R., & Grant, M. (1993). Development of the alcohol use disorders identification test (AUDIT): WHO collaborative project on early detection of persons with harmful alcohol consumption‐II. *Addiction, 88(6), 791-804.*

Sheehan, D. V., Lecrubier, Y., Sheehan, K. H., Amorim, P., Janavs, J., Weiller, E., ... & Dunbar, G. C. (1998). The Mini-International Neuropsychiatric Interview (MINI): the development and validation of a structured diagnostic psychiatric interview for DSM-IV and ICD-10. *Journal of clinical psychiatry, 59(20), 22-33.*

Skinner, H. A. (1982). The drug abuse screening test. *Addictive behaviors, 7(4), 363-371.*

Steel P (2010) Arousal, avoidant and decisional procrastinators: Do they exist? *Personality and Individual Differences 48: 926–934.*

Stępień, M., & Topolewska, E. (2014). Style tożsamości w ujęciu Berzonsky’ego a prokrastynacja, [w:] E. Topolewska, E. Skimina, S. Skrzek (red.), *Młoda Psychologia, 2.*

Stöber, J. (1998). The Frost Multidimensional Perfectionism Scale revisited: More perfect with four (instead of six) dimensions. *Personality and individual differences, 24(4), 481-491.*

Zawadzki, B. (2017). Lokalizacja zaburzeń osobowości w Kołowym Modelu Metacech Osobowości. *Roczniki Psychologiczne, 20(2), 473-4*

|  | **Time** | | | **Group** | | | **Time x Group** | | |
| --- | --- | --- | --- | --- | --- | --- | --- | --- | --- |
| **Analysis** | **F** | **p** | **Effect Size (η²p)** | **F** | **p** | **Effect Size (η²p)** | **F** | **p** | **Effect Size (η²p)** |
| SCL Anticipation Treated Criticism Recall | 49.870 (2.500,257.517) | < .001 | .326 | .742 (2,103) | .479 | .014 | 2.990 (5.000,257.517) | .012 | .055 |
| SCL Anticipation Treated Criticism Reinstatement | 64.219 (2.128,168.110) | < .001 | .448 | 3.207 (2,79) | .046 | .075 | 1.945 (4.256,168.110) | .101 | .047 |
| SCL Anticipation Treated Criticism Renewal | 91.250 (2.344,229.741) | < .001 | .482 | 2.338 (2,98) | .102 | .046 | 1.774 (4.689,229.741) | .124 | .035 |
| SCL Hotspot Treated Criticism Reinstatement | 6.762 (2.537,192.836) | < .001 | .082 | 1.764 (2,76) | .178 | .044 | .944 (5.075,192.836) | .454 | .024 |
| SCL Anticipation Untreated Criticism Recall | 3.084 (2.818,290.253) | .031 | .029 | .270 (2,103) | .764 | .005 | .733 (5.636,290.253) | .615 | .014 |
| SCL Anticipation Untreated Criticism Reinstatement | 2.030 (2.852,236.735) | .114 | .024 | 1.224 (2,83) | .299 | .029 | .559 (5.704,236.735) | .754 | .013 |
| SCL Anticipation Untreated Criticism Renewal | .868 (2.918,286.000) | .455 | .009 | 1.396 (2,98) | .253 | .028 | .470 (5.837,286.000) | .826 | .009 |
| SCL Anticipation Future Criticism | 35.541 (1.488,184.558) | < .001 | 0.223 | 2.155 (2,124) | 0.120 | 0.034 | 0.783 (2.977,184.558) | 0.504 | 0.012 |
| SCL Hotspot Future Criticism | 28.652 (1.339,113.802) | < .001 | .252 | 2.367 (2,85) | .100 | .053 | 1.962 (2.678,113.802) | .130 | .044 |
| Rating of Treated Criticism Anticipation Arousal | 21.846 (2.076,180.643) | < .001 | .201 | .103 (2,87) | .902 | .002 | .625 (4.153,180.643) | .651 | .014 |
| Rating of Treated Criticism Anticipation Disgust | 15.841 (2.154,187.418) | < .001 | .154 | .462 (2,87) | .631 | .011 | .888 (4.308,187.418) | .478 | .02 |
| Rating of Treated Criticism Anticipation Fear | 50.142 (2.045,177.950) | < .001 | .195 | .248 (2,87) | .781 | .003 | .614 (4.091,177.950) | .657 | .005 |
| Rating of Treated Criticism Anticipation Guilt | 39.509 (1.760,153.117) | < .001 | .312 | .853 (2,87) | .430 | .019 | .569 (3.520,153.117) | .664 | .013 |
| Rating of Treated Criticism Anticipation Sadness | 65.205 (1.724,150.023) | < .001 | .428 | .480 (2,87) | .620 | .011 | 1.581 (3.449,150.023) | .190 | .035 |
| Rating of Treated Criticism Reactivation Anger | 23.979 (2.167,188.566) | < .001 | .216 | .692 (2,87) | .503 | .016 | .542 (4.335,188.566) | .720 | .012 |
| Rating of Treated Criticism Hotspot Anger | 20.108 (2.088,181.619) | < .001 | .188 | .059 (2,87) | .942 | .001 | .452 (4.175,181.619) | .779 | .01 |
| Rating of Treated Criticism Hotspot Arousal | 13.935 (2.113,183.865) | < .001 | .138 | .641 (2,87) | .529 | .015 | 1.614 (4.227,183.865) | .169 | .036 |
| Rating of Treated Criticism Hotspot Disgust | 16.483 (2.404,209.164) | < .001 | .159 | .580 (2,87) | .562 | .013 | .496 (4.808,209.164) | .772 | .011 |
| Rating of Treated Criticism Hotspot Fear | 52.551 (2.348,204.308) | < .001 | .377 | .180 (2,87) | .836 | .004 | .338 (4.697,204.308) | .880 | .008 |
| Rating of Treated Criticism Hotspot Guilt | 48.034 (2.364,205.694) | < .001 | .356 | .565 (2,87) | .570 | .013 | .168 (4.729,205.694) | .970 | .004 |
| Rating of Treated Criticism Hotspot Sadness | 64.556 (2.463,214.324) | < .001 | .426 | .080 (2,87) | .923 | .002 | .463 (4.927,214.324) | .801 | .011 |
| Rating of Future Anticipation Anger | 93.676 (1.672,143.834) | < .001 | .521 | 1.453 (2,86) | .240 | .033 | .632 (3.345,143.834) | .612 | .014 |
| Rating of Future Anticipation Arousal | 29.969 (2.355,202.552) | < .001 | .258 | 1.240 (2,86) | .295 | .028 | .576 (4.711,202.552) | .708 | .013 |
| Rating of Future Anticipation Disgust | 45.968 (1.728,148.607) | < .001 | .348 | 3.120 (2,86) | .049 | .068 | .341 (3.456,148.607) | .823 | .008 |
| Rating of Future Anticipation Fear | 67.614 (1.902,163.614) | < .001 | .44 | .298 (2,86) | .743 | .007 | .544 (3.805,163.614) | .695 | .012 |
| Rating of Future Anticipation Guilt | 178.200 (1.956,168.215) | < .001 | .674 | .128 (2,86) | .880 | .003 | 2.130 (3.912,168.215) | .081 | .047 |
| Rating of Future Anticipation Sadness | 268.198 (1.989,171.046) | < .001 | .757 | 1.414 (2,86) | .249 | .032 | 1.272 (3.978,171.046) | .283 | .029 |
| Rating of Future Criticism Hotspot Anger | 9.400 (2.517,213.972) | < .001 | .1 | .604 (2,85) | .549 | .014 | .882 (5.035,213.972) | .495 | .02 |
| Rating of Future Hotspot Arousal | 7.262 (2.382,202.471) | < .001 | .079 | .479 (2,85) | .621 | .011 | .855 (4.764,202.471) | .508 | .02 |
| Rating of Future Hotspot Disgust | 5.614 (2.630,223.587) | .002 | .062 | 2.840 (2,85) | .064 | .063 | .195 (5.261,223.587) | .969 | .005 |
| Rating of Future Hotspot Fear | 21.128 (2.328,197.888) | < .001 | .199 | .360 (2,85) | .699 | .008 | 1.220 (4.656,197.888) | .302 | .028 |
| Rating of Future Hotspot Guilt | 27.623 (2.446,207.878) | < .001 | .245 | .602 (2,85) | .550 | .014 | .814 (4.891,207.878) | .539 | .019 |
| Rating of Future Hotspot Sadness | 50.324 (2.262,192.266) | < .001 | .372 | .351 (2,85) | .705 | .008 | .533 (4.524,192.266) | .734 | .012 |
| Rating of Untreated Anticipation Anger | 41.544 (2.131,185.406) | < .001 | .323 | .528 (2,87) | .592 | .012 | 1.485 (4.262,185.406) | .205 | .033 |
| Rating of Untreated Anticipation Arousal | 34.132 (2.145,186.656) | < .001 | .282 | .253 (2,87) | .777 | .006 | .627 (4.291,186.656) | .655 | .014 |
| Rating of Untreated Anticipation Disgust | 21.992 (2.254,196.094) | < .001 | .202 | 1.132 (2,87) | .327 | .025 | .761 (4.508,196.094) | .566 | .017 |
| Rating of Untreated Anticipation Fear | 68.533 (1.877,163.333) | < .001 | .441 | .358 (2,87) | .700 | .008 | .331 (3.755,163.333) | .846 | .008 |
| Rating of Untreated Anticipation Guilt | 75.532 (1.800,156.567) | < .001 | .465 | .875 (2,87) | .420 | .02 | .587 (3.599,156.567) | .655 | .013 |
| Rating of Untreated Anticipation Sadness | 104.451 (1.816,157.962) | < .001 | .546 | 1.424 (2,87) | .246 | .032 | .914 (3.631,157.962) | .450 | .021 |
| Rating of Untreated Hotspot Anger | 28.604 (2.532,220.295) | < .001 | .247 | .073 (2,87) | .929 | .002 | .668 (5.064,220.295) | .650 | .015 |
| Rating of Untreated Hotspot Arousal | 15.161 (2.216,192.750) | < .001 | .148 | .063 (2,87) | .939 | .001 | .682 (4.431,192.750) | .620 | .015 |
| Rating of Untreated Hotspot Disgust | 8.935 (2.393,208.190) | < .001 | .093 | 1.898 (2,87) | .156 | .042 | 1.010 (4.786,208.190) | .411 | .023 |
| Rating of Untreated Hotspot Fear | 39.010 (2.144,186.534) | < .001 | .31 | .085 (2,87) | .918 | .002 | .273 (4.288,186.534) | .906 | .006 |
| Rating of Untreated Hotspot Guilt | 50.209 (2.206,191.892) | < .001 | .366 | .649 (2,87) | .525 | .015 | .266 (4.411,191.892) | .914 | .006 |
| Rating of Untreated Hotspot Sadness | 65.040 (2.440,212.308) | < .001 | .428 | .251 (2,87) | .779 | .006 | .536 (4.881,212.308) | .745 | .012 |

### Supplementary Table 1. Per Protocol Analysis

| **Analysis** | **pre-treatment** | | | **post-treatment** | | | **3-month follow-up** | | | **6-month follow-up** | | |
| --- | --- | --- | --- | --- | --- | --- | --- | --- | --- | --- | --- | --- |
|  | **IE** | **ImRs** | **ImRs-DSR** | **IE** | **ImRs** | **ImRs-DSR** | **IE** | **ImRs** | **ImRs-DSR** | **IE** | **ImRs** | **ImRs-DSR** |
| SCL Anticipation Treated Criticism Recall | 1.719 (12.830) | 6.264 (15.616) | 11.722 (17.991) | -8.536 (9.970) | -10.651 (9.042) | -11.303 (10.897) | -7.963 (10.613) | -4.875 (9.478) | -11.013 (12.457) | -10.241 (10.027) | -8.423 (9.782) | -8.687 (11.187) |
| SCL Anticipation Treated Criticism Reinstatement | 2.431 (13.297) | 9.628 (15.884) | 13.582 (19.261) | -11.191 (9.451) | -12.217 (8.734) | -7.287 (9.361) | -12.901 (10.001) | -14.730 (10.451) | -12.555 (11.327) | -10.503 (9.519) | -15.292 (8.911) | -12.097 (10.116) |
| SCL Anticipation Treated Criticism Renewal | 2.074 (12.661) | 6.343 (15.701) | 13.581 (18.189) | -15.477 (11.972) | -14.036 (11.367) | -13.555 (10.407) | -15.463 (10.100) | -14.946 (11.434) | -15.138 (10.168) | -13.883 (11.377) | -15.051 (11.421) | -12.417 (8.881) |
| SCL Hotspot Treated Criticism Reinstatement | 0.209 (16.740) | 6.981 (25.633) | 14.183 (24.269) | -6.543 (16.861) | -9.168 (15.771) | -0.259 (17.939) | -4.413 (14.106) | -7.497 (19.256) | -6.246 (17.513) | -1.649 (29.332) | -7.983 (23.897) | -2.065 (31.276) |
| SCL Anticipation Future Criticism | -1.101 (6.986) | 0.690 (10.986) | 2.804 (11.755) | 7.511 (4.848) | 7.783 (5.472) | 8.595 (5.153) | 6.829 (4.785) | 5.764 (4.899) | 7.217 (5.072) | 5.803 (4.270) | 6.020 (4.525) | 7.356 (4.655) |
| SCL Anticipation Untreated Criticism Recall | -3.930 (11.052) | -6.200 (12.360) | -7.292 (7.990) | 0.366 (12.029) | -3.415 (10.029) | -2.641 (11.208) | -7.698 (10.246) | -5.794 (9.817) | -5.323 (10.069) | -5.435 (15.715) | -5.534 (14.405) | -3.775 (13.262) |
| SCL Anticipation Untreated Criticism Reinstatement | -5.810 (10.101) | -5.825 (13.657) | -5.579 (6.991) | -5.740 (9.430) | -7.843 (7.636) | -4.105 (9.239) | -8.496 (9.169) | -7.384 (10.063) | -7.687 (9.833) | -8.654 (11.466) | -11.236 (9.170) | -6.241 (9.040) |
| SCL Anticipation Untreated Criticism Renewal | -4.244 (10.956) | -6.765 (11.866) | -6.792 (8.046) | -7.732 (8.977) | -9.456 (10.713) | -6.665 (10.613) | -8.412 (12.929) | -9.854 (13.288) | -6.487 (12.961) | -8.778 (12.516) | -8.892 (9.133) | -5.063 (12.628) |
| SCL Hotspot Future Criticism | -4.826 (10.503) | -2.459 (16.628) | 3.310 (14.585) | 7.984 (4.816) | 7.691 (4.766) | 9.341 (5.504) | 6.847 (4.833) | 5.601 (4.582) | 6.333 (4.792) | 6.030 (4.588) | 5.826 (4.322) | 7.179 (4.528) |
| Rating of Anticipation Phase Treated Criticism Arousal | 4.483 (1.850) | 4.783 (2.140) | 4.150 (2.150) | 3.167 (1.637) | 3.533 (1.742) | 3.467 (2.025) | 2.867 (1.843) | 2.817 (1.812) | 3.050 (1.895) | 2.983 (1.954) | 3.050 (1.927) | 3.117 (1.994) |
| Rating of Anticipation Phase Treated Criticism Disgust | 2.300 (1.715) | 2.567 (2.020) | 2.400 (1.877) | 1.317 (0.650) | 1.317 (0.866) | 1.817 (1.658) | 1.467 (0.787) | 1.483 (0.725) | 1.783 (1.119) | 1.517 (1.156) | 1.817 (1.669) | 1.600 (0.914) |
| Rating of Anticipation Phase Treated Criticism Fear | 3.833 (1.704) | 4.200 (2.307) | 3.800 (2.184) | 2.167 (1.220) | 2.117 (1.072) | 2.267 (1.369) | 1.900 (1.148) | 2.000 (1.259) | 2.267 (1.670) | 1.917 (1.287) | 2.367 (1.987) | 2.117 (1.685) |
| Rating of Anticipation Phase Treated Criticism Guilt | 3.767 (2.377) | 3.700 (2.674) | 3.767 (1.897) | 1.633 (0.890) | 1.817 (0.905) | 2.367 (1.345) | 1.967 (1.217) | 2.200 (1.535) | 2.433 (1.518) | 1.817 (1.102) | 2.217 (1.855) | 2.283 (1.472) |
| Rating of Anticipation Phase Treated Criticism Sadness | 3.867 (1.781) | 4.083 (2.421) | 3.500 (1.592) | 1.817 (0.951) | 2.100 (0.875) | 2.250 (1.376) | 1.883 (1.208) | 1.900 (1.037) | 2.150 (1.226) | 1.850 (1.043) | 2.417 (1.503) | 1.967 (1.106) |
| Rating of Anticipation Phase Treated Criticism Anger | 2.850 (1.844) | 2.733 (1.547) | 2.783 (1.700) | 1.417 (0.720) | 1.433 (0.653) | 1.817 (1.477) | 1.333 (0.547) | 1.717 (0.935) | 1.733 (1.048) | 1.500 (1.383) | 1.783 (1.529) | 1.700 (0.887) |
| Rating of Anticipation Phase Future Criticism Anger | 4.500 (2.312) | 4.783 (2.288) | 4.293 (2.072) | 1.567 (1.015) | 1.800 (1.111) | 1.621 (1.023) | 1.383 (0.739) | 1.983 (1.679) | 2.069 (1.811) | 1.333 (0.711) | 1.900 (1.206) | 1.724 (1.107) |
| Rating of Anticipation Phase Future Criticism Arousal | 5.517 (1.714) | 6.000 (2.009) | 4.897 (2.465) | 4.633 (2.076) | 4.667 (2.023) | 4.328 (1.718) | 3.517 (2.156) | 4.050 (1.859) | 3.431 (2.034) | 3.400 (1.918) | 3.817 (1.817) | 3.483 (1.902) |
| Rating of Anticipation Phase Future Criticism Disgust | 2.917 (1.948) | 3.800 (2.712) | 3.879 (2.538) | 1.300 (0.551) | 1.817 (1.417) | 1.690 (1.411) | 1.283 (0.715) | 1.983 (1.709) | 1.690 (1.398) | 1.267 (0.612) | 1.883 (1.442) | 1.741 (1.099) |
| Rating of Anticipation Phase Future Criticism Fear | 5.517 (1.976) | 5.433 (2.140) | 5.345 (2.468) | 3.400 (2.032) | 3.650 (2.052) | 2.897 (1.844) | 2.717 (1.770) | 2.967 (1.852) | 2.810 (1.595) | 2.467 (1.795) | 2.800 (1.627) | 2.690 (1.391) |
| Rating of Anticipation Phase Future Criticism Guilt | 6.083 (2.142) | 5.717 (2.542) | 6.448 (1.703) | 2.283 (1.794) | 2.800 (1.896) | 1.948 (1.298) | 2.283 (1.735) | 2.400 (1.511) | 2.000 (1.376) | 1.750 (1.216) | 2.117 (1.230) | 2.276 (1.405) |
| Rating of Anticipation Phase Future Criticism Sadness | 6.283 (1.804) | 6.250 (2.281) | 6.793 (1.730) | 1.750 (1.057) | 2.433 (1.530) | 1.759 (1.162) | 1.733 (1.324) | 2.383 (1.827) | 1.966 (1.620) | 1.633 (1.231) | 2.150 (1.146) | 1.897 (1.145) |
| Rating of Anticipation Phase Untreated Criticism Anger | 2.908 (1.333) | 2.608 (1.148) | 2.842 (1.461) | 1.433 (0.682) | 1.567 (0.691) | 1.875 (1.567) | 1.400 (0.687) | 1.833 (0.925) | 1.825 (1.107) | 1.525 (1.323) | 1.767 (1.023) | 1.600 (0.762) |
| Rating of Anticipation Phase Untreated Criticism Arousal | 4.825 (1.355) | 4.783 (1.875) | 4.167 (1.877) | 3.642 (1.498) | 3.775 (1.705) | 3.558 (1.936) | 3.092 (1.771) | 2.883 (1.850) | 2.925 (1.751) | 3.075 (1.917) | 3.042 (1.669) | 3.017 (1.907) |
| Rating of Anticipation Phase Untreated Criticism Disgust | 2.100 (1.330) | 2.558 (1.624) | 2.492 (1.659) | 1.300 (0.651) | 1.358 (0.748) | 1.783 (1.653) | 1.442 (0.736) | 1.642 (0.848) | 1.783 (1.276) | 1.425 (0.777) | 1.717 (1.226) | 1.592 (0.819) |
| Rating of Anticipation Phase Untreated Criticism Fear | 3.833 (1.554) | 4.167 (1.849) | 3.833 (1.739) | 2.242 (1.125) | 2.383 (1.100) | 2.250 (1.256) | 2.008 (1.088) | 2.117 (1.264) | 2.217 (1.388) | 1.842 (0.977) | 2.183 (1.600) | 2.142 (1.406) |
| Rating of Anticipation Phase Untreated Criticism Guilt | 3.700 (1.626) | 3.600 (1.845) | 4.017 (1.271) | 1.767 (0.928) | 2.150 (1.090) | 2.242 (1.233) | 1.958 (1.124) | 2.217 (1.405) | 2.142 (1.203) | 1.792 (0.896) | 2.075 (1.446) | 2.208 (1.282) |
| Rating of Anticipation Phase Untreated Criticism Sadness | 3.808 (1.304) | 3.958 (1.645) | 3.692 (1.442) | 1.842 (0.786) | 2.308 (0.993) | 2.225 (1.195) | 1.892 (1.039) | 2.192 (1.037) | 2.042 (1.071) | 1.767 (0.785) | 2.408 (1.153) | 1.900 (1.043) |
| Rating of Hotspot Phase Treated Criticism Anger | 4.300 (2.575) | 4.733 (2.766) | 4.100 (2.468) | 2.767 (2.029) | 2.833 (1.949) | 2.867 (2.209) | 2.633 (2.008) | 2.933 (2.288) | 2.933 (1.929) | 2.733 (2.532) | 2.533 (1.776) | 2.767 (2.208) |
| Rating of Hotspot Phase Treated Criticism Arousal | 5.367 (2.220) | 6.200 (2.074) | 4.567 (2.648) | 4.367 (2.109) | 4.667 (2.354) | 4.500 (2.301) | 4.233 (2.373) | 4.067 (2.532) | 3.900 (2.496) | 4.067 (2.420) | 4.067 (2.434) | 3.800 (2.511) |
| Rating of Hotspot Phase Treated Criticism Disgust | 3.200 (2.441) | 3.667 (2.928) | 3.667 (2.657) | 1.900 (1.709) | 1.967 (1.608) | 2.667 (2.294) | 2.367 (2.109) | 2.300 (1.725) | 2.700 (2.215) | 2.133 (1.756) | 1.967 (1.542) | 2.300 (1.878) |
| Rating of Hotspot Phase Treated Criticism Fear | 5.667 (2.057) | 5.633 (2.798) | 5.333 (2.783) | 3.433 (2.388) | 3.200 (2.124) | 3.300 (2.087) | 3.567 (2.501) | 3.267 (2.599) | 3.000 (2.319) | 3.067 (2.490) | 2.667 (1.953) | 3.000 (2.150) |
| Rating of Hotspot Phase Treated Criticism Guilt | 5.933 (2.638) | 5.667 (2.987) | 6.300 (2.168) | 3.433 (2.223) | 3.133 (2.285) | 3.967 (2.312) | 3.833 (2.692) | 3.567 (2.885) | 3.900 (2.683) | 3.467 (2.675) | 3.100 (2.591) | 3.600 (2.207) |
| Rating of Hotspot Phase Treated Criticism Sadness | 6.300 (2.366) | 5.933 (2.532) | 6.533 (1.995) | 3.967 (2.157) | 3.800 (2.041) | 3.567 (1.995) | 3.833 (2.705) | 3.800 (2.295) | 3.733 (2.149) | 3.467 (2.596) | 3.300 (2.037) | 3.233 (2.144) |
| Rating of Hotspot Phase Future Criticism Anger | 4.500 (2.312) | 4.776 (2.328) | 4.293 (2.072) | 3.233 (2.192) | 3.724 (2.153) | 4.138 (2.083) | 3.200 (2.325) | 3.483 (2.262) | 3.966 (2.291) | 3.033 (2.059) | 3.483 (1.993) | 3.276 (1.907) |
| Rating of Hotspot Phase Future Criticism Arousal | 5.517 (1.714) | 6.034 (2.035) | 4.897 (2.465) | 5.400 (2.328) | 5.793 (2.144) | 5.414 (1.659) | 4.633 (2.371) | 4.931 (2.170) | 4.897 (2.498) | 4.467 (2.209) | 4.690 (2.020) | 4.690 (2.480) |
| Rating of Hotspot Phase Future Criticism Disgust | 2.917 (1.948) | 3.741 (2.741) | 3.879 (2.538) | 2.067 (1.660) | 3.103 (2.425) | 2.897 (2.144) | 2.033 (1.497) | 2.897 (2.006) | 2.931 (2.389) | 2.200 (1.627) | 3.207 (2.555) | 2.759 (2.294) |
| Rating of Hotspot Phase Future Criticism Fear | 5.517 (1.976) | 5.362 (2.142) | 5.345 (2.468) | 4.067 (2.532) | 4.655 (2.395) | 3.448 (2.443) | 3.367 (2.385) | 3.724 (2.520) | 3.793 (2.411) | 3.067 (2.449) | 3.793 (2.610) | 3.621 (2.651) |
| Rating of Hotspot Phase Future Criticism Guilt | 6.083 (2.142) | 5.690 (2.582) | 6.448 (1.703) | 4.500 (2.764) | 5.000 (2.507) | 4.690 (2.674) | 4.033 (2.735) | 3.793 (2.426) | 4.483 (2.668) | 3.567 (2.763) | 3.414 (2.612) | 4.379 (2.513) |
| Rating of Hotspot Phase Future Criticism Sadness | 6.283 (1.804) | 6.207 (2.309) | 6.793 (1.730) | 4.233 (2.239) | 4.828 (1.983) | 4.448 (2.384) | 3.633 (2.297) | 3.966 (2.427) | 3.966 (2.398) | 3.467 (2.345) | 3.966 (2.228) | 3.655 (2.622) |
| Rating of Hotspot Phase Future Criticism Anger | 4.500 (2.312) | 4.776 (2.328) | 4.293 (2.072) | 3.233 (2.192) | 3.724 (2.153) | 4.138 (2.083) | 3.200 (2.325) | 3.483 (2.262) | 3.966 (2.291) | 3.033 (2.059) | 3.483 (1.993) | 3.276 (1.907) |
| Rating of Hotspot Phase Future Criticism Arousal | 5.517 (1.714) | 6.034 (2.035) | 4.897 (2.465) | 5.400 (2.328) | 5.793 (2.144) | 5.414 (1.659) | 4.633 (2.371) | 4.931 (2.170) | 4.897 (2.498) | 4.467 (2.209) | 4.690 (2.020) | 4.690 (2.480) |
| Rating of Hotspot Phase Future Criticism Disgust | 2.917 (1.948) | 3.741 (2.741) | 3.879 (2.538) | 2.067 (1.660) | 3.103 (2.425) | 2.897 (2.144) | 2.033 (1.497) | 2.897 (2.006) | 2.931 (2.389) | 2.200 (1.627) | 3.207 (2.555) | 2.759 (2.294) |
| Rating of Hotspot Phase Future Criticism Fear | 5.517 (1.976) | 5.362 (2.142) | 5.345 (2.468) | 4.067 (2.532) | 4.655 (2.395) | 3.448 (2.443) | 3.367 (2.385) | 3.724 (2.520) | 3.793 (2.411) | 3.067 (2.449) | 3.793 (2.610) | 3.621 (2.651) |
| Rating of Hotspot Phase Future Criticism Guilt | 6.083 (2.142) | 5.690 (2.582) | 6.448 (1.703) | 4.500 (2.764) | 5.000 (2.507) | 4.690 (2.674) | 4.033 (2.735) | 3.793 (2.426) | 4.483 (2.668) | 3.567 (2.763) | 3.414 (2.612) | 4.379 (2.513) |
| Rating of Hotspot Phase Future Criticism Sadness | 6.283 (1.804) | 6.207 (2.309) | 6.793 (1.730) | 4.233 (2.239) | 4.828 (1.983) | 4.448 (2.384) | 3.633 (2.297) | 3.966 (2.427) | 3.966 (2.398) | 3.467 (2.345) | 3.966 (2.228) | 3.655 (2.622) |
| Rating of Hotspot Phase Untreated Criticism Anger | 4.700 (2.602) | 4.833 (2.520) | 4.400 (2.283) | 2.767 (2.063) | 3.000 (1.531) | 3.333 (2.309) | 3.033 (2.109) | 3.033 (2.025) | 3.200 (2.250) | 2.700 (2.322) | 3.033 (2.025) | 2.667 (2.171) |
| Rating of Hotspot Phase Untreated Criticism Arousal | 5.667 (1.729) | 5.800 (2.250) | 5.133 (2.609) | 4.667 (2.354) | 5.033 (2.428) | 4.967 (2.189) | 4.533 (2.583) | 4.167 (2.437) | 4.333 (2.578) | 4.100 (2.631) | 4.133 (2.161) | 4.000 (2.841) |
| Rating of Hotspot Phase Untreated Criticism Disgust | 2.633 (2.205) | 3.933 (2.828) | 3.967 (2.785) | 1.800 (1.400) | 2.300 (1.896) | 2.967 (2.341) | 2.500 (2.224) | 2.733 (2.083) | 3.033 (2.371) | 2.333 (2.202) | 2.633 (2.125) | 2.800 (2.091) |
| Rating of Hotspot Phase Untreated Criticism Fear | 5.367 (2.553) | 5.233 (2.359) | 5.267 (2.753) | 3.633 (2.539) | 3.633 (2.484) | 3.633 (2.456) | 3.400 (2.554) | 3.000 (2.378) | 3.500 (2.529) | 2.633 (2.442) | 2.700 (2.215) | 3.033 (2.498) |
| Rating of Hotspot Phase Untreated Criticism Guilt | 6.233 (2.192) | 5.767 (2.849) | 6.567 (2.112) | 3.900 (2.426) | 4.100 (2.683) | 4.400 (2.222) | 3.767 (2.825) | 3.533 (2.837) | 4.200 (2.483) | 3.267 (2.559) | 3.300 (2.423) | 3.833 (2.291) |
| Rating of Hotspot Phase Untreated Criticism Sadness | 6.267 (2.196) | 6.567 (2.254) | 6.900 (1.788) | 4.067 (2.545) | 4.600 (2.298) | 4.433 (1.906) | 3.833 (2.718) | 3.767 (2.635) | 4.000 (1.948) | 3.633 (2.566) | 4.067 (2.377) | 3.667 (2.368) |
| Beck Depression Inventory | 15.179 (6.092) | 11.885 (5.233) | 14.250 (6.421) | 10.893 (7.937) | 10.962 (5.950) | 9.714 (7.138) | 10.821 (6.673) | 9.808 (5.622) | 11.750 (8.095) | 8.464 (6.904) | 9.346 (7.099) | 10.643 (9.366) |
| Frost Multidimensional Perfectionism Scale | 3.093 (0.649) | 3.260 (0.554) | 3.157 (0.600) | 3.000 (0.666) | 3.109 (0.564) | 3.142 (0.601) | 3.112 (0.634) | 3.135 (0.584) | 3.148 (0.591) | 3.087 (0.732) | 3.060 (0.634) | 3.084 (0.659) |
| Leibowitz Social Anxiety Scale | 2.003 (0.503) | 2.056 (0.448) | 1.998 (0.495) | 1.999 (0.469) | 2.039 (0.407) | 2.002 (0.516) | 1.988 (0.518) | 1.990 (0.405) | 1.967 (0.557) | 1.927 (0.543) | 2.014 (0.446) | 1.922 (0.546) |
| Performance Failure Appraisale Inventory (Fear of Failure scale) | 118.485 (7.159) | 120.710 (8.642) | 120.280 (12.575) | 108.061 (22.218) | 114.774 (19.815) | 107.080 (27.394) | 108.394 (25.707) | 115.097 (21.481) | 111.720 (20.223) | 103.364 (29.056) | 113.161 (22.632) | 108.520 (22.437) |
| Pure Procrastination Scale | 2.877 (1.051) | 2.978 (0.955) | 2.709 (0.952) | 2.783 (1.025) | 3.067 (0.996) | 2.782 (0.848) | 2.857 (1.026) | 2.926 (0.987) | 2.742 (0.841) | 2.830 (1.082) | 2.892 (1.012) | 2.820 (0.933) |
| Salivary Alpha Amylase | 140.527 (91.230) | 111.418 (76.090) | 139.882 (89.356) | 138.194 (91.869) | 101.254 (70.448) | 126.414 (90.184) |  |  |  |  |  |  |
| PTSD DSM-V Scale | 0.437 (0.516) | 0.696 (0.952) | 0.512 (0.739) |  |  |  |  |  |  | 0.321 (0.694) | 0.291 (0.544) | 0.256 (0.567) |
| General Anxiety Disorder | 2.064 (0.545) | 1.859 (0.569) | 1.922 (0.616) |  |  |  |  |  |  | 1.636 (0.601) | 1.574 (0.467) | 1.691 (0.431) |
| Panic Disorder DSM-V Scale | 1.279 (0.346) | 1.315 (0.391) | 1.404 (0.606) |  |  |  |  |  |  | 1.161 (0.409) | 1.111 (0.189) | 1.235 (0.320) |
| Yale-Brown Obsessive-Compulsive Scale | 13.607 (5.928) | 12.815 (6.379) | 10.391 (5.639) |  |  |  |  |  |  | 9.893 (7.632) | 11.370 (5.732) | 10.304 (7.486) |

### Supplementary Table 2. Descriptive data Per Protocol Mean (SD)

| **Analysis** | **ImRs>IE Pre>Post-Treatment** | | **ImRs>IE Pre-Treatment>3-m F-up** | | **ImRs>IE Pre-Treatment>6-m F-up** | | **ImRs-DSR>ImRs Pre>Post-Treatment** | | **ImRs-DSR>ImRs Pre-Treatment>3-m F-up** | | **ImRs-DSR>ImRs Pre-Treatment>6-m F-up** | |
| --- | --- | --- | --- | --- | --- | --- | --- | --- | --- | --- | --- | --- |
|  | **t (df)** | **p** | **t (df)** | **p** | **t (df)** | **p** | **t (df)** | **p** | **t (df)** | **p** | **t (df)** | **p** |
| SCL Anticipation Treated Criticism Recall | -1.525 (df=103) | 0.13 | -0.316 (df=103) | 0.753 | -0.652 (df=103) | 0.516 | 1.410 (df=103) | 0.162 | 2.535 (df=103) | 0.013 | 1.379 (df=103) | 0.171 |
| SCL Anticipation Treated Criticism Reinstatement | -1.552 (df=79) | 0.125 | -1.566 (df=79) | 0.121 | -2.305 (df=79) | 0.024 | -0.186 (df=79) | 0.853 | 0.312 (df=79) | 0.756 | 0.147 (df=79) | 0.883 |
| SCL Anticipation Treated Criticism Renewal | -0.616 (df=98) | 0.539 | -0.815 (df=98) | 0.417 | -1.280 (df=98) | 0.204 | 1.414 (df=98) | 0.161 | 1.549 (df=98) | 0.125 | 1.041 (df=98) | 0.301 |
| SCL Hotspot Treated Criticism Reinstatement | -1.173 (df=76) | 0.245 | -1.210 (df=76) | 0.23 | -1.279 (df=76) | 0.205 | -0.211 (df=76) | 0.833 | 0.724 (df=76) | 0.472 | 0.124 (df=76) | 0.901 |
| SCL Anticipation Untreated Criticism Recall | -0.442 (df=103) | 0.66 | 1.137 (df=103) | 0.258 | 0.544 (df=103) | 0.588 | -0.541 (df=103) | 0.59 | -0.422 (df=103) | 0.674 | -0.708 (df=103) | 0.48 |
| SCL Anticipation Untreated Criticism Reinstatement | -0.549 (df=83) | 0.584 | 0.288 (df=83) | 0.774 | -0.752 (df=83) | 0.454 | -0.910 (df=83) | 0.366 | 0.139 (df=83) | 0.89 | -1.379 (df=83) | 0.172 |
| SCL Anticipation Untreated Criticism Renewal | 0.221 (df=98) | 0.826 | 0.286 (df=98) | 0.775 | 0.652 (df=98) | 0.516 | -0.738 (df=98) | 0.462 | -0.852 (df=98) | 0.396 | -0.986 (df=98) | 0.326 |
| SCL Anticipation Future Criticism | -1.131 (df=85) | 0.261 | -1.691 (df=85) | 0.095 | -1.125 (df=85) | 0.264 | 0.715 (df=85) | 0.477 | 0.777 (df=85) | 0.439 | 0.704 (df=85) | 0.483 |
| SCL Hotspot Future Criticism | -0.719 (df=85) | 0.474 | -0.967 (df=85) | 0.336 | -0.715 (df=85) | 0.476 | 1.104 (df=85) | 0.273 | 1.337 (df=85) | 0.185 | 1.219 (df=85) | 0.226 |
| Salivary Alpha Amylase | -0.826 (df=123) | 0.411 | 0.330 (df=123) | 0.742 |  |  |  |  |  |  |  |  |
| Rating of Anticipation Phase Treated Criticism Arousal | 0.111 (df=87) | 0.912 | -0.540 (df=87) | 0.59 | -0.359 (df=87) | 0.72 | -0.943 (df=87) | 0.348 | -1.338 (df=87) | 0.184 | -1.078 (df=87) | 0.284 |
| Rating of Anticipation Phase Treated Criticism Disgust | -0.618 (df=87) | 0.538 | -0.567 (df=87) | 0.572 | 0.072 (df=87) | 0.943 | -1.544 (df=87) | 0.126 | -1.058 (df=87) | 0.293 | 0.108 (df=87) | 0.914 |
| Rating of Anticipation Phase Treated Criticism Guilt | 0.443 (df=87) | 0.659 | 0.527 (df=87) | 0.599 | 0.764 (df=87) | 0.447 | -0.856 (df=87) | 0.394 | -0.293 (df=87) | 0.77 | 9.483×10-16 (df=87) | 1 |
| Rating of Anticipation Phase Treated Criticism Sadness | 0.147 (df=87) | 0.883 | -0.410 (df=87) | 0.683 | 0.691 (df=87) | 0.491 | -1.619 (df=87) | 0.109 | -1.710 (df=87) | 0.091 | -0.263 (df=87) | 0.793 |
| Rating of Anticipation Phase Treated Criticism Anger | 0.277 (df=87) | 0.783 | 1.105 (df=87) | 0.272 | 0.729 (df=87) | 0.468 | -0.692 (df=87) | 0.491 | 0.074 (df=87) | 0.941 | 0.243 (df=87) | 0.809 |
| Rating of Anticipation Phase Future Criticism Anger | -0.086 (df=86) | 0.931 | 0.461 (df=86) | 0.646 | 0.448 (df=86) | 0.655 | -0.533 (df=86) | 0.596 | -0.830 (df=86) | 0.409 | -0.493 (df=86) | 0.623 |
| Rating of Anticipation Phase Future Criticism Arousal | -0.759 (df=86) | 0.45 | 0.073 (df=86) | 0.942 | -0.102 (df=86) | 0.919 | -1.278 (df=86) | 0.205 | -0.704 (df=86) | 0.483 | -1.165 (df=86) | 0.247 |
| Rating of Anticipation Phase Future Criticism Disgust | -0.635 (df=86) | 0.527 | -0.286 (df=86) | 0.776 | -0.436 (df=86) | 0.664 | 0.354 (df=86) | 0.724 | 0.576 (df=86) | 0.566 | 0.359 (df=86) | 0.72 |
| Rating of Anticipation Phase Future Criticism Fear | 0.483 (df=86) | 0.63 | 0.505 (df=86) | 0.615 | 0.651 (df=86) | 0.516 | 0.956 (df=86) | 0.342 | 0.102 (df=86) | 0.919 | 0.034 (df=86) | 0.973 |
| Rating of Anticipation Phase Future Criticism Guilt | 1.499 (df=86) | 0.137 | 0.765 (df=86) | 0.446 | 1.181 (df=86) | 0.241 | 2.664 (df=86) | 0.009 | 1.775 (df=86) | 0.079 | 0.914 (df=86) | 0.363 |
| Rating of Anticipation Phase Future Criticism Sadness | 1.332 (df=86) | 0.186 | 1.132 (df=86) | 0.261 | 0.947 (df=86) | 0.346 | 2.245 (df=86) | 0.027 | 1.579 (df=86) | 0.118 | 1.360 (df=86) | 0.177 |
| Rating of Anticipation Phase Untreated Criticism Anger | 1.161 (df=87) | 0.249 | 2.168 (df=87) | 0.033 | 1.449 (df=87) | 0.151 | -0.201 (df=87) | 0.841 | 0.714 (df=87) | 0.477 | 1.070 (df=87) | 0.288 |
| Rating of Anticipation Phase Untreated Criticism Arousal | 0.343 (df=87) | 0.733 | -0.312 (df=87) | 0.756 | 0.015 (df=87) | 0.988 | -0.783 (df=87) | 0.436 | -1.233 (df=87) | 0.221 | -1.068 (df=87) | 0.289 |
| Rating of Anticipation Phase Untreated Criticism Disgust | -1.195 (df=87) | 0.235 | -0.756 (df=87) | 0.452 | -0.436 (df=87) | 0.664 | -1.469 (df=87) | 0.145 | -0.610 (df=87) | 0.544 | 0.153 (df=87) | 0.879 |
| Rating of Anticipation Phase Untreated Criticism Fear | -0.444 (df=87) | 0.658 | -0.493 (df=87) | 0.623 | 0.017 (df=87) | 0.987 | -0.463 (df=87) | 0.645 | -0.949 (df=87) | 0.345 | -0.579 (df=87) | 0.564 |
| Rating of Anticipation Phase Untreated Criticism Guilt | 1.206 (df=87) | 0.231 | 0.833 (df=87) | 0.407 | 0.855 (df=87) | 0.395 | 0.811 (df=87) | 0.419 | 1.142 (df=87) | 0.256 | 0.632 (df=87) | 0.529 |
| Rating of Anticipation Phase Untreated Criticism Sadness | 0.933 (df=87) | 0.353 | 0.406 (df=87) | 0.686 | 1.230 (df=87) | 0.222 | -0.540 (df=87) | 0.59 | -0.316 (df=87) | 0.753 | 0.605 (df=87) | 0.547 |
| Rating of Hotspot Phase Treated Criticism Anger | -0.560 (df=87) | 0.577 | -0.172 (df=87) | 0.864 | -0.807 (df=87) | 0.422 | -1.018 (df=87) | 0.311 | -0.818 (df=87) | 0.415 | -1.105 (df=87) | 0.272 |
| Rating of Hotspot Phase Treated Criticism Arousal | -0.778 (df=87) | 0.439 | -1.407 (df=87) | 0.163 | -1.116 (df=87) | 0.267 | -2.139 (df=87) | 0.035 | -2.063 (df=87) | 0.042 | -1.831 (df=87) | 0.071 |
| Rating of Hotspot Phase Treated Criticism Disgust | -0.711 (df=87) | 0.479 | -0.810 (df=87) | 0.42 | -0.984 (df=87) | 0.328 | -1.245 (df=87) | 0.217 | -0.607 (df=87) | 0.545 | -0.518 (df=87) | 0.606 |
| Rating of Hotspot Phase Treated Criticism Fear | -0.319 (df=87) | 0.751 | -0.387 (df=87) | 0.699 | -0.543 (df=87) | 0.588 | -0.638 (df=87) | 0.525 | -0.048 (df=87) | 0.961 | -0.938 (df=87) | 0.351 |
| Rating of Hotspot Phase Treated Criticism Guilt | -0.054 (df=87) | 0.957 | 1.314×10-15 (df=87) | 1 | -0.140 (df=87) | 0.889 | -0.325 (df=87) | 0.746 | 0.409 (df=87) | 0.684 | 0.186 (df=87) | 0.853 |
| Rating of Hotspot Phase Treated Criticism Sadness | 0.330 (df=87) | 0.742 | 0.497 (df=87) | 0.621 | 0.293 (df=87) | 0.77 | 1.375 (df=87) | 0.173 | 0.993 (df=87) | 0.323 | 0.977 (df=87) | 0.331 |
| Rating of Hotspot Phase Future Criticism Anger | 0.326 (df=85) | 0.745 | 0.009 (df=85) | 0.992 | 0.265 (df=85) | 0.792 | -1.350 (df=85) | 0.181 | -1.312 (df=85) | 0.193 | -0.417 (df=85) | 0.678 |
| Rating of Hotspot Phase Future Criticism Arousal | -0.190 (df=85) | 0.85 | -0.308 (df=85) | 0.759 | -0.419 (df=85) | 0.677 | -1.146 (df=85) | 0.255 | -1.530 (df=85) | 0.13 | -1.602 (df=85) | 0.113 |
| Rating of Hotspot Phase Future Criticism Disgust | 0.348 (df=85) | 0.729 | 0.057 (df=85) | 0.955 | 0.254 (df=85) | 0.8 | 0.560 (df=85) | 0.577 | 0.152 (df=85) | 0.88 | 0.810 (df=85) | 0.42 |
| Rating of Hotspot Phase Future Criticism Fear | 0.951 (df=85) | 0.344 | 0.690 (df=85) | 0.492 | 1.129 (df=85) | 0.262 | 1.509 (df=85) | 0.135 | -0.115 (df=85) | 0.909 | 0.197 (df=85) | 0.844 |
| Rating of Hotspot Phase Future Criticism Guilt | 1.250 (df=85) | 0.215 | 0.196 (df=85) | 0.845 | 0.311 (df=85) | 0.757 | 1.482 (df=85) | 0.142 | 0.087 (df=85) | 0.931 | -0.265 (df=85) | 0.792 |
| Rating of Hotspot Phase Future Criticism Sadness | 1.038 (df=85) | 0.302 | 0.560 (df=85) | 0.577 | 0.754 (df=85) | 0.453 | 1.481 (df=85) | 0.142 | 0.797 (df=85) | 0.428 | 1.165 (df=85) | 0.247 |
| Rating of Hotspot Phase Untreated Criticism Anger | 0.177 (df=87) | 0.86 | -0.208 (df=87) | 0.836 | 0.317 (df=87) | 0.752 | -1.355 (df=87) | 0.179 | -0.936 (df=87) | 0.352 | -0.106 (df=87) | 0.916 |
| Rating of Hotspot Phase Untreated Criticism Arousal | 0.376 (df=87) | 0.708 | -0.716 (df=87) | 0.476 | -0.142 (df=87) | 0.887 | -0.966 (df=87) | 0.337 | -1.193 (df=87) | 0.236 | -0.760 (df=87) | 0.45 |
| Rating of Hotspot Phase Untreated Criticism Disgust | -1.331 (df=87) | 0.187 | -1.619 (df=87) | 0.109 | -1.515 (df=87) | 0.133 | -1.054 (df=87) | 0.295 | -0.405 (df=87) | 0.687 | -0.202 (df=87) | 0.84 |
| Rating of Hotspot Phase Untreated Criticism Fear | 0.236 (df=87) | 0.814 | -0.355 (df=87) | 0.723 | 0.273 (df=87) | 0.786 | 0.059 (df=87) | 0.953 | -0.622 (df=87) | 0.536 | -0.409 (df=87) | 0.683 |
| Rating of Hotspot Phase Untreated Criticism Guilt | 1.043 (df=87) | 0.3 | 0.309 (df=87) | 0.758 | 0.706 (df=87) | 0.482 | 0.782 (df=87) | 0.436 | 0.176 (df=87) | 0.86 | 0.377 (df=87) | 0.707 |
| Rating of Hotspot Phase Untreated Criticism Sadness | 0.417 (df=87) | 0.678 | -0.554 (df=87) | 0.581 | 0.198 (df=87) | 0.844 | 0.893 (df=87) | 0.374 | 0.151 (df=87) | 0.88 | 1.089 (df=87) | 0.279 |
| Beck Depression Inventory | 1.737 (df=79) | 0.086 | 1.198 (df=79) | 0.235 | 1.807 (df=79) | 0.075 | 1.866 (df=79) | 0.066 | 0.222 (df=79) | 0.825 | 0.462 (df=79) | 0.645 |
| Frost Multidimensional Perfectionism Scale | -0.684 (df=108) | 0.496 | -1.450 (df=108) | 0.15 | -1.780 (df=108) | 0.078 | -1.582 (df=108) | 0.117 | -1.150 (df=108) | 0.253 | -1.146 (df=108) | 0.255 |
| Leibowitz Social Anxiety Scale | -0.244 (df=115) | 0.807 | -0.738 (df=115) | 0.462 | 0.429 (df=115) | 0.668 | -0.389 (df=115) | 0.698 | -0.508 (df=115) | 0.612 | 0.428 (df=115) | 0.669 |
| Performance Failure Appraisal Inventory (Fear of Failure scale) | 0.749 (df=86) | 0.456 | 0.823 (df=86) | 0.413 | 1.251 (df=86) | 0.214 | 1.127 (df=86) | 0.263 | 0.504 (df=86) | 0.615 | 0.647 (df=86) | 0.519 |
| Pure Procrastination Scale | 1.635 (df=137) | 0.104 | -0.251 (df=137) | 0.802 | -0.231 (df=137) | 0.817 | 0.139 (df=137) | 0.889 | -0.669 (df=137) | 0.505 | -1.167 (df=137) | 0.245 |
| PTSD DSM-V Scale | -1.239 (df=75) | 0.219 |  |  |  |  |  |  |  |  | -0.604 (df=75) | 0.548 |
| General Anxiety Disorder | 0.727 (df=75) | 0.47 |  |  |  |  |  |  |  |  | -0.264 (df=75) | 0.793 |
| Panic Disorder DSM-V Scale | -0.574 (df=75) | 0.568 |  |  |  |  |  |  |  |  | -0.217 (df=75) | 0.829 |
| Yale-Brown Obsessive-Compulsive Scale | 1.372 (df=75) | 0.174 |  |  |  |  |  |  |  |  | -0.780 (df=75) | 0.438 |

### Supplementary Table 3 Per Protocol Contrast Analysis
